# Unsymmetrical Thienopentalenes: Synthesis, Optoelectronic Properties, and (Anti)aromaticity Analysis

**DOI:** 10.1021/acsomega.1c05618

**Published:** 2022-03-03

**Authors:** Tamás Gazdag, Péter J. Mayer, Péter Pál Kalapos, Tamás Holczbauer, Ouissam El Bakouri, Gábor London

**Affiliations:** † MTA TTK Lendület Functional Organic Materials Research Group, Institute of Organic Chemistry, Research Centre for Natural Sciences, Magyar tudósok krt. 2., Budapest 1117, Hungary; ‡ Hevesy György PhD School of Chemistry, Eötvös Loránd University, Pázmány Péter sétány 1/a, Budapest 1117, Hungary; § Institute of Chemistry, University of Szeged, Rerrich tér 1, Szeged 6720, Hungary; ∥ Centre for Structural Science and Institute of Organic Chemistry, Research Centre for Natural Sciences, Magyar tudósok körútja 2 Budapest 1117, Hungary; ⊥ Institut de Química Computacional i Catàlisi (IQCC) and Departament de Química, Universitat de Girona, C/Maria Aurèlia Capmany 6, Girona 17003, Catalonia, Spain

## Abstract

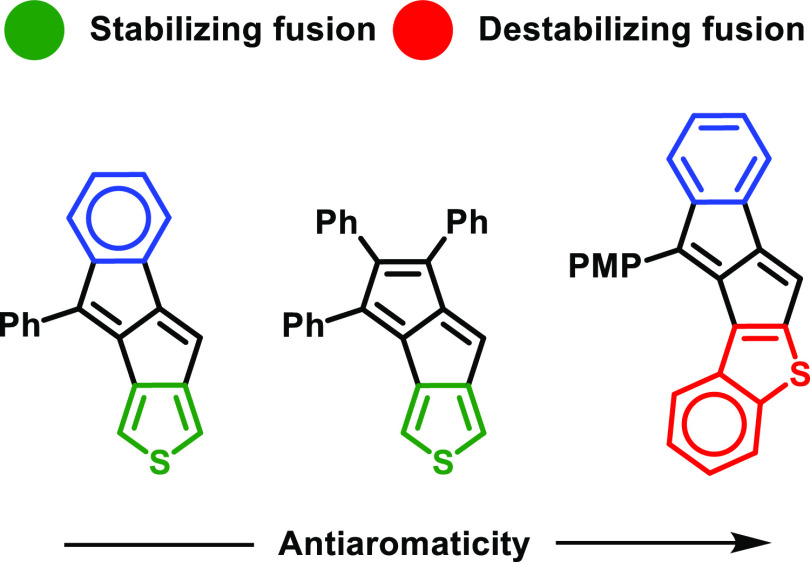

The synthesis and properties of a series of unsymmetrical thienopentalenes are explored, including both monoareno and diareno derivatives. For the synthesis of monoareno pentalenes, a carbopalladation cascade reaction between alkynes and *gem*-dibromoolefins was applied. Diareno pentalene derivatives were accessed via gold-catalyzed cyclization of diynes. Thiophene was fused to pentalene in two different geometries via its 2,3 and 3,4 bonds. 2,3-Fusion resulted in increased antiaromaticity of the pentalene unit compared to the 3,4-fusion both in the monoareno and diareno framework. Monothienopentalenes that contained the destabilizing 2,3-fusion could not be isolated. For diareno derivatives, the aromatic character of the different aryl groups fused to the pentalene was not independent. Destabilizing fusion on one side resulted in alleviated aromaticity on the other side and vice versa. The synthesized molecules were characterized experimentally by ^1^H NMR and UV–vis spectroscopies, cyclic voltammetry, and X-ray crystallography, and their aromatic character was assessed using magnetic (NICS and ACID) and electronic indices (MCI and FLU).

## Introduction

Polycyclic conjugated π-electron systems with incorporated antiaromatic [4*n*]π subunits have been extensively explored recently.^1−8^ The interest in such materials is twofold. From the perspective of the [4*n*]π unit,^9^ these structures provide opportunities to study antiaromatic molecules as π-extension ensures sufficient stabilization of the otherwise unstable [4*n*]π-electron systems.^10−12^ From the perspective of the resulting acene-derivatives, [4*n*]π subunits can improve their stability and processability for organic electronic applications.^13−16^ Hence, exploring the synthesis and properties of novel π-extended antiaromatics is of considerable interest from both fundamental and applications point of view. Among antiaromatic subunits, pentalene is particularly interesting as this planar 8 π-electron molecule could be extended/stabilized through different fusion and substituent patterns, and the synthesis of its derivatives has advanced a lot recently.^2^ However, regardless of the available synthetic methodologies for the preparation of derivatives with unsymmetrical conjugated cores,^17−26^ material properties of linear and symmetric π-extended antiaromatics are explored mostly.^27−37^ Although the study of symmetric polycyclic conjugated systems is facilitated by their more straightforward syntheses in general, the departure from such structures proved to be fruitful in the case of acenes. Within the class of acenes, the introduction of asymmetry, especially by the unsymmetrical fusion of thiophenes, led to improved stability, good solubility, novel assembly modes, and interesting electronic features such as ambipolar transport properties in several cases.^38−42^

Unique properties that may emerge from π-extended thieno-antiaromatics having unsymmetrical conjugated cores, along with the potential of asymmetry to tune electronic properties, prompted us to investigate such structures. In this contribution, we explore the synthesis, basic opto-electronic properties, and aromaticity of unsymmetrical thienopentalenes. Special focus is given to derivatives that have a proton attached to the pentalene unit, which could be an important reporter on the extent of antiaromaticity within the molecules.

## Results and Discussion

### Synthesis

**Scheme 1:**
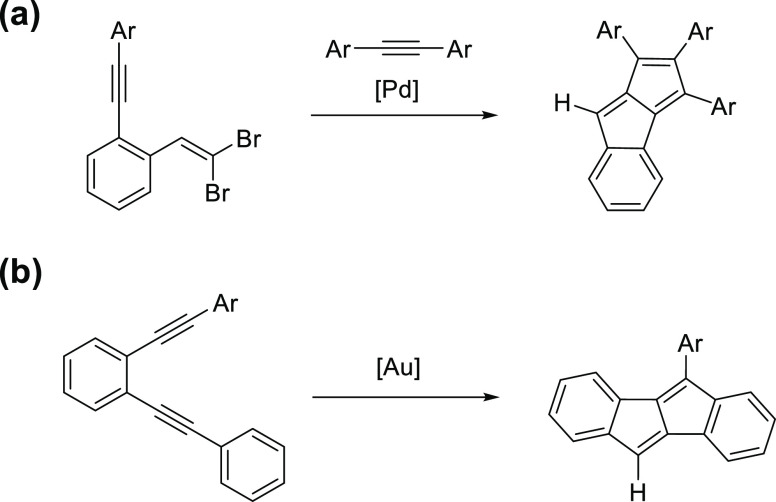
General Strategies Applied for the Syntheses of Molecules in This Study; (a) Pd-Catalyzed and (b) Au-Catalyzed Cascade Transformation

To access unsymmetrical thienopentalenes, we followed two synthetic approaches (Scheme 1), that have been proven to be reliable methods for the synthesis of pentalene derivatives. The methodology developed and optimized by Diederich and co-workers (Scheme 1a)^43,44^ is based on the cascade carbopalladation between alkynes and *gem*-dibromoolefines and leads to monoannelated pentalenes. For the synthesis of unsymmetrical diareno[*a*,*e*]pentalene-type molecules, we turned to the gold-catalyzed cyclization of diynes developed and optimized by Hashmi and co-workers (Scheme 1b).^21^ Both methodologies involve the formation of multiple C–C bonds in one-pot, which should be considered in the evaluation of the product yields.

**Figure 1:**
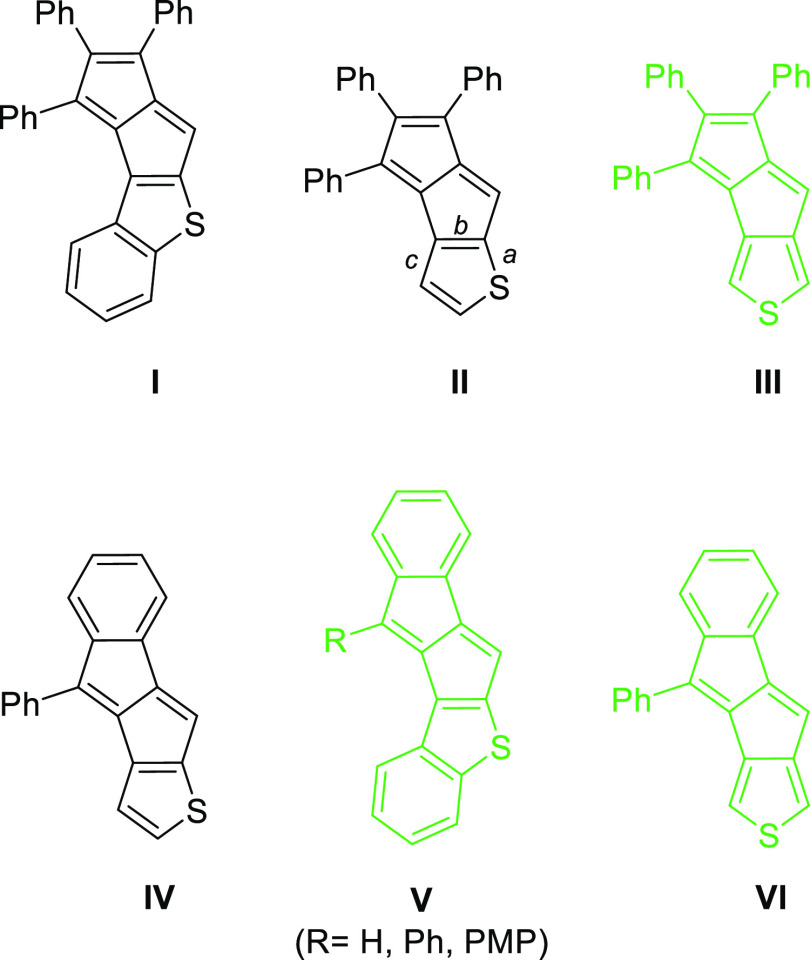
Targeted thienopentalene derivatives. Isolated structures are shown in green (PMP = *p*-methoxyphenyl).

We expected that these methodologies could be adapted to synthesize a series of thieno and benzothieno derivatives of pentalene (Figure 1), taken also into account that thiophene can be annelated both through its 2,3(*b*) and 3,4(*c*) C–C bonds. It is noted, however, that several reports that deal with synthetic methodology development describe benzo[4,5]pentaleno[2,1-*b*]thiophene (**IV** type) derivatives as unstable.^17,19−22^ Hence, instead of **IV**, we explored the synthesis of the benzothiophene-fused benzopentalenes (**V**) only.

**Scheme 2:**
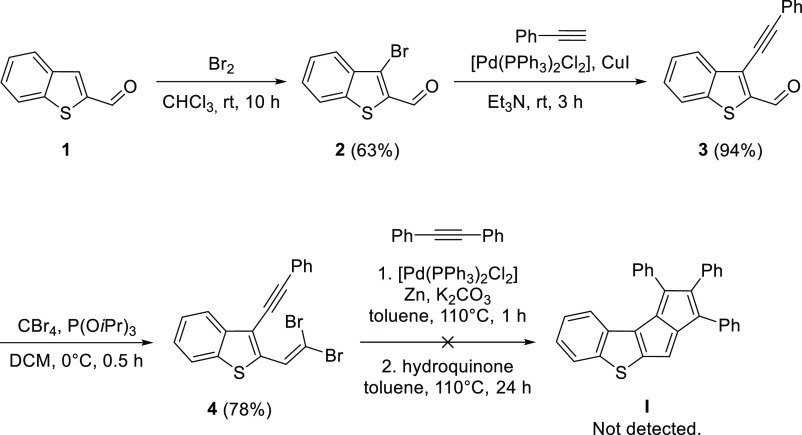
Attempted Synthesis of Compound **I**

First, we attempted the synthesis of thienopentalene **I** (Scheme 2), which was expected to contain the most antiaromatic pentalene unit. The strong antiaromaticity, in this case, comes from the monoannelated structure on one hand and from the strong alkene character of the fused thiophene double bond on the other. The synthesis was started with the bromination of benzo[*b*]thiophene-2-carboxaldehyde **1** followed by a Sonogashira coupling between the brominated derivative **2** and phenylacetylene. Cross-coupling product **3** was converted to the corresponding *gem*-dibromoolefin **4** using the procedure reported by Lautens and co-workers.^45^ The attempted carbopalladation cascade between *gem*-dibromoolefin **4** and diphenylacetylene did not provide the desired product **I**. It is noted that the *gem*-dibromoolefin starting materials were fully consumed during all the cascade carbopalladation reactions in this study, so the lack of isolable products in some cases is most probably not related to the reactivity of these starting materials but rather their non-selective transformations under the reaction conditions.

**Scheme 3:**
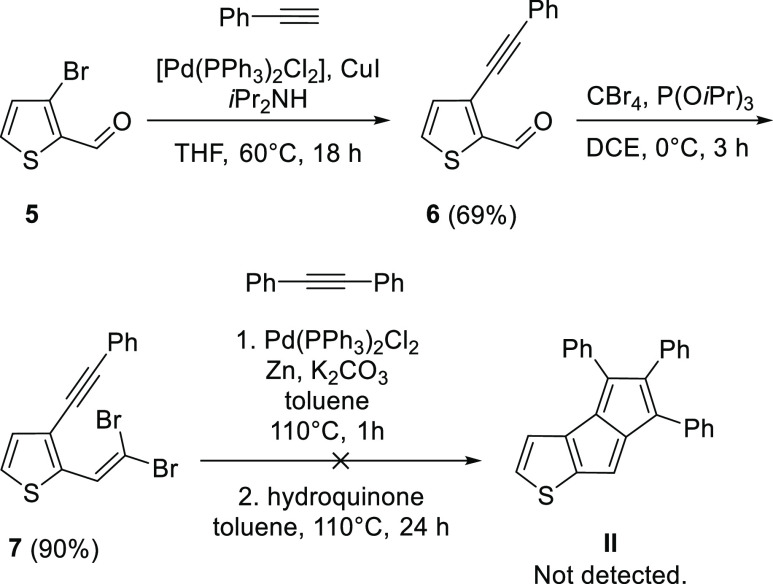
Attempted Synthesis of Compound **II**

The low stability of **I** might be the reason of its unsuccessful synthesis. Thus, we instead targeted thienopentalene **II** (Scheme 3), in which the fused benzothiophene unit was reduced to a single thiophene ring. Although thiophene has a considerable diene character, the bond order of its 2,3(*b*) double bond is somewhat lower compared to the same bond in benzothiophene. This was expected to contribute to increased stability of **II** through somewhat decreased antiaromaticity of the pentalene unit compared to that of **I**.

**Scheme 4:**
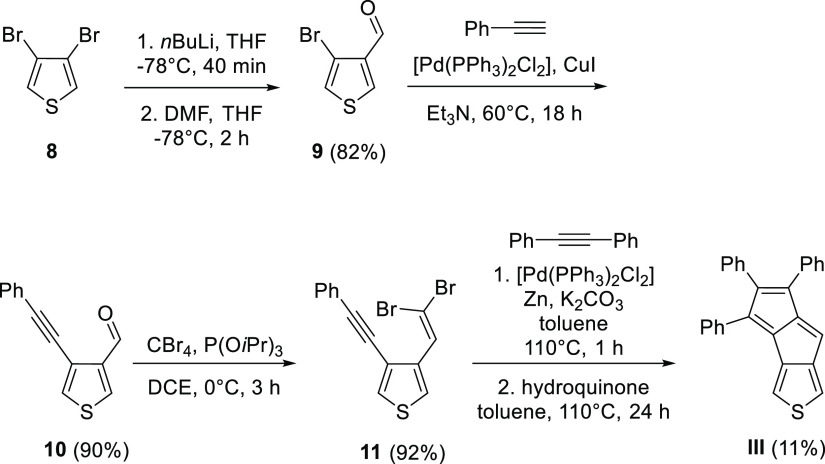
Synthesis of Compound **III**

The attempted synthesis of **II** (Scheme 3) followed a similar route as of **I**. A Sonogashira coupling on 3-bromothiophene-2-carboxaldehyde **5** is followed by the conversion of the aldehyde in **6** into a *gem*-dibromoolefin **7**, which was the starting material for the pentalene-forming cascade reaction. Unfortunately, as in the case of compound **I**, no pentalene formation was detected. It has been shown earlier by Kawase and co-workers that thiophene fusion through its 3,4(*c*) bond strongly reduces the pentalene contribution to the resulting π-system.^32^ Those molecules (pentaleno[1,2-*c*:4,5-*c*′]dithiophene derivatives), in contrast to dibenzo[*a*,*e*]pentalene derivatives, were characterized by allowed highest occupied molecular orbital (HOMO)-to-lowest occupied molecular orbital (LUMO) transitions and a level of fluorescence. A reduced bond order of the fused thiophene bond explains such pronounced changes in properties. Based on the work of Kawase and co-workers, we explored the synthesis of **III** (Scheme 4), where a single thiophene ring is fused to pentalene through its 3,4(*c*) bond. 3,4-Dibromothiophene **8** was used as a starting material, which was lithiated with *n*BuLi and quenched with DMF to access bromoaldehyde **9**. Subsequently, a Sonogashira coupling with phenylacetylene and *gem*-dibromoolefination of the resulting aldehyde **10** led to compound **11**, which could be converted to **III** in 11% yield as a bench-stable green material.

**Scheme 5:**
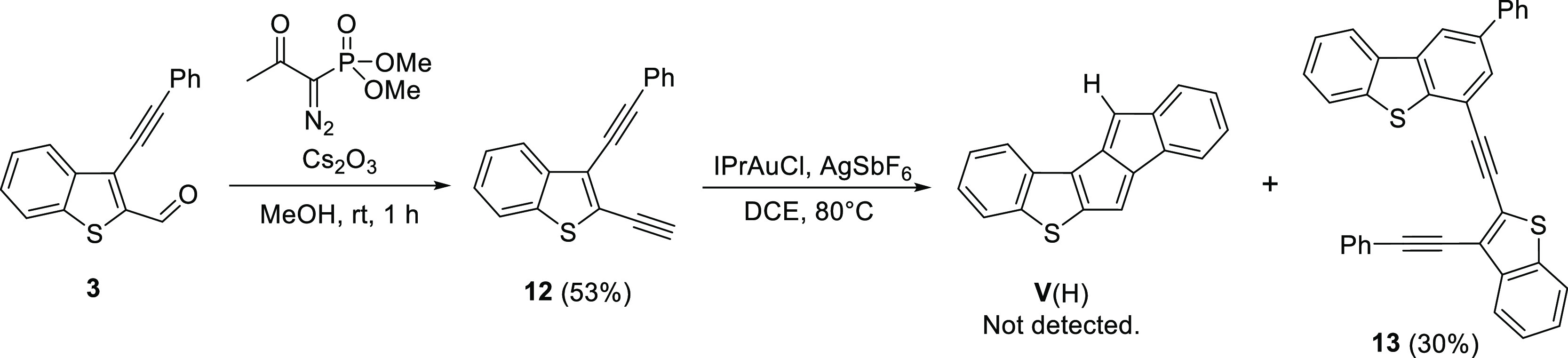
Attempted Synthesis of Compound **V**(H)

Following the monoannelated thienopentalenes, we turned to the synthesis of thiophene containing diarenopentalene derivatives. Compound **3** from the synthesis of **I** was chosen as a starting material to access the non-substituted derivative of **V** [**V**(H)] (Scheme 5) with two pentalene protons in the molecule, which could be useful to evaluate its antiaromaticity with ^1^H NMR spectroscopy. The aldehyde group of **3** could be converted to a terminal alkyne via a Seyferth–Gilbert homologation using the Bestmann–Ohira reagent for the transformation. The resulting diacetylene **12** was submitted to gold-catalyzed pentalene-forming conditions. The gold-catalyzed cascade did not provide the expected **V**(H); instead, a compound was isolated with a structure that was suggested to be **13**. Such dimerized side products have been reported under the applied conditions and were associated with the presence of terminal alkynes.^46^

**Scheme 6:**
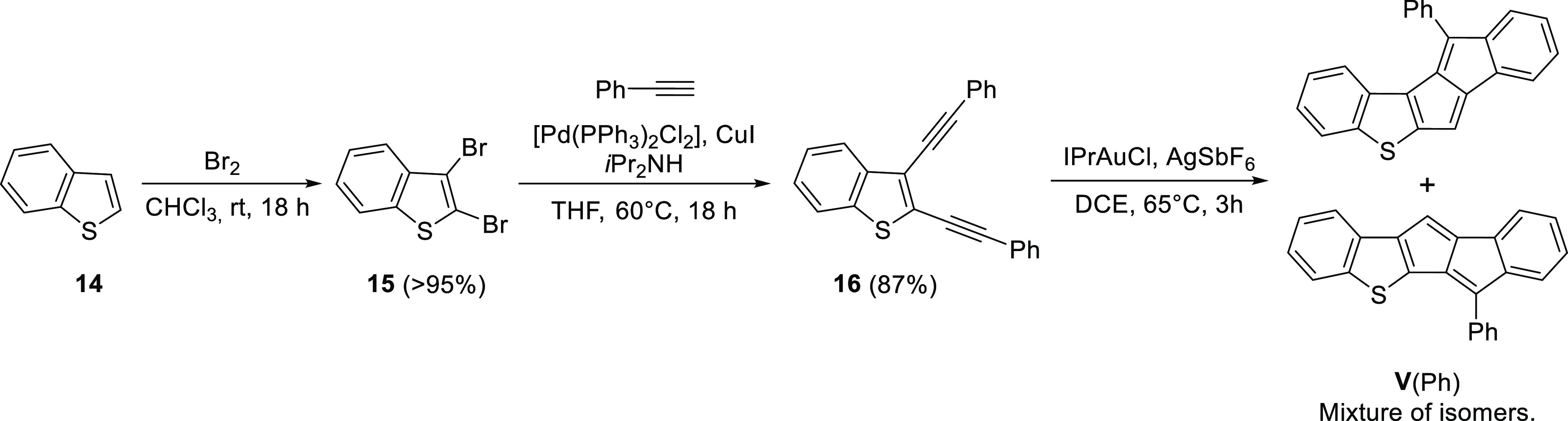
Attempted Synthesis of **V**(Ph)

To eliminate the side reaction, we synthesized a benzothiophene derivative with two phenylacetylene moieties. For this, benzothiophene (**14**) was dibrominated with Br_2_, then product **15** was coupled with two equivalents of phenylacetylene. The gold-catalyzed pentalene formation from diacetylene **16** worked in this case; however, an inseparable and impure mixture of isomers of **V**(Ph) was obtained (Scheme 6).

**Scheme 7:**
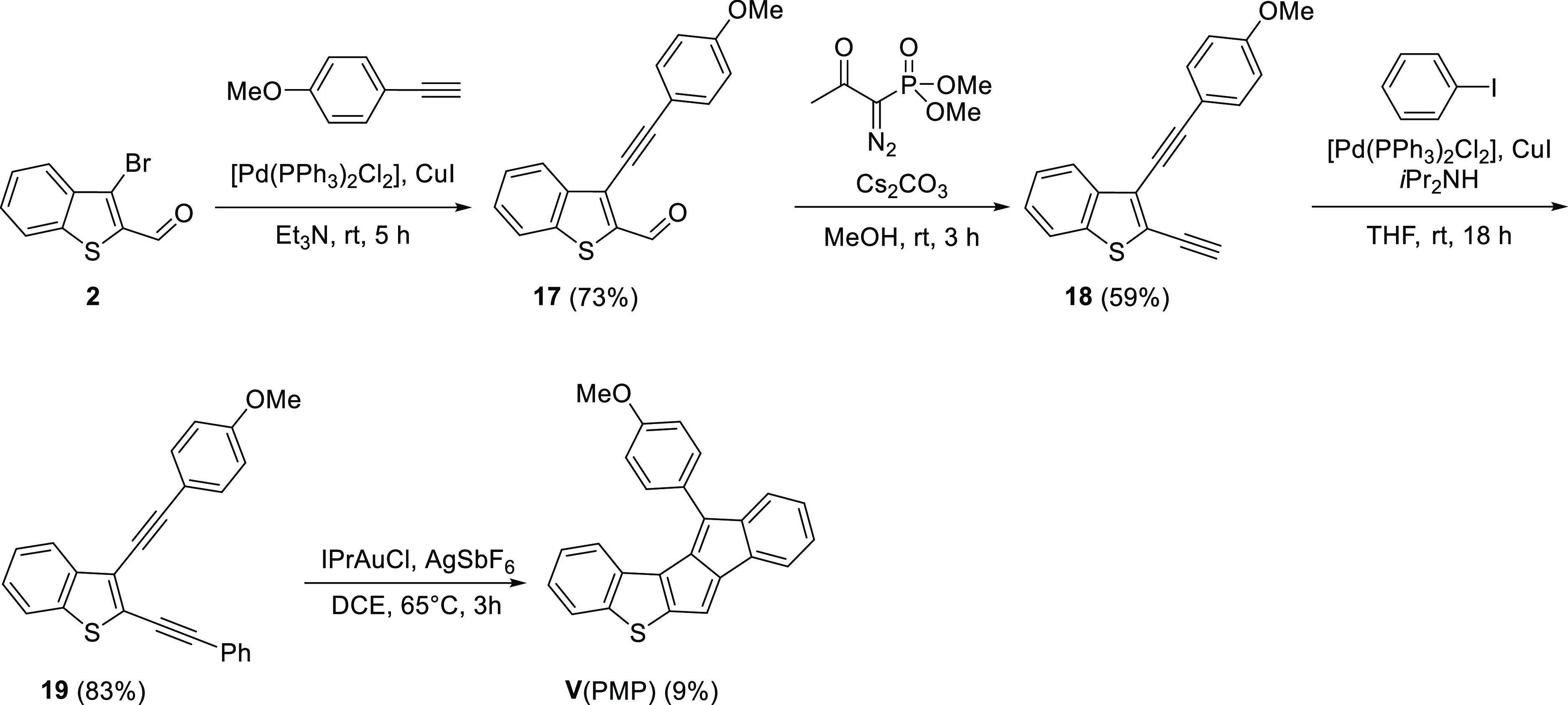
Synthesis of **V**(PMP)

To control the selectivity of the reaction, one of the phenylacetylenes was replaced with 4-ethynylanisole that was expected to promote the regioselective annulation and facilitate the purification (Scheme 7).^47^ Indeed, after performing the gold-catalyzed annulation on compound **19**, a single isomer of **V**(PMP) could be isolated as a green solid.

**Scheme 8:**
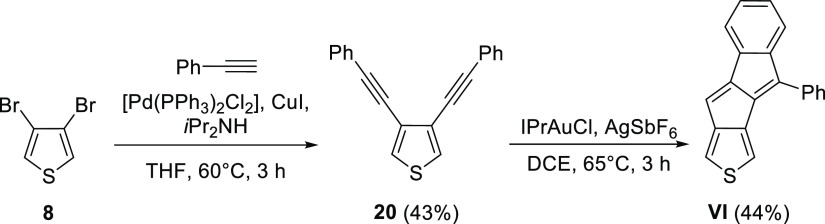
Synthesis of Compound **VI**

We also investigated the synthesis of thienopentalene **VI** (Scheme 8), in which the contribution of pentalene antiaromaticity is expected to be the least of all structures considered in this study due to the strong electronic stabilization. The comparably higher yielding annulation of diacetylene **20**, giving **VI** as an orange solid, seemed to support this speculation.

Although not all the target molecules could be isolated, the unsuccessful syntheses provide important information. The compounds that could not be obtained represent limitations of the applied synthetic methodology on one hand and, likely, the stability of thienopentalene derivatives on the other hand.

### ^1^H NMR Spectroscopy

**Figure 2:**
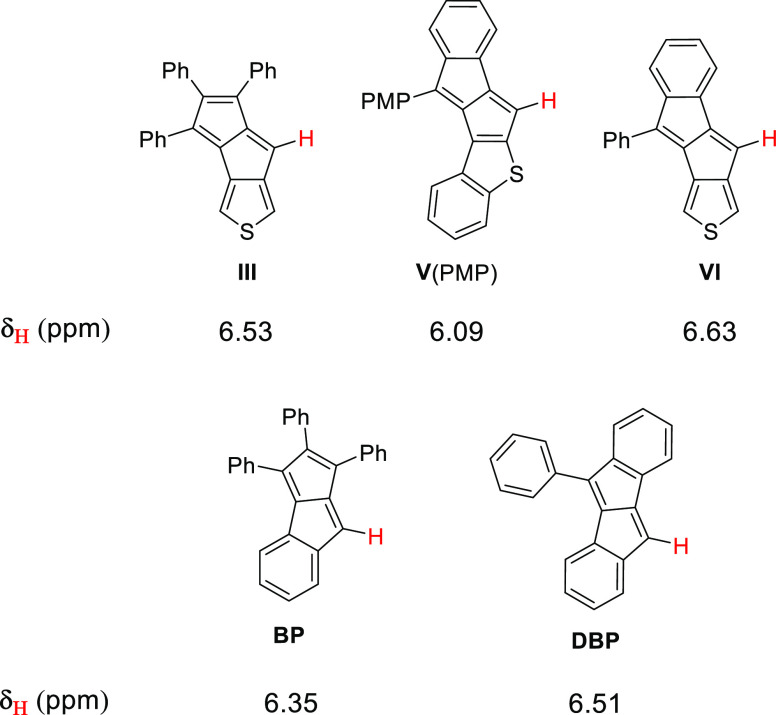
^1^H NMR chemical shifts (CD_2_Cl_2_, 500 MHz, rt) of the protons attached to the pentalene unit within **III**, **V**(PMP), and **VI**. Values for **BP** and **DBP** are added for comparison.

It is instructive to look at the chemical shifts of the protons connected to the pentalene moiety in the isolated molecules (Figure 2). As the substituent patterns around the free pentalene Hs are similar, it allows for the comparison of chemical shifts within the synthesized series of molecules and with related structures that have been reported earlier. In general, paratropic and diatropic ring current effects are reflected in upfield and downfield ^1^H NMR shifts, respectively.^48^

The proton chemical shifts of the pentalene Hs within the synthesized molecules are decreasing in the order of **VI** (6.63 ppm) > **III** (6.53 ppm) > **V**(PMP) (6.09 ppm), which indicate an increasing olefinic double-bond character experienced by the protons of interest; although in **III**, the phenyl substituent could have some influence on the measured chemical shift through additional shielding.

Nevertheless, these values are in line with what would be expected intuitively, based on previously studied structures.^34,49,50^ In both **III** and **VI**, thiophene is fused through its 3,4 bond, which has a low double-bond character that lowers the antiaromatic character of the pentalene subunit. **VI** is further stabilized by the fusion of an additional phenyl ring that leads to an even more alleviated antiaromaticity. Although **V**(PMP) has a stabilizing benzene fusion as well, the other fused aryl system is a benzothiophene, in which the double-bond character of the thiophene ring contributes to the increased antiaromaticity; hence, we call this fusion destabilizing. The chemical shift of the pentalene proton in **III** (6.53 ppm) compared to that of in monobenzopentalene **BP** (6.35 ppm) suggests that thiophene fusion through its 3,4 bond stabilizes the antiaromatic system somewhat stronger than a single phenyl ring. This also holds when **VI** and dibenzopentalene **DBP** are compared. The contribution of benzothiophene fusion in **V**(PMP) to increased antiaromaticity is further confirmed by its analogy with **DBP**.

### X-ray Crystallography

**Figure 3:**
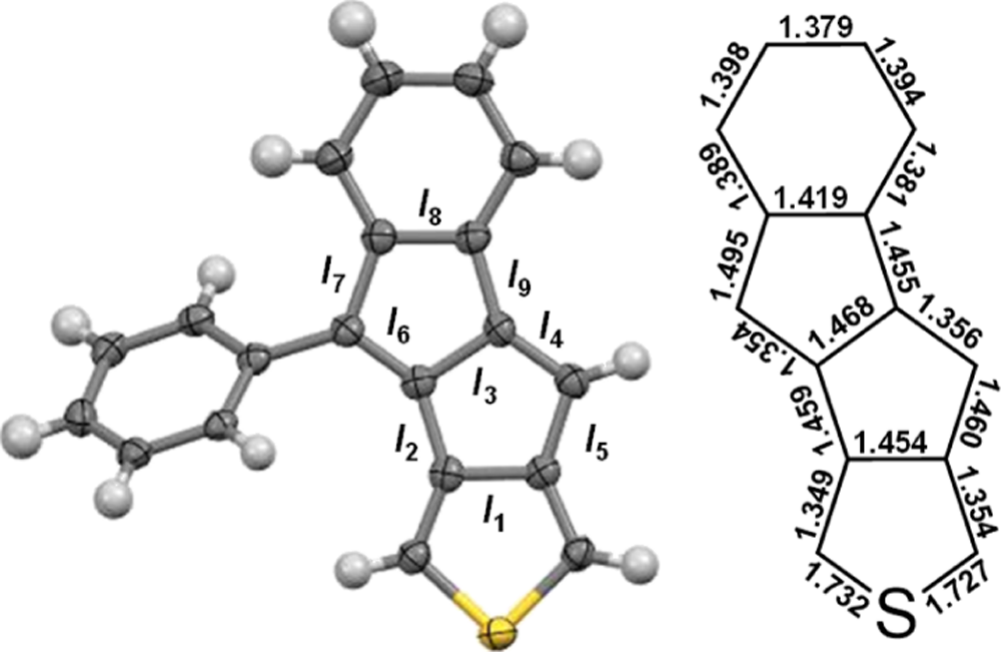
X-ray structure of **VI** (CCDC 2090075) and the corresponding bond lengths (Å) (ORTEP style representation is drawn at the 50% probability level. A disordered phenyl substituent was found in the asymmetric unit. Here, only the dominant conformation is presented, and disorder was omitted for clarity).

Among the synthesized molecules, only **VI** could be crystallized for X-ray single-crystal analysis (Figure 3 and Section S1, Supporting Information). In the asymmetric unit, a whole **VI** molecule was found. Stacks of thienopentalene conjugated cores are built up with π–π interactions within the crystal lattice. The disordered phenyl substituent is found between these stacks in the crystal layer. A general structural feature of antiaromatic molecules is bond length alternation within their conjugated core. This should be observed within the pentalene unit of **VI**, if its antiaromaticity is well preserved. However, by looking at the bond lengths within the molecule, it is clearly not the case. Within the pentalene unit, bond lengths *l*_4_ (1.356 Å) and *l*_6_ (1.354 Å) are considerably shorter than the other bonds; however, no pronounced bond length alternation can be observed that extends to the whole pentalene unit. Based on this, the π-system within the two fused five-membered rings has rather a butadiene-type character (*l*_4_–*l*_3_–*l*_6_), or, if the relatively short *l*_8_ is considered, it shows a fulvene-type conjugation pattern (*l*_3_–*l*_6_–*l*_7_–*l*_8_–*l*_9_–*l*_4_). In any case, based on the crystal structure, there is a relatively low pentalene-type 8π-character present in the molecule. This observation is in line with what was found for symmetric dithieno[*a*,*e*]pentalene derivatives having similarly fused thiophenes.^32,34^

As a comparison, the replacement of a single benzene ring in **DBP** by a thiophene ring in **VI** did not drastically alter the solid-state packing properties of the two compounds. A very similar network of non-covalent interactions within the crystal lattices was found in both cases (Section S1.1, Supporting Information).

### Opto-Electronic Properties

#### UV–Vis Spectroscopy

**Figure 4:**
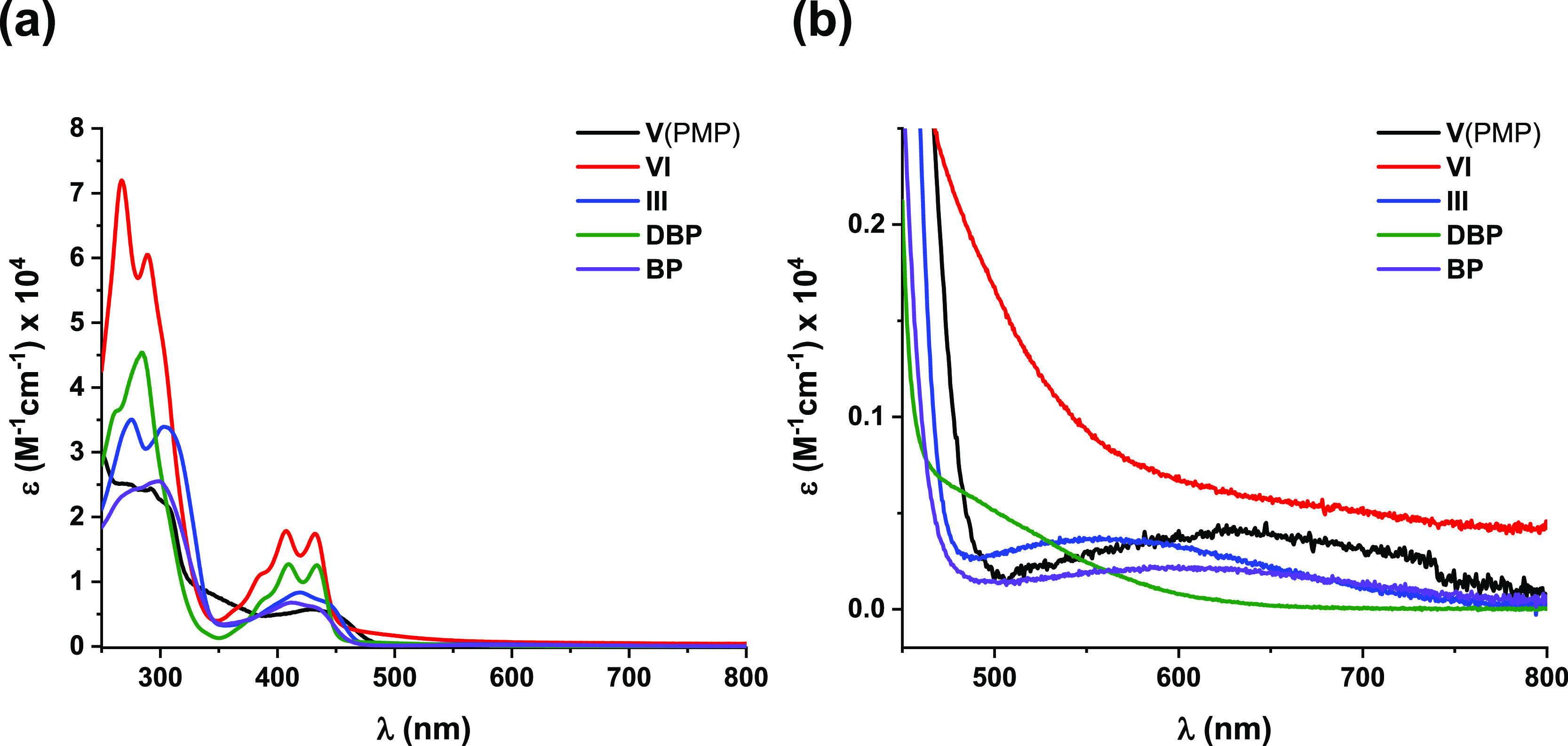
(a) UV–vis spectra of the synthesized new compounds and of **DBP** and **BP** for comparison; (b) partial UV–vis spectra showing the low-intensity, long wavelength absorptions. All spectra were recorded in CHCl_3_.

The optical properties of the synthesized pentalene derivatives were studied by UV–vis spectroscopy (Figure 4) in CHCl_3_. There is no pronounced difference between the compounds in terms of the position of the main absorption bands below 500 nm. The ε for **V**(PMP) in the whole spectrum is comparable to that of **BP**, suggesting that the benzopentalene subunit dominates the optical properties of the molecule regardless of the presence of five fused unsaturated rings. The spectra of **VI** and **DBP** are characterized by two distinct maxima in the 350–450 nm region, which are absent in the spectra of the other molecules. As a signature of the presence of the 8π subunit,^16−19,24,26,30,33−35,43,44,49^ broad, low-intensity absorptions were observed above 500 nm for all compounds measured, although these bands are rather shoulders of the stronger absorption bands below 500 nm in the case of **VI** and **DBP** (note that the spectra obtained for **BP** and **DBP** are added for comparison in Figure 4).

**Figure 5:**
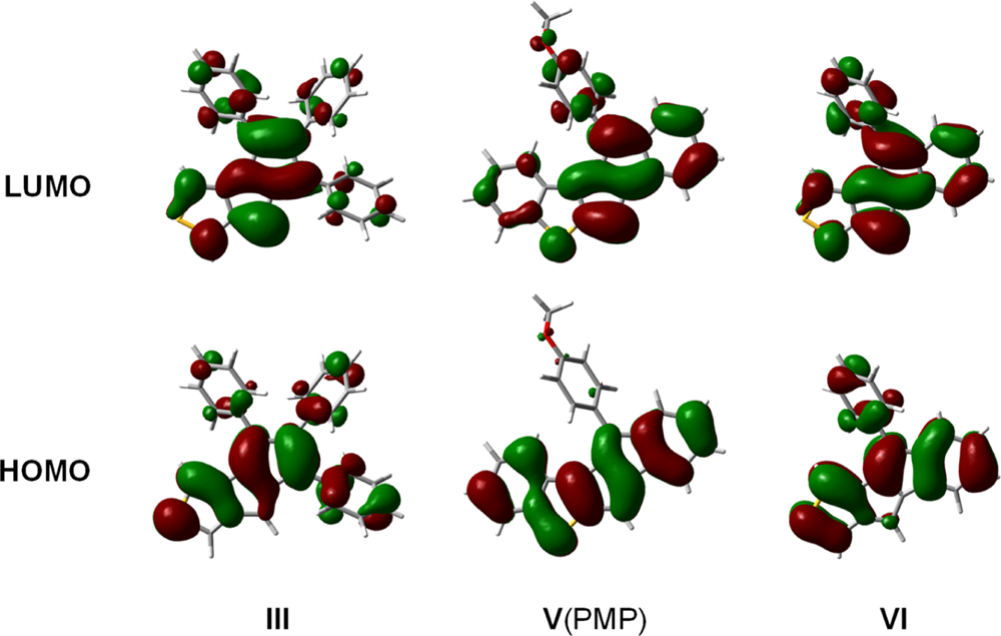
Calculated HOMOs and LUMOs of **III**, **V**(PMP), and **VI** (isosurface of 0.02 a.u. is used).

These long wavelength low-energy bands that are characteristic to pentalenes could be assigned as the symmetry-forbidden HOMO → LUMO transitions,^16−19,24,26,30,33−35,43,44,49^ whereas those in the 350–450 nm region could be assigned as the symmetry-allowed HOMO – 1 → LUMO transitions (Tables S7 and S8, Supporting Information). Clear maxima that could be obtained for **III** (549 nm) and **V**(PMP) (610 nm) correspond to an optical HOMO–LUMO gap of 2.26 and 2.05 eV, respectively (Table 1) (for higher-intensity spectra in this region, see Section S2, Supporting Information). For comparison, **BP** has a maximum approximately at 600 nm and a gap of 2.06 eV. As the HOMO–LUMO gap for **VI** could not be determined based on its UV–Vis spectrum, time-dependent density functional theory calculations [at B3LYP/6-311+G(d,p) level of theory] were performed. In agreement with the UV–vis spectra of **VI** that lacked the clear long-wavelength maximum, theory provided a larger HOMO–LUMO gap of 2.81 eV (441 nm, *f* = 0.015). It is noted that a fulvene-like conjugation pattern has a strong contribution to the HOMO of **VI** (Figure 5), which is in agreement with crystallographic bond lengths within the molecule. Such fulvene-like local structures have been identified in π-extended pentalenes, associated with strongly alleviated antiaromaticity.^44,49^ Furthermore, the pendant phenyl rings have considerable HOMO contribution in the case of **III** and **VI**, while the methoxyphenyl ring has negligible in **V**(PMP). Compared to **VI**, the HOMO of **III** is more pentalene-like, whereas the pentalene contribution is highest in **V**(PMP), which already suggests the extent of antiaromaticity within the series. In all three cases, the pentalene character can be clearly recognized in the LUMO of the molecules.

**Table 1:** Summary of Electrochemical, Optical, and Computational Data for **III**, **V**(PMP), and **VI**

entry	compound	*E*_pa_ (*E*_1/2,ox_) [V]^a^	*E*_pc_ (*E*_1/2,red_) [V]^a^	Δ*E*_redox_ [eV]^b^	Δ*E*_opt_ [eV]	Δ*E*_calc_ [eV]^e^
1	**III**	0.65 (0.60)^c^	–1.68^d^	1.95	2.26	2.25
2	**V**(PMP)	0.54 (0.51)^c^	–1.61 (−1.58)^c^	1.96	2.05	1.99
3	**VI**	0.93^d^	–1.93^d^	2.46	n.d.	2.81

a Electrochemical measurements were carried out in 0.1 M Bu_4_NPF_6_ in dichloromethane at a scan rate of 0.1 V s^–1^ on a platinum wire working electrode. All potentials are given versus the Fc/Fc^+^ couple used as the internal standard (half-wave potentials in parenthesis).

b Estimated from the differences between onset potentials.

c Reversible first reduction or oxidation wave.

d Irreversible first reduction or oxidation wave.

e Calculations were performed on the B3LYP/6-311+G(d,p) level of theory.

#### Electrochemistry

The electrochemical behavior of the synthesized thienopentalene derivatives was examined by cyclic voltammetry (CV) (for further details, see Section S3, Supporting Information). Notably, while for **V**(PMP) both the first oxidation and reduction processes were reversible, for **III**, only the oxidation step was reversible, and both redox processes were irreversible in the case of **VI**. The electrochemical energy gaps change in the order of **V**(PMP) (1.96 V) ≈ **III** (1.95 V) < **VI** (2.46 V) (Table 1). The similar Δ*E*_redox_ values obtained for **V**(PMP) and **III** could partially come from the approximative determination of the onset potentials that are used for the calculations. Among the structures, **VI** can be compared with dibenzo[*a*,*e*]pentalenes and previously reported symmetric dithienopentalenes^32,34^ as it contains the same number of fused rings. The electrochemically determined HOMO–LUMO gap of **VI** (2.46 eV) is almost equal to what has been measured for TMS-substituted dibenzo[*a*,*e*]pentalene (2.48 eV),^34^ while significantly larger than that of pentaleno[1,2-*b*:4,5-*b*′]dithiophenes (1.87–1.97 eV).^34^ The electrochemical gap for pentaleno[1,2-*c*:4,5-*c*′]dithiophenes where both thiophene rings are fused through their 3,4(*c*) bond to the pentalene unit approaches 3 eV,^32^ which is somewhat larger compared to that of **VI**. Similar to pentaleno[1,2-*c*:4,5-*c*′]dithiophene derivatives,^32^**VI** polymerized during the measurements (Figure S16, Supporting Information).

### Aromaticity Analysis

All geometry optimizations were made with the Gaussian 09^51^ package using the B3LYP^52^ hybrid functional and the 6-311+G(d,p)^53^ basis set. The (anti)aromatic character of the studied thienopentalene derivatives was evaluated by means of magnetic (NICS-*XY* scans^54^ and ACID plots^55,56^) and electronic indices (MCI^57^ and FLU^58^) in their ground (S_0_) state, computed at the B3LYP/6-311+G(d,p) level (for further details, see Section S4, Supporting Information). Aromatic rings are characterized by negative NICS values and clockwise ring currents revealed through ACID plots, whereas positive NICS values and anticlockwise ring currents are indicative of antiaromaticity.^48^ The higher the MCI and the lower the FLU, the more aromatic are the rings. For further details, see Section S4 in Supporting Information.

**Figure 6:**
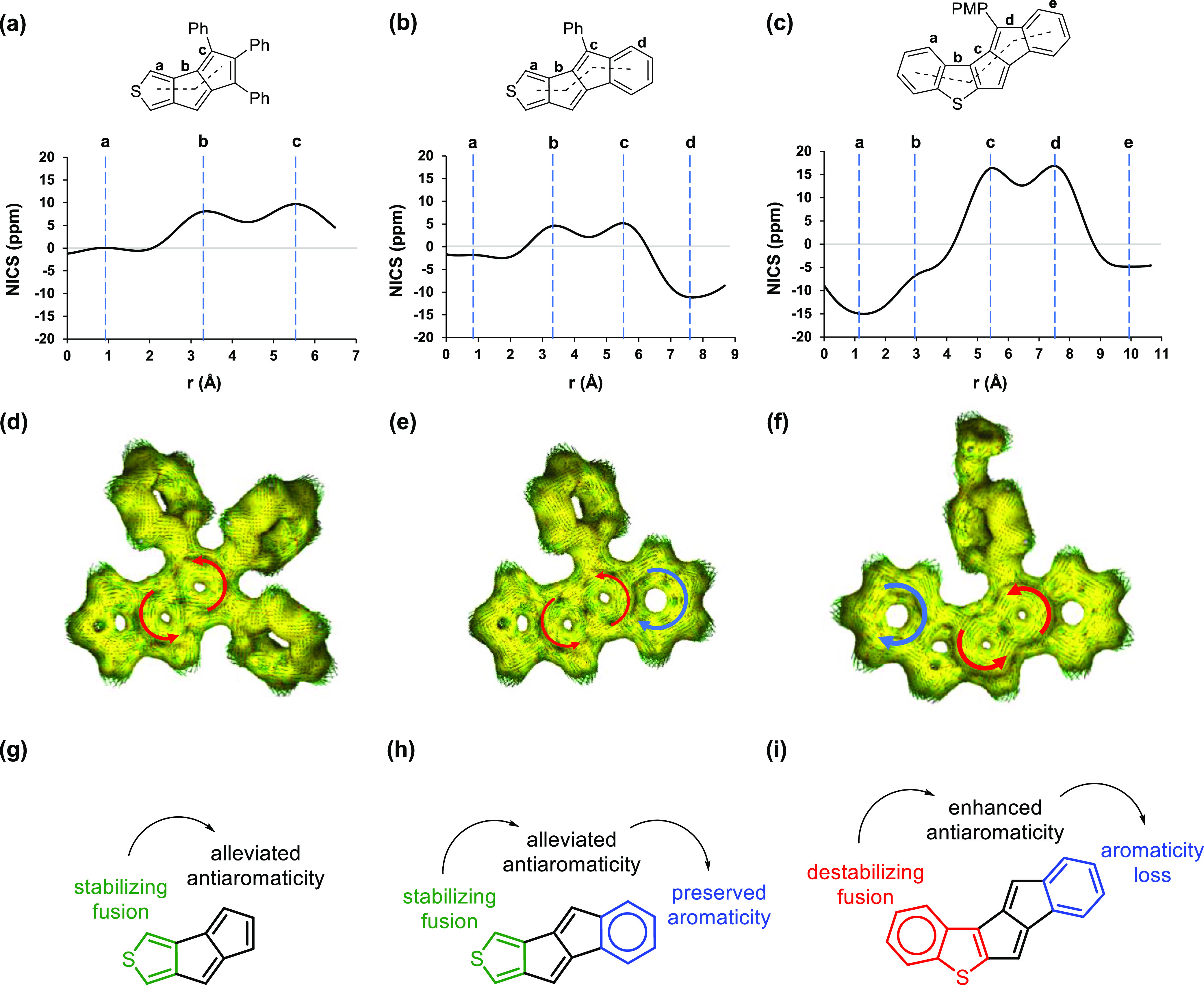
NICS-*XY* scans (a–c) and ACID plots (d–f) of compounds **III**, **VI**, and **V**(PMP) in their ground states. Antiaromaticity changes within the subunits of the molecules (substituents are removed for clarity) are also shown (g–i).

Magnetic-based aromaticity descriptors support the experimental findings. Among the synthesized compounds, based on the experimental data, the pentalene unit in **VI** is characterized by the lowest antiaromaticity, somewhat higher in **III**, while in **V**(PMP), it exhibits the highest. NICS-*XY* scans of the molecules support this trend (Figure 6a–c). Both in **III** and **VI**, the thiophene ring is essentially non-aromatic, although this non-aromaticity resides slightly on the antiaromatic side of the scale in **III** while on the aromatic side in **VI**. In the case of **III**, the small maximum in the scan over the thiophene ring could indicate a weak global paratropic current in the molecule.^59^ The antiaromaticity of the pentalene units is lowest in **VI**, which is stabilized by the 3,4-fusion of the thiophene ring on one hand and by the fused benzene ring on the other hand. In contrast to the non-aromatic thiophene unit, the benzene ring preserved a considerable level of aromaticity. The antiaromaticity of the pentalene unit was found the strongest in **V**(PMP) based on the NICS-*XY* scans. The fusion of a benzothiophene unit contributes to a preserved antiaromaticity due to the strong alkene character of the fused bond in the thiophene ring. This destabilizing interaction is compensated with the preserved aromaticity of the benzene ring within the benzothiophene unit. Likely due to the destabilizing fusion of the benzothiophene, the aromaticity of the benzene ring that is fused on the other side of the pentalene unit is strongly alleviated. This is in contrast to what was observed for **VI**, yet in that case, the thiophene fusion provided strong stabilization for the pentalene unit. Overall, the electronic nature of the fused system on one side of the antiaromatic unit influences the aromaticity of the fused ring on the other side of the same unit (Figure 6g–i). Destabilizing fusion on one side contributes to preserved antiaromaticity of the pentalene unit, which leads to alleviated aromaticity on the other side, such as in **V**(PMP) (Figure 6i). However, stabilizing fusion on one side contributes to alleviated antiaromaticity of the pentalene unit, which leads to preserved aromaticity on the other side, such as in **VI** (Figure 6h). Similar conclusions were found computationally for thiophene-fused benzocyclobutadienes recently.^60^ The possibility of tuning multiple electronic interactions in diarenopentalenes to alleviate antiaromaticity or to preserve aromaticity of the subunits is clearly an advantage that can be exploited in the molecular design. Due to their monoannelated structure, these possibilities are limited for monoarenopentalenes, which contributes to their strongly preserved antiaromaticity on one hand and could limit their synthetic accessibility on the other. The NICS-*XY* scans of the synthetically unfeasible molecules **I**, **II**, and **IV** support this explanation (see Section S4.2, Supporting Information). Complementary to NICS-*XY* scans, ACID plots (Figure 6d–f) reflect the extent of tropicities in each molecule, which is in agreement with the conclusions based on NICS calculations. For the ACID plots of structures **I**, **II**, and **IV**, see Section S4.2 in Supporting Information. The calculated transition energies (Table S7, Supporting Information) support the antiaromaticity trend [**VI** < **III** < **V**(PMP)] revealed by the NICS-*XY* scans, ACID, and MCI (λ_**VI**_(HOMO → LUMO) = 440.86 nm, λ_**III**_(HOMO → LUMO) = 550.36 nm, and λ_**V**(PMP)_ (HOMO → LUMO) = 622.40 nm).

To complement the results of magnetic aromaticity calculations, we also analyzed the molecules by means of electronic indices. The antiaromaticity trend revealed by MCI data is in line with the results obtained from NICS-*XY* scans. The antiaromaticity of the pentalene unit is similar in **III** and **VI** (MCI = 0.0008 e and 0.0007 e, respectively), whereas it is enhanced in **V**(PMP) (MCI = 0.0004 e). As a comparison, pentalene itself has an MCI value of −0.005 e (for comparative FLU and DI analyses of the synthesized and proposed molecules, see Section S4.2 in Supporting Information).

## Summary

In summary, we explored the synthesis and opto-electronic properties of unsymmetrical thienopentalenes. From a synthetic point, these molecules provided a ground for testing and exploiting recently developed methodologies in the preparation of novel pentalene derivatives. We showed that both the cascade carbopalladation reaction and the gold-catalyzed cyclization of diynes is a reliable methodology for the synthesis of unsymmetrical thienopentalenes. Notably, the stability of monoareno pentalenes reached its limit upon the destabilizing (2,3-) fusion of (benzo)thiophene (structures **I** and **II**). Stabilizing (3,4-) fusion of a single thiophene ring to pentalene (compound **III**) led to considerable alleviation of its antiaromaticity. In the case of the unsymmetrical diareno derivatives, we found that the nature of the (benzo)thiophene fusion on one edge of the pentalene unit affects the aromaticity of the fused benzene ring on the other edge. Destabilizing benzothiophene fusion resulted in aromaticity loss in the fused benzene ring (compound **V**) while stabilizing fusion of thiophene resulted in preserved aromaticity in the benzene unit (compound **IV**). In this latter case, due to the extensive stabilization of the pentalene unit, its antiaromaticity is essentially diminished and exhibited a diene/fulvene character.

## Experimental Section

### General Information

Commercial reagents, solvents, and catalysts (Aldrich, Fluorochem, VWR) were purchased as reagent grade and used without further purification. Solvents for extraction or column chromatography were of technical quality. Organic solutions were concentrated by rotary evaporation at 25–40 °C. Thin-layer chromatography (TLC) was carried out on SiO_2_-layered aluminum plates (60778-25EA, Fluka). Column chromatography was performed using SiO_2_-60 (230–400-mesh ASTM, 0.040–0.063 mm from Merck) at 25 °C or using a Teledyne Isco CombiFlash Rf^+^ automated flash chromatographer with silica gel (25–40 μm, Zeochem). Room temperature refers to 25(±1) °C. NMR spectra were acquired on a Varian 500 NMR spectrometer, running at 500 and 126 MHz for ^1^H and ^13^C, respectively. The residual solvent peaks were used as the internal reference. Chemical shifts (δ) are reported in ppm. The following abbreviations are used to indicate the multiplicity in ^1^H NMR spectra: s, singlet; d, doublet; t, triplet; q, quartet; h, heptet; and m, multiplet. ^13^C NMR spectra were acquired on a broad-band decoupled mode. Gas chromatography–mass spectrometry (GC–MS) analysis was performed on a Shimadzu GCMS-QP2010 UltraSystem operated in the electron impact ionization mode. High-resolution measurements were performed on a Sciex TripleTOF 5600+ high-resolution tandem mass spectrometer equipped with a DuoSpray ion source. Atmospheric-pressure chemical ionization was applied in the positive ion detection mode. Samples were dissolved in acetonitrile and flow injected into the acetonitrile/water 1:1 flow. The flow rate was 0.2 mL/min. The resolution of the mass spectrometer was 35000. UV–vis spectra were acquired on a Jasco V-750 spectrophotometer. Melting point was determined visually with a Boetius PHMK-05 hot stage microscope (VEB Kombinat Nagema, Germany).

### General Procedures

#### General Procedure for Gem-dibromoolefination (**GP1**)

The aldehyde (1 equiv) and CBr_4_ (1.5 equiv) were stirred at 0 °C in CH_2_Cl_2_ (10 mL/mmol) under an inert atmosphere (N_2_). A solution of P(O*i*Pr)_3_ (3 equiv) in CH_2_Cl_2_ was added dropwise to the mixture, and the reaction was stirred at 0 °C for 1–6 h. After the reaction was completed, the mixture was diluted with CH_2_Cl_2_ and washed with water twice, then the organic phase was dried on MgSO_4_. The solvent was evaporated under reduced pressure, and the product was purified with column chromatography (SiO_2_; *n*-hexane/EtOAc 12:1).

#### General Procedure for the Carbopalladation Cascade Reaction (**GP2**)

The dibromoolefin (0.22 mmol, 1 equiv), diphenylacetylene (200 mg, 1.10 mmol, 5 equiv), K_2_CO_3_ (61 mg, 0.44 mmol, 2 equiv), Pd(PPh_3_)_2_Cl_2_ (16 mg, 23 μmol 0.1 equiv), Zn powder (20 mg, 30 μmol, 1.4 equiv), and toluene (4 mL) were added to a sealed vial and stirred under an inert atmosphere for 1 h at 110 °C in an aluminum heating block. The reaction was cooled to rt, hydroquinone (30 mg, 0.27 mmol, 1.2 equiv) was added to the reaction mixture, and it was stirred at 110 °C for additional 16 h. After the reaction was completed, the mixture was diluted with EtOAc and washed with water twice and brine once. The organic phase was dried over MgSO_4_. The solvent was evaporated under reduced pressure, and the products were purified by column chromatography (SiO_2_; *n*-hexane/EtOAc, 12:1).

#### General Procedure for Seyferth–Gilbert Homologation (**GP3**)

The aldehyde (1 equiv) and Cs_2_CO_3_ (4 equiv) were stirred in methanol (10 mL/mmol) under a N_2_ atmosphere at rt. The Bestmann–Ohira reagent (1.5 equiv) was added dropwise to the reaction, which was stirred for 1–5 h at rt. The reaction was monitored by TLC. After the reaction was completed, the solvent was evaporated, and the product was purified with column chromatography (SiO_2_; *n*-hexane/EtOAc 12:1).

##### Synthesis of 3-Bromobenzo[*b*]thiophene-2-carbaldehyde (**2**)^61,62^

Bromine (1.33 g, 428 μL, 8.32 mmol) in CHCl_3_ (2 mL) was added dropwise to a stirred, ice cold solution of benzo[*b*]thiophene-2-carbaldehyde (**1**) (1.35 g, 8.32 mmol) in CHCl_3_ (10 mL). After the addition, the solution was allowed to warm to rt and was stirred for 10 h. The mixture was diluted with CHCl_3_, washed once with water, twice with 1 M NaOH, and once with brine. The organic phase was dried over MgSO_4_. The solvent was evaporated under reduced pressure. The crude product was recrystallized from acetic acid (4 mL). The product was obtained as pale yellow crystals. Yield: 1.25 g, 5.18 mmol, 63%. ^1^H NMR (500 MHz, CDCl_3_): δ 10.29 (s, 1H), 8.02 (d, *J* = 8.0 Hz, 1H), 7.87 (d, *J* = 8.0 Hz, 1H), 7.55 (dt, *J* = 14.9, 7.2 Hz, 2H) ppm; ^13^C{^1^H} NMR (126 MHz, CDCl_3_): δ 184.8, 140.6, 138.2, 136.7, 129.4, 126.1, 125.2, 123.6, 118.9 ppm; HRMS (APCI) *m*/*z*: [M + H]^+^ calcd for C_9_H_6_OSBr^+^, 240.9317; found, 240.9314.

##### Synthesis of 3-(Phenylethynyl)benzo[*b*]thiophene-2-carbaldehyde (**3**)^63^

3-Bromobenzo[*b*]thiophene-2-carbaldehyde (**2**) (300 mg, 1.24 mmol), phenylacetylene (152 mg, 164 μL, 1.5 mmol), Pd(PPh_3_)_2_Cl_2_ (16.4 mg, 23.4 μmol), and CuI (7 mg, 37 μmol) were stirred under an inert atmosphere in Et_3_N (10 mL) at rt for 3 h. After the reaction was completed, the mixture was diluted with EtOAc and washed once with water, twice with 10% HCl, and once with brine. The organic phase was dried over MgSO_4_, and the solvent was evaporated in vacuo. The crude product was purified by column chromatography (SiO_2_; *n*-hexane/EtOAc 12:1). The product was obtained as a yellow solid. Yield: 307 mg, 1.17 mmol, 94%. ^1^H NMR (500 MHz, CDCl_3_): δ 10.48 (s, 1H), 8.16 (d, *J* = 7.6 Hz, 1H), 7.89 (d, *J* = 7.8 Hz, 1H), 7.70–7.61 (m, 2H), 7.59–7.49 (m, 2H), 7.48–7.39 (m, 3H) ppm; ^13^C{^1^H} NMR (126 MHz, CDCl_3_): δ 184.6, 143.6, 141.2, 139.6, 132.1 (2), 129.7, 129.0 (2), 128.8, 127.9, 125.7, 125.2, 123.5, 122.1, 99.2, 80.7 ppm; HRMS (APCI) *m*/*z*: [M + H]^+^ calcd for C_17_H_11_OS^+^, 263.0525; found, 263.0522.

##### Synthesis of 2-(2,2-Dibromovinyl)-3-(phenylethynyl)benzo[*b*]thiophene (**4**)

This compound was synthesized from compound **3** according to **GP1**. The reaction was stirred for 0.5 h. The crude product was purified by column chromatography (SiO_2_; *n*-hexane), yielding a pale-yellow solid. Yield: 119 mg, 0.29 mmol, 78%. ^1^H NMR (500 MHz, CDCl_3_): δ 8.23 (s, 1H), 8.01 (dd, *J* = 5.2, 3.8 Hz, 1H), 7.80 (dd, *J* = 5.2, 3.7 Hz, 1H), 7.64 (dd, *J* = 6.5, 3.0 Hz, 2H), 7.48–7.38 (m, 5H) ppm; ^13^C{^1^H} NMR (126 MHz, CDCl_3_): δ 140.4, 138.6, 138.2, 131.8 (2), 131.2, 129.0, 128.7 (2), 126.5, 125.4, 123.5, 122.9, 122.3, 120.5, 98.3, 91.2, 82.5 ppm; HRMS (APCI) *m*/*z*: [M + H]^+^ calcd for C_18_H_11_SBr_2_^+^, 416.8942; found, 416.8957.

##### Synthesis of 1,2,3-Triphenylbenzo[*b*]pentaleno[1,2-*d*]thiophene (**I**)

The synthesis of this compound was attempted from compound **4** according to **GP2**. The desired product was not detected.

##### Synthesis of 3-(Phenylethynyl)thiophene-2-carbaldehyde (**6**)^46^

3-Bromothiophene-2-carbaldehyde (**5**) (400 mg, 2.09 mmol), phenylacetylene (256.6 mg, 276 μL, 2.51 mmol), Pd(PPh_3_)_2_Cl_2_ (44 mg, 62.8 μmol), and CuI (11.9 mg, 62.8 μmol) were stirred in THF (15 mL) at rt under an inert atmosphere (N_2_). After a few minutes, diisopropylamine (5 mL) was added to the reaction mixture. Following the addition, the reaction was stirred for 18 h at 60 °C in an aluminum heating block. The reaction was monitored by TLC (*n*-hexane/EtOAc 12:1). Upon completion, the mixture was diluted with EtOAc and washed twice with water and once with brine. The organic phase was dried over MgSO_4_. The crude product was purified by column chromatography [SiO_2_, *n*-hexane → *n*-hexane/EtOAc (12:1)]. The product was obtained as a yellow oil. Yield: 309 mg, 1.46 mmol, 69%. ^1^H NMR (500 MHz, CDCl_3_): δ 10.24 (d, *J* = 1.3 Hz, 1H), 7.69 (dd, *J* = 5.0, 1.3 Hz, 1H), 7.59–7.51 (m, 2H), 7.43–7.33 (m, 3H), 7.25 (d, *J* = 5.0 Hz, 1H) ppm; ^13^C{^1^H} NMR (126 MHz, CDCl_3_): δ 183.1, 143.7, 134.0, 131.9 (2), 131.7, 131.1, 129.4, 128.7 (2), 122.1, 96.2, 81.7 ppm; HRMS (APCI) *m*/*z*: [M + H]^+^ calcd for C_13_H_9_OS^+^, 213.0368; found, 213.0360.

##### Synthesis of 2-(2,2-Dibromovinyl)-3-(phenylethynyl)thiophene (**7**)

This product was prepared from compound **6** according to **GP1**. The reaction was stirred for 3 h. The product was obtained as a colorless oil. Yield: 469 mg, 0.839 mmol, 90%. ^1^H NMR (500 MHz, CDCl_3_): δ 8.06 (s, 1H), 7.57–7.52 (m, 2H), 7.40–7.33 (m, 4H), 7.13 (d, *J* = 5.2 Hz, 1H); ^13^C{^1^H} NMR (126 MHz, CDCl_3_): δ 140.1, 131.7 (2), 130.5, 129.3, 128.8, 128.6 (2), 126.4, 124.4, 122.9, 95.2, 88.8, 83.6 ppm; HRMS (APCI) *m*/*z*: [M + H]^+^ calcd for C_14_H_9_SBr_2_^+^, 366.8786; found, 366.8799.

##### Synthesis of 4,5,6-Triphenylpentaleno[2,1-*b*]thiophene (**II**)

The synthesis of this compound was attempted from compound **7** according to **GP2**. The desired product was not detected.

##### Synthesis of 4-Bromothiophene-3-carbaldehyde (**9**)^64^

3,4-Dibromothiophene (**8**) (3.00 g, 1.37 mL, 12.4 mmol) was stirred in diethyl ether (15 mL) under an inert atmosphere (N_2_) at −78 °C (acetone/dry ice) for 5 min, then *n*-BuLi (5.21 mL, 2.5 M in hexane) was added dropwise to the reaction. The mixture was stirred for 40 min at −78 °C, then DMF (1.36 g, 1.45 mL, 18.6 mmol) was added, and the reaction was stirred for another 2 h. The reaction was quenched with saturated NH_4_Cl solution and allowed to warm to rt. The mixture was extracted twice with diethyl ether, and the combined organic phase was dried over MgSO_4_. The solvent was evaporated under reduced pressure. The crude product was purified by column chromatography [SiO_2_, *n*-hexane → *n*-hexane/EtOAc (9:1)]. The product was obtained as a yellow oil. Yield: 1.94 g, 10.15 mmol, 82%. ^1^H NMR (500 MHz, CDCl_3_): δ 9.95 (s, 1H), 8.16 (d, *J* = 3.4 Hz, 1H), 7.36 (d, *J* = 3.4 Hz, 1H) ppm; ^13^C{^1^H} NMR (126 MHz, CDCl_3_): δ 184.8, 137.7, 134.7, 125.2, 111.5 ppm; HRMS (APCI) *m*/*z*: [M + H]^+^ calcd for C_5_H_4_OSBr^+^, 190.9160; found, 190.9154.

##### Synthesis of 4-(Phenylethynyl)thiophene-3-carbaldehyde (**10**)

4-Bromothiophene-3-carbaldehyde (**9**) (500 mg, 2.62 mmol), phenylacetylene (321 mg, 345 μL, 3.14 mmol), Pd(PPh_3_)_2_Cl_2_ (55 mg, 78.5 μmol), and CuI (15 mg, 78.5 μmol) were stirred in THF (12 mL) at rt under an inert atmosphere (N_2_). After a few minutes, diisopropylamine (3 mL) was added to the mixture. After the addition, the reaction was stirred overnight at 60 °C in an aluminum heating block. The reaction was monitored by TLC [*n*-hexane/EtOAc (12:1)]. Upon the completion of the reaction, the mixture was diluted with EtOAc and washed twice with water and once with brine. The organic phase was dried over MgSO_4_. After evaporation of the solvent under reduced pressure, the crude product was purified by column chromatography [SiO_2_, *n*-hexane → *n*-hexane/EtOAc (12:1)]. The product was obtained as a brown solid. Yield: 502 mg, 2.36 mmol, 90%. ^1^H NMR (500 MHz, CDCl_3_): δ 10.15 (s, 1H), 8.14 (d, *J* = 3.2 Hz, 1H), 7.58–7.53 (m, 3H), 7.39–7.34 (m, 3H) ppm; ^13^C{^1^H} NMR (126 MHz, CDCl_3_): δ 185.0, 140.7, 133.1, 131.8 (2), 130.3, 129.0, 128.6 (2), 123.4, 122.6, 93.1, 81.6 ppm; HRMS (APCI) *m*/*z*: [M + H]^+^ calcd for C_13_H_9_OS^+^, 213.0368; found, 213.0359.

##### Synthesis of 3-(2,2-Dibromovinyl)-4-(phenylethynyl)thiophene (**11**)

The product was prepared from compound **10** according to **GP1**. The reaction was stirred for 3 h. The product was obtained as a pale yellow solid. Yield: 796 mg, 2.16 mmol, 92%. ^1^H NMR (500 MHz, CDCl_3_): δ 8.06 (d, *J* = 3.0 Hz, 1H), 7.71 (s, 1H), 7.57–7.51 (m, 2H), 7.50 (d, *J* = 3.1 Hz, 1H), 7.40–7.34 (m, 3H) ppm; ^13^C{^1^H} NMR (126 MHz, CDCl_3_): δ 136.5, 131.8 (2), 130.3, 128.8, 128.6 (2), 128.1, 124.4, 123.6, 122.9, 92.9, 90.4, 82.9 ppm; HRMS (APCI) *m*/*z*: [M + H]^+^ calcd for C_14_H_9_SBr_2_^+^, 366.8786; found, 366.8799.

##### Synthesis of 4,5,6-Triphenylpentaleno[1,2-*c*]thiophene (**III**)

This product was prepared from compound **11** according to **GP2**. The product was obtained as a green solid. Yield: 9 mg, 23.3 μmol, 11%. ^1^H NMR (500 MHz, CD_2_Cl_2_): δ 7.25–7.17 (m, 9H), 7.12–7.08 (m, 4H), 7.01–6.97 (m, 2H), 6.69 (s, 2H), 6.53 (s, 1H) ppm; ^13^C{^1^H} NMR (126 MHz, CD_2_Cl_2_): δ 160.8, 153.8, 148.7, 139.1, 139.1, 136.0, 135.2, 135.2, 134.5, 132.0, 130.2 (2), 129.2 (2), 129.2 (2), 129.0, 128.7 (2), 128.5 (2), 128.5 (2), 128.3, 128.0, 127.3, 118.8, 115.3 ppm; HRMS (APCI) *m*/*z*: [M + H]^+^ calcd for C_28_H_19_S^+^, 387.1201; found, 387.1204.

##### Synthesis of 2-Ethynyl-3-(phenylethynyl)benzo[*b*]thiophene (**12**)^46^

This compound was synthesized from **3** according to **GP3**. The reaction was stirred for 1 h. The product was purified by column chromatography (SiO_2_; *n*-hexane) and obtained as a brownish yellow solid. Yield: 100 mg, 387.1 μmol, 53%. ^1^H NMR (500 MHz, CDCl_3_): δ 8.03–7.98 (m, 1H), 7.79–7.75 (m, 1H), 7.70–7.65 (m, 2H), 7.52–7.44 (m, 2H), 7.44–7.38 (m, 3H), 3.79 (s, 1H) ppm; ^13^C{^1^H} NMR (126 MHz, CDCl_3_): δ 138.6, 138.5, 132.0 (2), 128.8, 128.5 (2), 126.7, 125.4, 124.8, 124.2, 123.8, 123.0, 122.3, 96.6, 87.4, 82.3, 76.7 ppm; HRMS (APCI) *m*/*z*: [M + H]^+^ calcd for C_18_H_11_S^+^, 259.0575; found, 259.0568.

##### Synthesis of Benzo[*b*]benzo[4,5]pentaleno[1,2-*d*]thiophene [**V**(H)]

In a 20 mL vial, chloro[1,3-bis(2,6-diisopropylphenyl)imidazole-2-ylidene]gold(I) (29 mg, 46.6 μmol) and AgSbF_6_ (20 mg, 58.2 μmol) were stirred in 1,2-dichloroethane (1 mL) for 5 min under an inert atmosphere (N_2_) at rt. 2-Ethynyl-3-(phenylethynyl)benzo[*b*]thiophene (**12**) (277.0 mg, 1.07 μmol) in 1,2-dichloroethane (7 mL) was added to the stirred solution, and the mixture was stirred for 16 h under an inert atmosphere (N_2_) at 80 °C in an aluminum heating block. The reaction was monitored with TLC (*n*-hexane). After the completion of the reaction, the mixture was diluted with CH_2_Cl_2_ and filtered through a pad of Celite. The solvent was evaporated in vacuo. The crude product was purified by column chromatography (SiO_2_, *n*-hexane). The title compound was not obtained; instead, a yellow solid product was isolated, which is suggested to be structure **13**.^43^ Yield: 15 mg, 29.0 μmol, 30%. ^1^H NMR (500 MHz, CDCl_3_): δ 8.34 (d, *J* = 1.7 Hz, 1H), 8.23–8.19 (m, 1H), 8.06–8.03 (m, 1H), 7.96 (d, *J* = 1.7 Hz, 1H), 7.82–7.76 (m, 3H), 7.75–7.70 (m, 2H), 7.70–7.66 (m, 1H), 7.54–7.44 (m, 6H), 7.44–7.39 (m, 4H) ppm.

##### Synthesis of 2,3-Dibromobenzo[*b*]thiophene (**15**)^65^

Bromine (7.50 g, 2.35 mL, 46.93 mmol) in CHCl_3_ (10 mL) was added dropwise to a stirred solution of benzo[*b*]thiophene (**14**) (3.00 g, 22.36 mmol) in CHCl_3_ (50 mL) under an inert atmosphere (N_2_) at 0 °C. After the addition, the mixture was allowed to warm to rt, and it was stirred for an additional 18 h. The mixture was diluted with CHCl_3_ and washed once with water, twice with saturated NaHCO_3_, and once with brine. The organic phase was dried over MgSO_4_, and the solvent was evaporated in vacuo. The product was sufficiently pure to be used without any further purification. The product was obtained as a white solid. Yield: 6.48 g, 22.2 mmol, >95%. ^1^H NMR (500 MHz, CDCl_3_): δ 7.75 (d, *J* = 7.4 Hz, 1H), 7.72 (d, *J* = 8.0 Hz, 1H), 7.43 (td, *J* = 7.6, 1.2 Hz, 1H), 7.38 (td, *J* = 7.7, 1.3 Hz, 1H) ppm; ^13^C{^1^H} NMR (126 MHz, CDCl_3_): δ 139.1, 137.7, 125.8, 125.7, 123.5, 122.0, 114.4, 111.9; HRMS (APCI) *m*/*z*: [M]^+^ calcd for C_8_H_4_SBr_2_^+^, 289.8400; found, 289.8403.

##### Synthesis of 2,3-Bis(phenylethynyl)benzo[*b*]thiophene (**16**)^66^

2,3-Dibromobenzo[*b*]thiophene (**15**) (600 mg, 2.05 mmol), phenylacetylene (504 mg, 542 μL, 4.93 mmol), Pd(PPh_3_)_2_Cl_2_ (72 mg, 103 μmol), and CuI (19.6 mg, 103 μmol) were stirred in THF (10 mL) at rt under a N_2_ atmosphere. After a few minutes, diisopropylamine (2 mL) was added to the reaction mixture. After the addition, the reaction was stirred for 18 h at 60 °C in an aluminum heating block. The reaction was monitored by TLC (*n*-hexane). The reaction mixture was diluted with EtOAc and washed once with 1 M HCl and once with brine. The organic phase was dried over MgSO_4_. The solvent was evaporated under reduced pressure. The crude product was purified by column chromatography (SiO_2_, *n*-hexane) and obtained as a yellow solid. Yield: 599 mg, 1.79 mmol, 87%. ^1^H NMR (500 MHz, CDCl_3_): δ 8.03–7.98 (m, 1H), 7.78 (d, *J* = 7.8 Hz, 1H), 7.70–7.65 (m, 2H), 7.65–7.60 (m, 2H), 7.51–7.37 (m, 8H) ppm; ^13^C{^1^H} NMR (126 MHz, CDCl_3_): δ 138.8, 138.8, 131.9 (2), 131.8 (2), 129.1, 128.7, 128.6 (2), 128.6 (2), 126.4, 126.4, 125.3, 123.6, 123.3, 122.9, 122.8, 122.3, 99.8, 96.6, 83.0, 82.8 ppm; HRMS (APCI) *m*/*z*: [M + H]^+^ calcd for C_24_H_15_S^+^, 335.0888; found, 335.0894.

##### Synthesis of 11-Phenylbenzo[*b*]benzo[4,5]pentaleno[1,2-*d*]thiophene and 6-phenylbenzo[*b*]benzo[4,5] pentaleno[2,1-*d*]thiophene [**V**(Ph)]

In a 40 mL vial, chloro[1,3-bis(2,6-diisopropylphenyl)imidazole-2-ylidene]gold(I) (18.3 mg, 53.2 μmol) and AgSbF_6_ (33.1 mg, 53.2 μmol) were stirred in degassed dry 1,2-dichloroethane (2 mL) for 5 min under an inert atmosphere (N_2_). 2,3-Bis(phenylethynyl)benzo[*b*]thiophene (**16**) (356 mg, 1.06 mmol) in degassed dry 1,2-dichloroethane (8 mL) was added to the stirred solution, and the mixture was stirred for 6 h under an inert atmosphere (N_2_) at 65 °C in an aluminum heating block. The reaction was monitored with TLC (*n*-hexane). After the completion of the reaction, the mixture was diluted with CH_2_Cl_2_ and filtered through a pad of Celite. The solvent was evaporated in vacuo. The crude product was purified by column chromatography (SiO_2_, *n*-hexane). The products were obtained as an inseparable mixture of isomers and other impurities. HRMS (APCI) *m*/*z*: [M + H]^+^ calcd for C_24_H_15_S^+^, 335,0888; found, 335,0888.

##### Synthesis of 3-((4-Methoxyphenyl)ethynyl)benzo[*b*]thiophene-2-carbaldehyde (**17**)^67^

3-Bromobenzo[*b*]thiophene-2-carbaldehyde (**2**) (300 mg, 1.24 mmol), 1-ethynyl-4-methoxybenzene (197 mg, 1.5 mmol), Pd(PPh_3_)_2_Cl_2_ (26.4 mg, 37.6 μmol), and CuI (7 mg, 37.6 μmol) were stirred under an inert atmosphere (N_2_) in triethylamine (10 mL) at rt for 5 h. After the reaction was completed, the mixture was diluted with EtOAc and filtered through a pad of Celite. The solvent was evaporated in vacuo. The crude product was purified by column chromatography [SiO_2_, *n*-hexane → *n*-hexane/EtOAc (9:1)]. The product was obtained as a yellow solid. Yield: 265 mg, 0.88 mmol, 73%. ^1^H NMR (500 MHz, CDCl_3_): δ 10.44 (s, 1H), 8.16–8.10 (m, 1H), 7.87–7.82 (m, 1H), 7.58 (d, *J* = 8.8 Hz, 2H), 7.55–7.47 (m, 2H), 6.94 (d, *J* = 8.8 Hz, 2H), 3.86 (s, 3H) ppm; ^13^C{^1^H} NMR (126 MHz, CDCl_3_): δ 184.6, 160.8, 142.9, 141.2, 139.5, 133.7 (2), 128.9, 128.4, 125.6, 125.12, 123.4, 114.5 (2), 114.0, 99.6, 79.8, 55.5 ppm; HRMS (APCI) *m*/*z*: [M + H]^+^ calcd for C_18_H_13_O_2_S^+^, 293.0630; found, 293.0632.

##### Synthesis of 2-Ethynyl-3-((4-methoxyphenyl)ethynyl)benzo[*b*]thiophene (**18**)^46^

This compound was synthesized from **17** according to **GP3**. The reaction was stirred for 3 h. The product was purified by column chromatography [SiO_2_, *n*-hexane → *n*-hexane/EtOAc (12:1)] and obtained as a brownish yellow solid. Yield: 154 mg, 0,534 mmol, 59%. ^1^H NMR (500 MHz, CDCl_3_): δ 8.00–7.95 (m, 1H), 7.77–7.72 (m, 1H), 7.59 (d, *J* = 8.8 Hz, 2H), 7.49–7.42 (m, 2H), 6.92 (d, *J* = 8.8 Hz, 2H), 3.85 (s, 3H), 3.76 (s, 1H) ppm; ^13^C{^1^H} NMR (126 MHz, CDCl_3_): δ 160.2, 138.6, 138.6, 133.5 (2), 126.6, 125.3, 124.6, 124.0, 123.8, 122.3, 115.1, 114.2 (2), 96.8, 87.2, 81.2, 76.9, 55.5 ppm; HRMS (APCI) *m*/*z*: [M + H]^+^ calcd for C_19_H_13_OS^+^, 289.0681; found, 289.0677.

##### Synthesis of 3-((4-Methoxyphenyl)ethynyl)-2-(phenylethynyl)benzo[*b*]thiophene (**19**)

2-Ethynyl-3-((4-methoxyphenyl)ethynyl)benzo[*b*]thiophene (**18**) (140 mg, 486 μmol), iodobenzene (149 mg, 81 μL, 728 μmol), Pd(PPh_3_)_2_Cl_2_ (10.2 mg, 14.6 μmol), and CuI (2.8 mg, 14.6 μmol) were stirred in THF (7 mL) at rt under an inert atmosphere (N_2_). After a few minutes, diisopropylamine (0.5 mL) was added to the reaction mixture. After the addition, the reaction was stirred overnight (16 h) at rt. The reaction was monitored by TLC (*n*-hexane/EtOAc 12:1). The reaction mixture was diluted with EtOAc and washed twice with water and once with brine. The organic phase was dried over MgSO_4_. The solvent was evaporated in vacuo. The crude product was purified by column chromatography [SiO_2_, *n*-hexane → *n*-hexane/EtOAc (12:1)]. The product was obtained as a yellow oil. Yield: 147 mg, 0.403 mmol, 83%. ^1^H NMR (500 MHz, CDCl_3_): δ 8.02–7.97 (m, 1H), 7.79–7.75 (m, 1H), 7.65–7.58 (m, 4H), 7.50–7.41 (m, 2H), 7.41–7.36 (m, 3H), 6.94 (d, *J* = 8.7 Hz, 2H), 3.86 (s, 3H) ppm; ^13^C{^1^H} NMR (126 MHz, CDCl_3_): δ 160.1, 138.9, 138.8, 133.4 (2), 131.8 (2), 129.0, 128.6 (2), 126.3, 125.6, 125.2, 123.6, 123.3, 122.8, 122.3, 115.4, 114.3 (2), 99.5, 96.8, 83.0, 81.7, 55.5 ppm; HRMS (APCI) *m*/*z*: [M + H]^+^ calcd for C_25_H_17_OS^+^, 365.0994; found, 365.0996.

##### Synthesis of 11-(4-Methoxyphenyl)benzo[*b*]benzo[4,5]pentaleno[1,2-*d*]thiophene [**V**(PMP)]

In a 4 mL vial, chloro[1,3-bis(2,6-diisopropylphenyl)imidazol-2-ylidene]gold(I) (6.7 mg, 10.8 μmol) and AgSbF_6_ (3.7 mg, 10.8 μmol) were stirred in degassed dry 1,2-dichloroethane (1 mL) under an inert atmosphere (N_2_) at rt. After 5 min, 3-((4-methoxyphenyl)ethynyl)-2-(phenylethynyl)benzo[*b*]thiophene (**19**) (78.7 mg, 216 μmol) in degassed dry 1,2-dichloroethane (1 mL) was added to the stirred solution, and the mixture was stirred for 3 h under an inert atmosphere (N_2_) at 65 °C in an aluminum heating block. The reaction was monitored with TLC (*n*-hexane). After the completion of the reaction, the mixture was diluted with CH_2_Cl_2_ and filtered through a pad of Celite. The solvent was evaporated in vacuo. The crude product was purified by column chromatography [SiO_2_, *n*-hexane → *n*-hexane/EtOAc (12:1)]. The product is a deep green solid. Yield: 7.2 mg, 19.8 μmol, 9%. ^1^H NMR (500 MHz, CD_2_Cl_2_): δ 7.60–7.52 (m, 3H), 7.09–7.05 (m, 2H), 7.05–7.00 (m, 3H), 6.81–6.73 (m, 2H), 6.72–6.65 (m, 2H), 6.10 (s, 1H), 3.91 (s, 3H) ppm; ^13^C{^1^H} NMR (126 MHz, CD_2_Cl_2_): δ 161.6, 152.8, 151.2, 150.7, 144.7, 143.9, 141.7, 137.7, 133.7, 132.8, 130.8 (2), 129.1, 128.3, 126.7, 125.5, 124.3, 123.8, 123.4, 122.7, 121.6, 119.5, 114.7 (2), 56.0 ppm; HRMS (APCI) *m*/*z*: [M + H]^+^ calcd for C_25_H_17_OS^+^, 365.0994; found, 365.1001.

##### Synthesis of 3,4-Bis(phenylethynyl)thiophene (**20**)

3,4-Dibromothiophene (**8**) (750 mg, 343 μL 3.10 mmol), phenylacetylene (950 mg, 1.02 mL, 9.30 mmol), Pd(PPh_3_)_2_Cl_2_ (65 mg, 93 μmol), and CuI (18 mg, 93 μmol) were stirred in THF (12 mL) at rt under an inert atmosphere (N_2_). After a few minutes, diisopropylamine (3 mL) was added to the reaction mixture. After the addition, the reaction was stirred overnight at 60 °C in an aluminum heating block. The reaction was monitored by TLC (*n*-hexane). Upon completion, the mixture was diluted with EtOAc and washed twice with water and once with brine. The organic phase was dried over MgSO_4_. The crude product was purified by column chromatography (SiO_2_, *n*-hexane). The product was obtained as a yellow solid. Yield: 378 mg, 1.33 mmol, 43%. ^1^H NMR (500 MHz, CDCl_3_): δ 7.59–7.52 (m, 4H), 7.50 (s, 2H), 7.36–7.32 (m, 6H) ppm; ^13^C{^1^H} NMR (126 MHz, CDCl_3_): δ 131.8 (4), 129.2 (2), 128.5 (4), 128.1 (2), 125.2 (2), 123.3 (2), 91.9 (2), 83.5 (2) ppm; HRMS (APCI) *m*/*z*: [M + H]^+^ calcd for C_20_H_13_S^+^, 285.0732; found, 285.0726.

##### Synthesis of 4-Phenylbenzo[4,5]pentaleno[1,2-*c*]thiophene (**VI**)

In a 4 mL vial, chloro[1,3-bis(2,6-diisopropylphenyl)imidazol-2-ylidene]gold(I) (6.2 mg, 10.0 μmol) and AgSbF_6_ (3.4 mg, 10.0 μmol) were stirred in degassed dry 1,2-dichloroethane (1 mL) for 5 min under an inert atmosphere (N_2_). After that, 3,4-bis(phenylethynyl)thiophene (**20**) (57.0 mg, 200 μmol) in degassed dry 1,2-dichloroethane (1 mL) was added to the stirred solution, and the mixture was stirred for 3 h under an inert atmosphere (N_2_) at 65 °C in an aluminum heating block. The reaction was monitored by TLC (SiO_2_, hexane). After the completion of the reaction, the mixture was diluted with CH_2_Cl_2_ and filtered through a pad of Celite. The solvent was evaporated in vacuo. The crude product was purified by column chromatography (SiO_2_, *n*-hexane). The product was obtained as an orange solid. Yield: 25 mg, 87.9 μmol, 44%. mp 134.4–136.2 °C; ^1^H NMR (500 MHz, CD_2_Cl_2_): δ 7.73 (d, *J* = 7.2 Hz, 2H), 7.52 (t, *J* = 7.6 Hz, 2H), 7.43 (t, *J* = 7.4 Hz, 1H), 7.36–7.27 (m, 2H), 7.11–7.02 (m, 2H), 6.96 (d, *J* = 1.8 Hz, 1H), 6.72 (d, *J* = 1.8 Hz, 1H), 6.62 (s, 1H) ppm; ^13^C{^1^H} NMR (126 MHz, CD_2_Cl_2_): δ 155.3, 154.5, 148.6, 138.4, 137.5, 136.5, 135.0, 134.8, 129.4 (2), 129.2, 128.8, 128.5 (2), 127.3, 122.9, 122.7, 121.6, 117.0, 116.2 ppm; HRMS (APCI) *m*/*z*: [M + H]^+^ calcd for C_20_H_13_S^+^, 285.0732; found, 285.0726.

## Supporting Information Available

Crystallographic data of 4-Phenylbenzo[4,5]pentaleno[1,2-*c*]thiophene (**VI**) (CIF)
Additional UV–vis, X-ray crystallography, CV data, ^1^H and ^13^C NMR spectra, ACID plots, NICS-*XY* scans, MCI and FLU data, further aromaticity analysis, and computational details for all new compounds (PDF)

## Supplementary Material

ao1c05618_si_001.cif
