# Supplementary material for: Unsymmetrical Thienopentalenes: Synthesis, Optoelectronic Properties, and (Anti)aromaticity Analysis
Source: ACS Omega. 2022 Mar 3;7(10):8336–49. doi: 10.1021/acsomega.1c05618 (PMC8928497; doi:10.1021/acsomega.1c05618)
Supplement: Supplementary file 2 — ao1c05618_si_002.pdf [file ao1c05618_si_002.pdf]

Supporting Information

Unsymmetrical thienopentalenes: synthesis, opto-electronic properties and (anti)aromaticity analysis

Tamás Gazdag,[a,b] Péter J. Mayer,[a,c] Péter Pál Kalapos,[a] Tamás Holczbauer,[d] Ouissam El Bakouri[e] and Gábor London[a]*

[a] MTA TTK Lendület Functional Organic Materials Research Group, Institute of Organic Chemistry, Research Centre for Natural Sciences, 1117 Budapest, Magyar tudósok krt. 2., Hungary

[b] Hevesy György PhD School of Chemistry, Eötvös Loránd University, Pázmány Péter sétány 1/a, Budapest 1117, Hungary

[c] Institute of Chemistry, University of Szeged, 6720 Szeged, Rerrich tér 1., Hungary

[d] Centre for Structural Science and Institute of Organic Chemistry, Research Centre for Natural Sciences, 1117 Budapest, Magyar tudósok körútja 2., Hungary

[e] Institut de Química Computacional i Catàlisi (IQCC) and Departament de Química, Universitat de Girona, C/ Maria Aurèlia Capmany 6, 17003 Girona, Catalonia, Spain.

Table of contents

S1. X-Ray crystallography 3

S1.1 Comparison of solid-state packings of **VI** and **DBP** 7

S2. UV-Vis spectroscopy data 9

S3. Electrochemistry 10

S3.1 CV measurements on compound **III** 11

S3.2 CV measurements on compound **V**(PMP) 13

S3.3 CV measurements on compound **VI** 15

S3.4 Summary of CV measurements 18

S4. Computational studies 19

S4.1 General methods 19

S4.2 Aromaticity analyses 23

S4.2.1 ACID analyses 23

S4.2.2 NICS-*XY* scans 26

S4.2.3 Electronic aromaticity indices 27

S4.3 Cartesian coordinates and absolute electronic energies 31

S5. NMR spectra of compounds and intermediates 36

References 58

S1. X-Ray crystallography

Crystal data of compound **VI***:* C20 H12 S, *Fwt.*: 284.36, red, block, size: 0.280 x 0.250 x 0.220 mm, monoclinic, space group *P* 21/c, a = 15.5106(7)Å, b = 4.9274(2)Å, c = 18.1757(9)Å, α = 90°, β = 99.752(7)°, γ = 90°, V = 1369.04(11)Å^3, T = 138(2)K, Z= 4, F(000) = 592, D_x = 1.380 Mg/m^3, μ 0.225mm^{-1}.

A crystal of **VI** was mounted on a fiber. Cell parameters were determined by least-squares using 35483 (3.07 ≤ θ ≤ 27.515°) reflections.

Intensity data were collected on a Rigaku R-axis Rapid Spider II diffractometer[1] (monochromator; Mo-*K*α radiation, λ = 0.71075Å) at 138(2) K in the range 3.197 ≤ θ ≤ 25.347.

A total of 36556 reflections were collected of which 2486 were unique [R(int) = 0.0558, $R(\sigma)$ = 0.0211]; intensities of 2308 reflections were greater than 2σ(I). Completeness to θ = 0.998.

A numerical absorption correction[2] was applied to the data (the minimum and maximum transmission factors were 0.968289 and 0.991885).

The structure was solved by direct methods[3] (and subsequent difference syntheses).

Anisotropic full-matrix least-squares refinement on F^2 for all non-hydrogen atoms yielded R_1 = 0.0580 and wR^2 = 0.1243 for 1332 [I>2σ(I)] and R_1 = 0.0639 and wR^2 = 0.1272 for all (2486) intensity data, (number of parameters = 226, goodness-of-fit = 1.168, the maximum and mean shift/esd is 0.000 and 0.000).

The maximum and minimum residual electron density in the final difference map was 0.69 and -0.25e.Å^{-3}.

The weighting scheme applied was w = 1/[σd^2($F_o{}^2$)+(0.04251.7212P)^2+1.7212P] where P = ($F_o{}^2$+2$F_c{}^2$)/3.

Hydrogen atomic positions were calculated from assumed geometries. Hydrogen atoms were included in structure factor calculations but they were not refined. The isotropic displacement parameters of the hydrogen atoms were approximated from the U(eq) value of the atom they were bonded to. The structure is presented using the program Mercury.[4]

CCDC 2090075 contains the supplementary crystallographic data for this paper. These data can be obtained free of charge from The Cambridge Crystallographic Data Centre via www.ccdc.cam.ac.uk/structures.

Table S1. Summary of crystallographic data, data collections, structure determination and refinement for **VI**.

CCDC Number	2090075
Empirical formula	$C_{20}H_{12}S$
Formula weight	284.36
Temperature	138(2)
Radiation and wavelength	Mo-Kα, λ =0.71075Å
Crystal system	monoclinic
Space group	$P2_1/c$
Unit cell dimensions	*a* =15.5106(7)Å
	b =4.9274(2)Å
	c =18.1757(9)Å
	α =90°
	β =99.752(7)°
	γ =90°
Volume	1369.04(11)$Å^3$
Z, Z'	4, 1
Density (calculated)	1.380 Mg/m^3
Absorption coefficient, μ	0.225 mm^{-1}
F(000)	592
Crystal colour	red
Crystal description	block
Crystal size	0.28 x 0.25 x 0.22 mm
Absorption correction	numerical
Max. and min. transmission	0.968289 and 0.991885
θ−range for data collection	3.197 ≤ θ ≤ 25.347°
Index ranges	-18 ≤ *h* ≤ 18;
	-5≤ *k* ≤5;
	-21 ≤ *l* ≤ 21
Reflections collected	36556
Completeness to 2θ	0.998
Independent reflections	2486 [*R*(int) =0.0558]
Reflections *I*>2σ(*I*)	2308
Data / restraints / parameters	2486 /0 /226

Goodness-of-fit on F^2	1.168
Final R indices [I>2σ(I)]	R_1 =0.0580,
	wR^2 =0.1243
R indices (all data)	R_1 =0.0639,
	wR^2 =0.1272
Max. and mean shift/esd	0.000;0.000
Largest diff. peak and hole	0.69 and -0.25 e.Å^{-3}

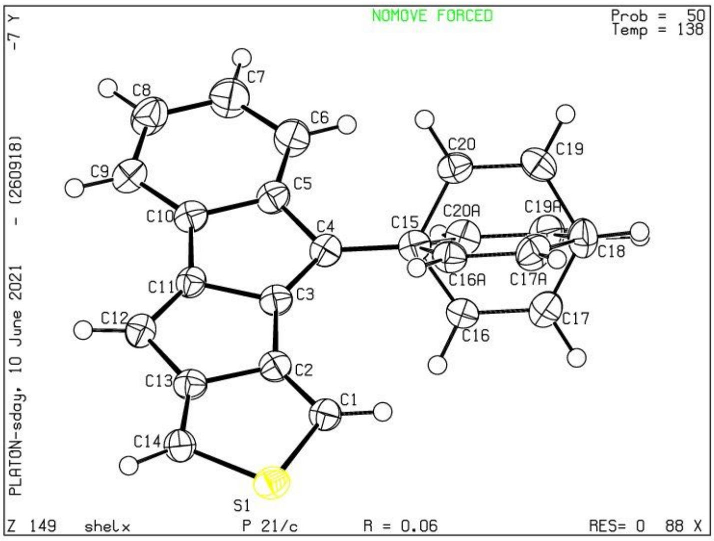

Figure S1. ORTEP style representation of **VI** is drawn at the 50% probability level.

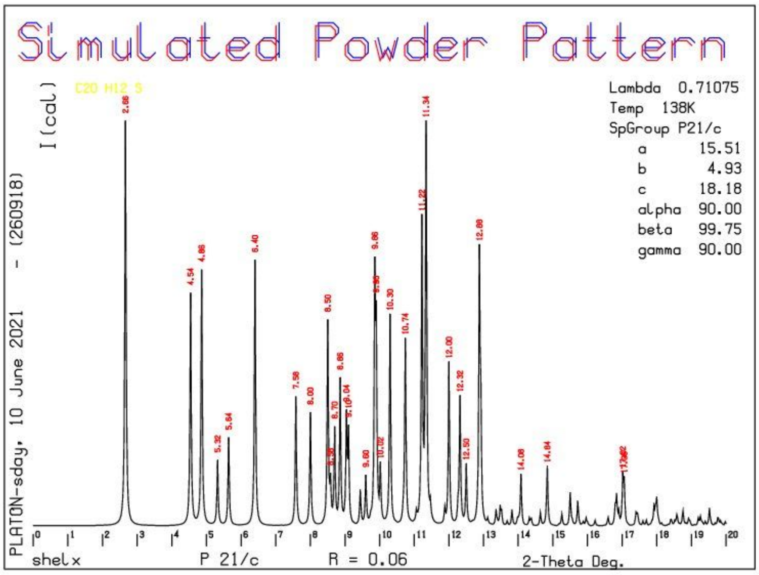

Figure S2. Simulated powder diffractogram of **VI**.

Table S2. View of crystallographic unit cells from different axis.

a axis	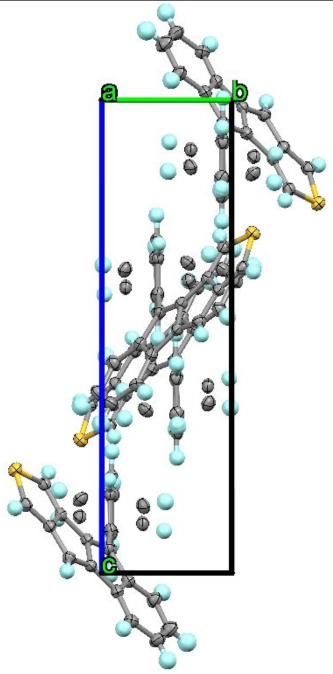
b axis	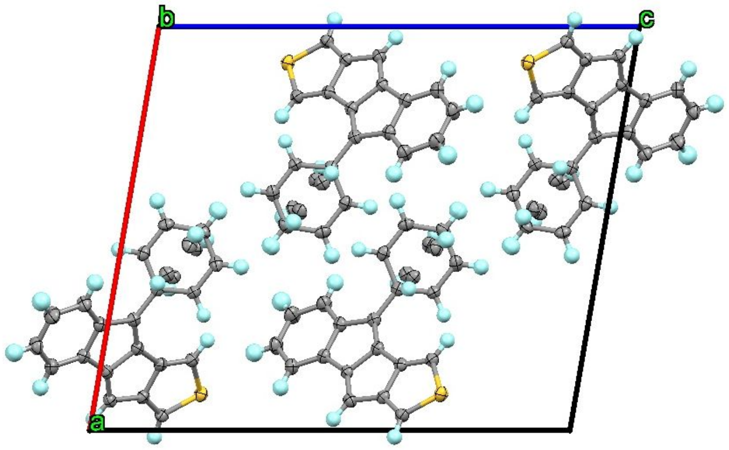
c axis	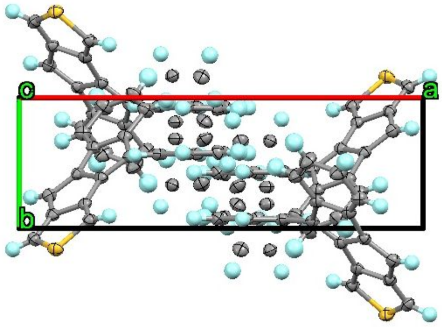

S1.1 Comparison of solid-state packings of VI and DBP

In the crystal of compound **VI**, one molecule with a disordered phenyl substituent was found in the asymmetric unit in the space group $P2_1/c$ (Figure S3).

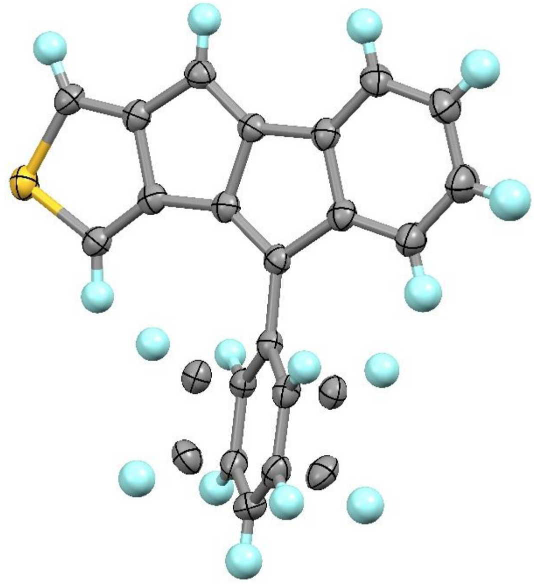

Figure S3. The asymmetric unit of the crystal of compound **VI**.

For **DBP**,[5] four molecules with disordered phenyl groups were found in the asymmetric unit in the space group *P*-1 (Figure S4).

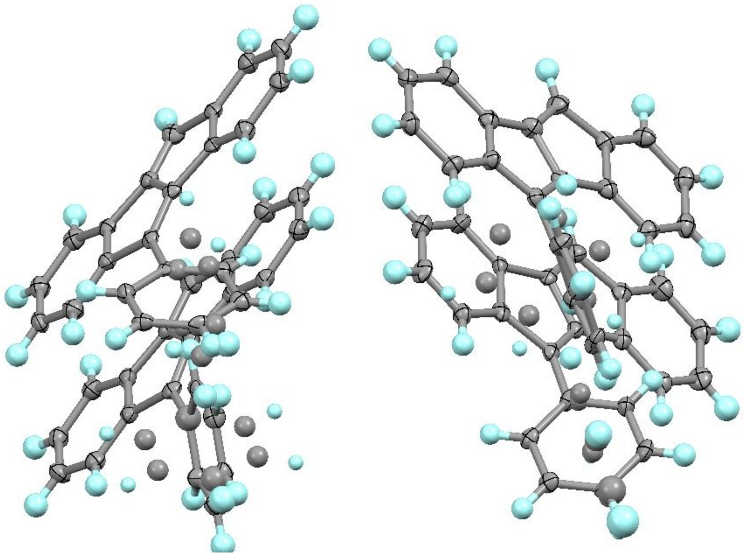

Figure S4. The asymmetric unit of the crystal of **DBP**.

DBP has similar supramolecular interactions within the crystal as compound **VI** despite the number of molecules in the asymmetric unit. The disordered phenyl groups within the molecules are present in two positions that are perpendicular to each other (83(1)^0 and 85(5)^0 in **VI** and **DBP**, respectively, relative to the plane of conjugated cores).
The conjugated cores of both molecules are arranged parallel due to π-stacking with a slight displacement along their longer axis (Figure S5).

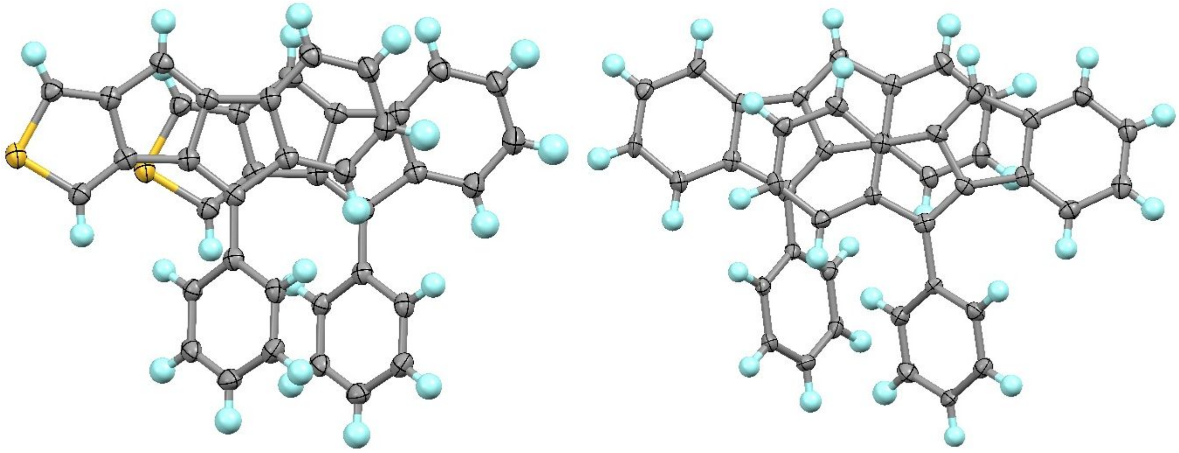

Figure S5. π-Stacked dimers of **VI** and **DBP**. One disordered position is omitted for clarity.

Besides the parallel π-stacking, "T-shaped" π-π interactions are present within the crystal lattices, leading to a herringbone arrangement in both cases (Figure S6).

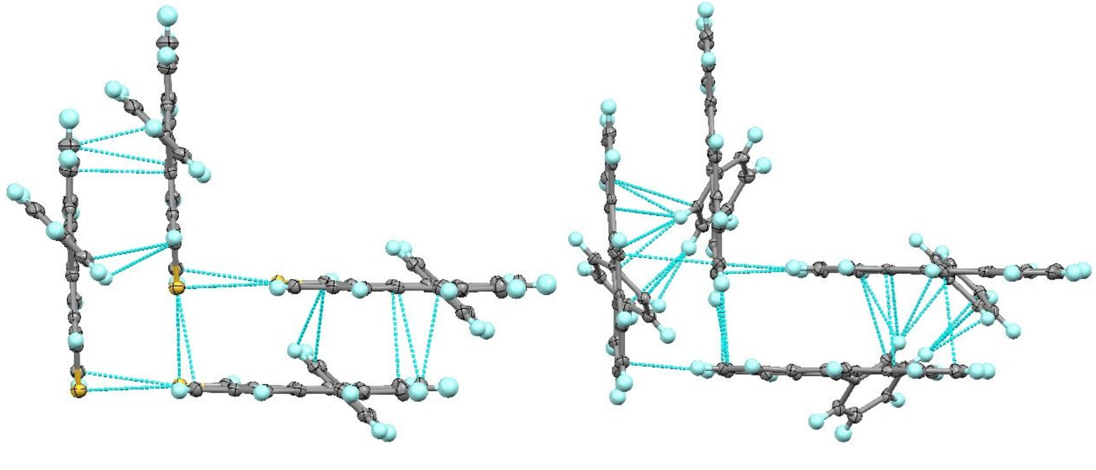

Figure S6. Herringbone arrangement in the crystal lattices of **VI** (left) and **DBP** (right). One disordered position is omitted for clarity.

S2. UV-Vis spectroscopy data

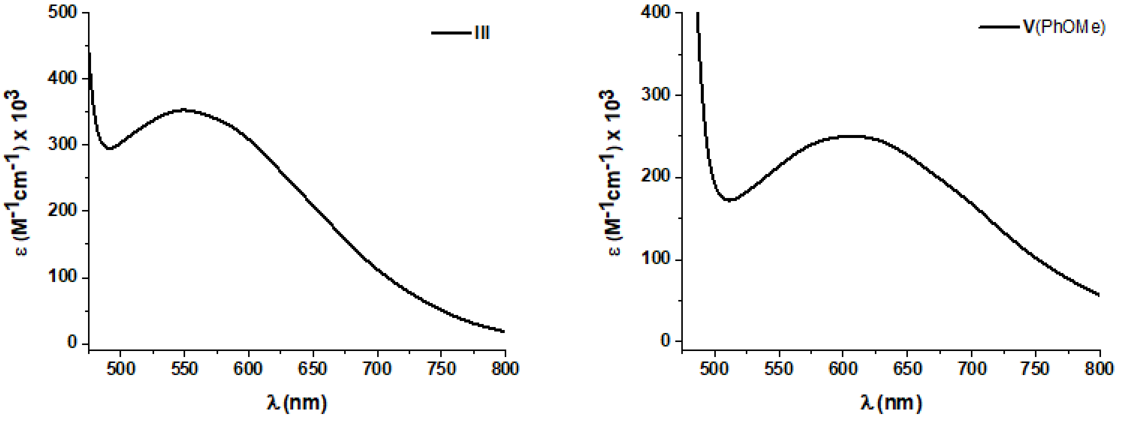

Figure S7. UV-Vis spectra in the 500-800 nm region of more concentrated samples (~ 10^{-3} M) of compounds **III** and **V**(PMP). Spectra were recorded in $CHCl_3$.

S3. Electrochemistry

Cyclic voltammetry was performed using a Zahner M6 potentiostat.

The solution and the electrodes were housed in a 4 mL screw cap vial. The following electrodes were used for all measurements (Figure S8): Pt counter-electrode (CE), Pt wire working-electrode (WE), Ag/AgCl reference electrode.

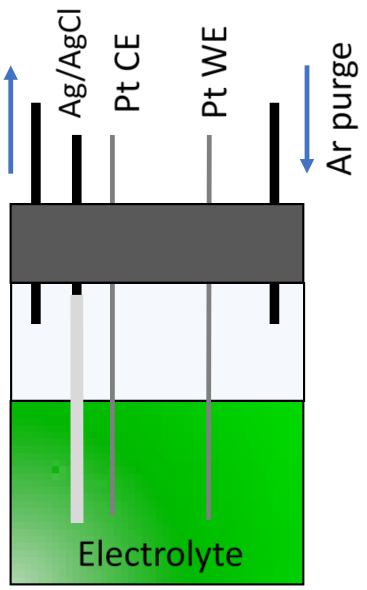

Figure S8. Schematic representation of the electrochemical cell.

General considerations for sample preparation: A solution containing 0.1 M Bu_4NPF_6 as the electrolyte, 0.001 M ferrocene as an internal standard, and the electroactive species in 0.001 M concentration was prepared. Spectroscopic grade dichloromethane (CH_2Cl_2) was used as a solvent in all cases. Oxygen was removed from the system by purging the solution with Ar gas (saturated with DCM vapor) for 3-5 minutes. The headspace above the solution was gently purged with Ar gas (saturated with DCM vapor) during data acquisition.

General considerations for data acquisition: A CV was recorded at 100, 500, 1000 and 2000 mV/s for every sample. The effect of ferrocene on the samples was evaluated by recording CVs in the absence of the reference compound.

S3.1 CV measurements on compound III

Compound **III** shows an oxidation at $E_{p,a}$=1.07 V (vs. Ag/AgCl) and an irreversible reduction at $E_{p,c}$=-1.26 V. Cyclic voltammograms recorded at different sweep-rates revealed the oxidation at $E_{p,a}$=1.07 V to be quasi-reversible ($E_{1/2}$=1.02 V), while the reduction was irreversible. At sweep-rates higher than 100 mV/s a new irreversible oxidation was visible at $E_{p,a}$=-0.40 V (Figure S9).

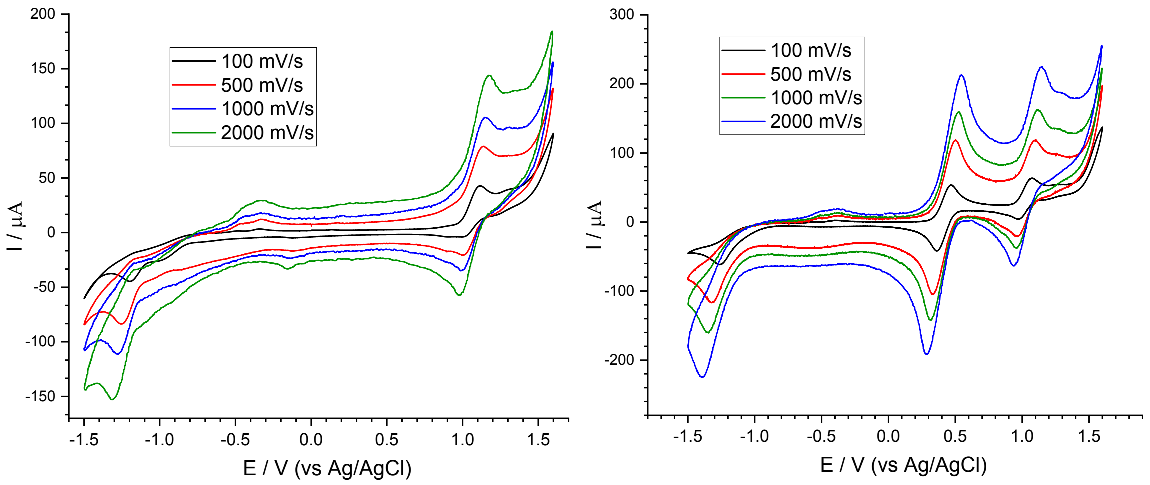

Figure S9. Cyclic voltammogram of compound **III** in CH_2Cl_2 with different sweep rates in the absence (left) and in the presence (right) of ferrocene. (10-point average smoothing was used for voltammograms recorded faster than 500 mV/s.)

The HOMO-LUMO gap was determined from onset potentials observed on the 100 mV/s sweep rate voltammogram. The potential values were acquired by linear extrapolation of the background current and the linear segment of the respective oxidations/reductions (Figure S10). Electrochemical data is summarized in Table S3.

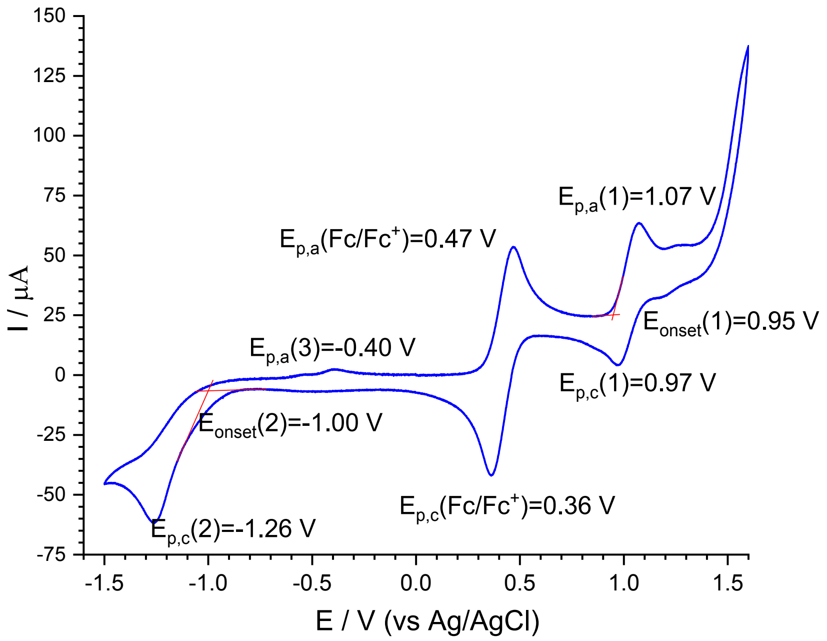

Figure S10. CV of compound **III** at 100 mV/s. Red lines indicate the extrapolation that was used for onset potential determination.

Table S3. Electrochemical data for compound III.

Potential	vs. Ag/AgCl (V)	vs. Fc/Fc+ (V)
$E_{1/2}$(Fc/Fc$^+$)	0.42	0.00
1st Oxidation ($E_{1/2}$(1))	1.02	0.60
1st Reduction ($E_{p,c}$(2))	-1.26	-1.68
HOMO (E_{onset}(1))	0.95	0.53
LUMO (E_{onset}(2))	-1.00	-1.42
HOMO-LUMO gap	1.95	1.95

S3.2 CV measurements on compound **V**(PMP)

Compound **V**(PMP) has shown a reversible oxidation at $E_{1/2}$=0.91 V (vs Ag/AgCl) and a reversible reduction at $E_{1/2}$=-1.18 V (Figure S11).

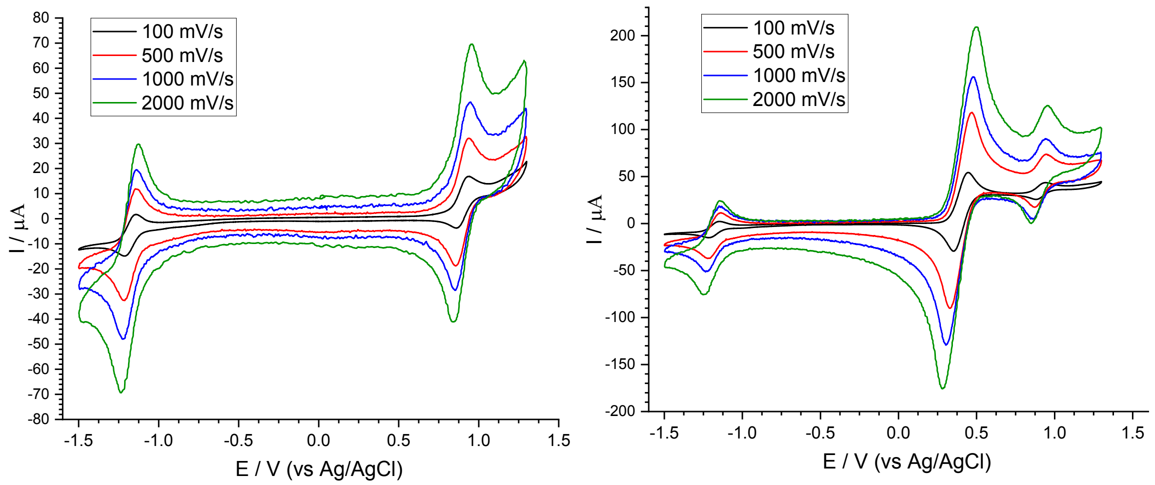

Figure S11. Cyclic voltammogram of **V**(PMP) with different sweep rates in the presence and in the absence of ferrocene. (10-point average smoothing was used for data recorded above 500 mV/s).

The HOMO-LUMO gap was determined from onset potentials observed on the 100 mV/s sweep rate voltammogram. The onset potentials were acquired by linear extrapolation of the background current and the linear segment of the respective oxidations/reductions (Figure S12). Electrochemical data is summarized in Table S4.

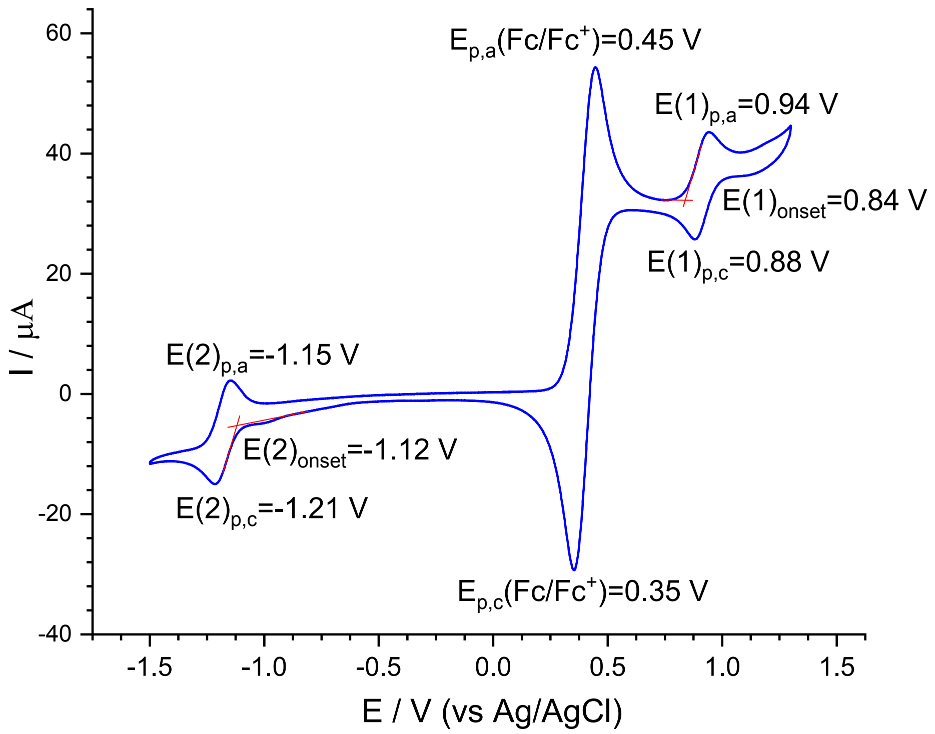

Figure S12. Cyclic voltammogram of **V**(PMP) at 100 mV/s.

Table S4. Electrochemical data for **V**(PMP).

Potential	vs. Ag/AgCl (V)	vs. Fc/Fc+ (V)
$E_{1/2}$(Fc/Fc$^+$)	0.40	0.00
1st Oxidation ($E_{1/2}$(1))	0.91	0.51
1st Reduction ($E_{1/2}$(2))	-1.18	-1.58
HOMO (E_{onset}(1))	0.84	0.44
LUMO (E_{onset}(2))	-1.12	-1.52
HOMO-LUMO gap	1.96	1.96

S3.3 CV measurements on compound VI

Compound **VI** has shown an irreversible oxidation and an irreversible reduction. Changing the sweep rate did not result in observable cathodic current for the oxidation, nor anodic current for the reduction, indicating the irreversibility of the processes (Figure S13). Interestingly, an irreversible reduction was observed for sweep rates higher than 100 mV/s at $E_{p,c}$=-0.05 V (vs. Ag/AgCl).

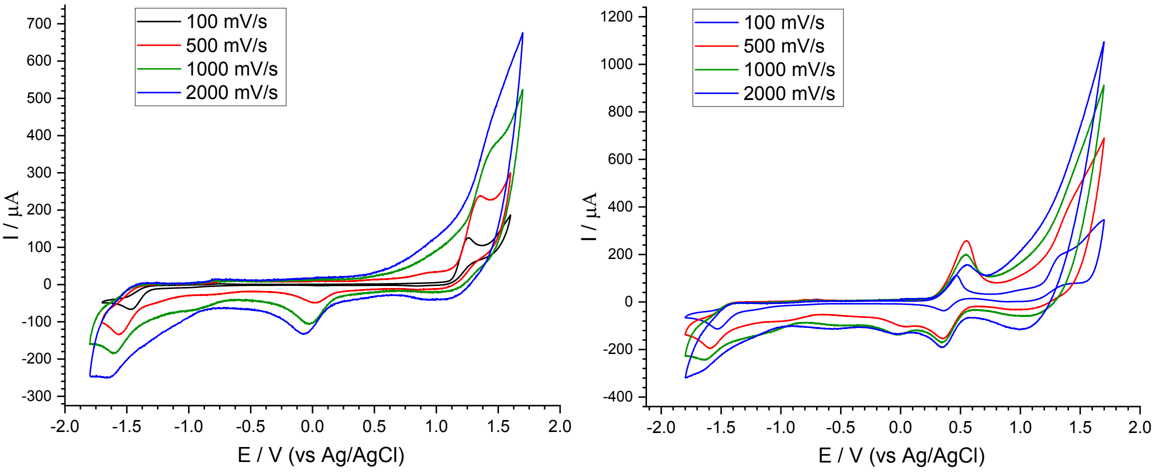

Figure S13. Cyclic voltammograms of **VI** at different sweep rates, in the presence and in the absence of ferrocene.

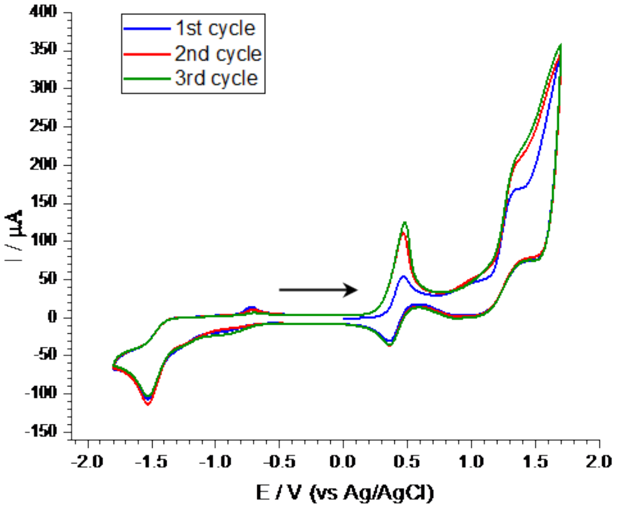

Figure S14. All three cycles of CV performed at 100 mV/s.

The sample containing ferrocene displayed slightly unusual electrochemical behavior. The first cycle scanned from 0 V towards positive potentials showed a well-defined ferrocene/ferrocenium couple and an irreversible oxidation. Surprisingly, the 2nd and 3rd cycle

displayed an unusually high anodic current for the oxidation of ferrocene (Figure S14). Also, the presence of the reference compound caused the appearance of small electrochemical signals with low currents, around the first oxidation (1.0 V) and reduction (-1.0 V). A voltammogram recorded in the presence and absence of ferrocene is shown on Figure S15.

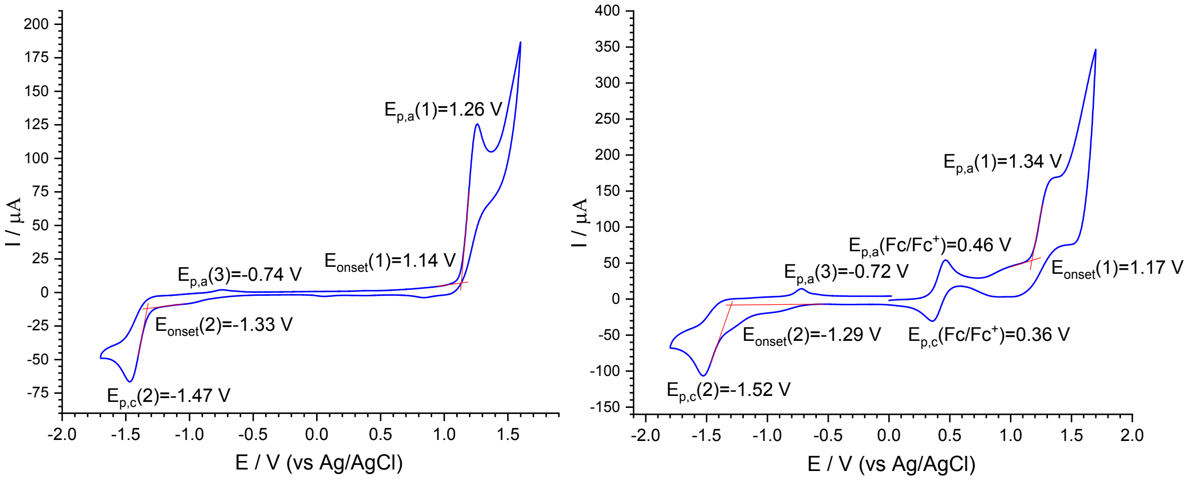

Figure S15. Cyclic voltammogram in the presence (left) and in the absence (right) of ferrocene recorded at 100 mV/s.

Even though some differences can be identified between the voltammograms in the presence and in the absence of ferrocene, the onset potentials for the first oxidation and reduction lie very close to each other. This allows us to extract HOMO-LUMO gap from these voltammograms, similarly to **III**.

An interesting observation was that after the experiments a red precipitate was found on the surface of the working electrode (Figure S16). It is possible that the formation of this layer caused the unusual electrochemical behavior.

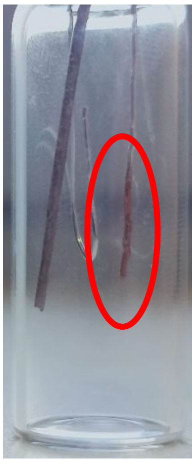

Figure S16. A red precipitate was observed on the surface of the working electrode after data acquisition. (Picture was taken by on of the co-authors (K.P.P.).)

Electrochemical data extracted from the voltammograms is summarized in Table S5.

Table S5. Electrochemical data for **VI**.

Potential	vs. Ag/AgCl (V)	vs. Fc/Fc+ (V)
$E_{1/2}$(Fc/Fc$^+$)	0.41	0.00
1st Oxidation ($E_{p,a}$(1))	1.34	0.93
1st Reduction ($E_{p,c}$(2))	-1.52	-1.93
HOMO (E_{onset}(1))	1.17	0.64
LUMO (E_{onset}(2))	-1.29	-1.70
HOMO-LUMO gap	2.46	2.46

S3.4 Summary of CV measurements

Compounds **III** and **V**(PMP) has shown usual electrochemical behavior. Their HOMO-LUMO gap could be estimated by determining the onset potentials for the first oxidation/reduction observed on a cyclic voltammogram recorded at 100 mV/s. The oxidation of **III** was quasi-reversible, while the reduction was irreversible. Both the oxidation and reduction of **V**(PMP) was highly reversible.

Compound **VI** showed unsettling electrochemical behavior. Both oxidations and reductions were found to be irreversible. The addition of ferrocene as a reference compound to the solution triggered the appearance of new, artifact signals. Also, the non-symmetrical Fc/Fc+ signal in some voltammograms is surprising. The unusual observations might be answered by the formation of a red precipitate on the electrode surface. Despite these, the HOMO-LUMO gap could be extracted from the onset potentials. HOMO-LUMO data extracted from the voltammograms is summarized in Table S6.

Table S6. Summary of HOMO-LUMO gaps.

Compound	Δ HOMO-LUMO (V)
**III**	1.95
**VI**	2.46
**V**(PMP)	1.96

S4. Computational studies

S4.1 General methods

All geometry optimizations were made with the Gaussian 09[6] package using the B3LYP[7] hybrid functional and the 6-311+G(d,p)[8] basis set. Analytical hessians were computed to confirm that the structures are minima. The conjugated cores of all considered molecules and the complete structures of the prepared molecules (**III**, **V**(PMP), **VI**) were studied. The conjugated cores are abbreviated as follows: **P*b*T** (pentaleno[2,1-*b*]thiophene), **P*c*T** (pentaleno[1,2-*c*]thiophene), **P*b*BT** (pentaleno[2,1-*b*]benzothiophene), **BP*b*T** (benzo[4,5]pentaleno[2,1-*b*]thiophene), **BP*c*T** (benzo[4,5]pentaleno[1,2-*c*]thiophene), **BP*b*BT** (benzo[4,5]pentaleno[2,1-*b*]benzothiophene). For the prepared compounds TD-DFT calculations were performed to obtain the HOMO-LUMO transition energies (with the following commands: td=(nstates=100) cam-b3lyp/6-311+g(d,p) scrf=(solvent=chloroform) scf=xqc). Aromaticity has been assessed by means of magnetic indices computed at the B3LYP/6-311+G(d,p) level. As magnetic indicators, the anisotropy of the induced current density (ACID)[9,10] plots and NICS-*XY* scans[11-14] were used. ACID plots give a qualitative picture of the ring-current nature so that clockwise and anticlockwise ring-currents indicate aromatic and antiaromatic character, respectively. NICS-*XY* scans, which take into account only π-contributions by employing the σ-model, provide quantitative information about diatropicity (negative values) and paratropicity (positive values) of the different circuits.

As electronic indices of aromaticity, we employed the multicenter index (MCI)[15] and the fluctuation aromaticity index (FLU).[16] MCI is obtained from I_{ring} values as follows:

$$MCI(\mathcal{A}) = \frac{1}{2N}\sum_{P(\mathcal{A})} I_{ring}(\mathcal{A})$$

where $P(\mathcal{A})$ stands for a permutation operator which interchanges the atomic labels A_1, A_2 ... A_N to generate up to the N! permutations of the elements in the string $\mathcal{A}$, and the I_{ring} index is defined as:

$$I_{ring}(\mathcal{A}) = \sum_{i_1,i_2,\ldots i_N} n_{i1}\ldots n_{iN} S_{i_1 i_2}(A_1) S_{i_2 i_3}(A_2) \ldots S_{i_N i_1}(A_N)$$

where $S_{ij}(A)$ is the overlap of natural orbitals i and j in the atom A defined within the QTAIM scheme,[17] and n_i are their occupancies. FLU is computed using delocalization indices, δ(A,B), with the expression:

$$FLU(\mathcal{A}) = \frac{1}{N}\sum_{i=1}^{N}\left[\left(\frac{V(A_i)}{V(A_{i-1})}\right)^{\alpha}\left(\frac{\delta(A_i,A_{i-1}) - \delta_{ref}(A_i,A_{i-1})}{\delta_{ref}(A_i,A_{i-1})}\right)\right]^2 \quad (1)$$

where $A_0 \equiv A_N$ and the string $\mathcal{A} = \{A_1,A_2,\ldots,A_N\}$ contains the ordered elements according to the connectivity of the N atoms in a ring or in a chosen circuit. V(A) is defined as:

$$V(A_i) = \sum_{A_j \neq A_i} \delta(A_i,A_j), \quad (2)$$

and α is a simple function to make sure that the first term is always greater or equal to 1. The delocalization indices of Eq. 1 were calculated using the overlaps between occupied molecular orbitals in the atomic basins generated by AIMAll program.[18] In FLU calculations, the δ_{ref} (C,C) and $\delta_{ref}(C,S)$ reference values of 1.389 e and 1.270 e used for C–C and C–S bonds corresponds to the $\delta(C,C)$ of benzene and $\delta(C,S)$ of thiophen, respectively, computed at the B3LYP/6-311+G(d,p) level of theory. FLU and MCI were obtained with the ESI program.[19]

Table S7. Orbital assignments, transition energies and oscillator strengths (*f*) for **III**, **V**(PMP) and **VI**.

Molecule	Transition	ΔE (eV)	λ (nm)	*f*
**III**	HOMO→LUMO	2.2528	550.36	0.0118
	HOMO-1→LUMO	3.2087	386.40	0.4060
	HOMO→LUMO+1	4.1004	302.37	0.3162
**V** PMP)	HOMO→LUMO	1.9920	622.40	0.0213
	HOMO-1→LUMO	3.0969	400.35	0.4671
	HOMO-2→LUMO	3.9529	313.65	0.1973
	HOMO-3→LUMO	4.0191	308.49	0.0877
**VI**	HOMO→LUMO	2.8123	440.86	0.0151
	HOMO-1→LUMO	3.2520	381.25	0.4523
	HOMO→LUMO+1	4.4229	280.32	0.3012

Table S8. Depiction of calculated HOMOs and LUMOs of **III**, **V**(PMP) and **VI** (at the 0.02 a.u. isosurface).

Compound	HOMO-1	HOMO	LUMO	LUMO+1
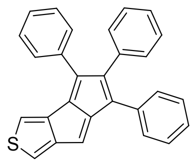 **III**	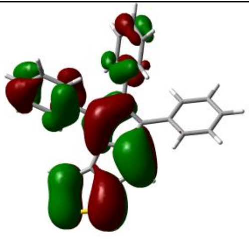	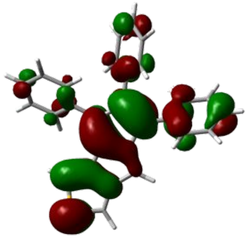	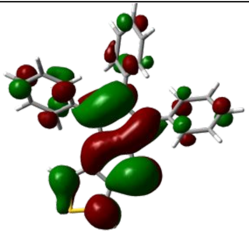	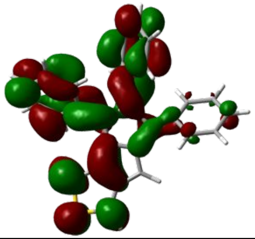
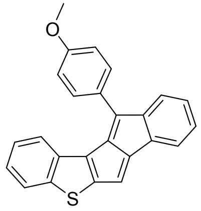 **V**(PhOMe)	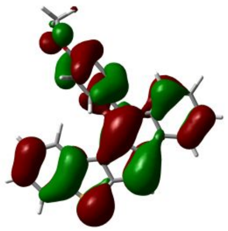	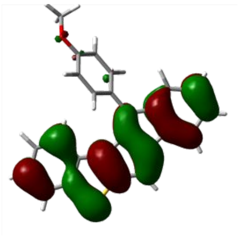	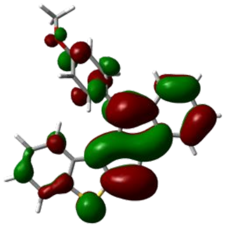	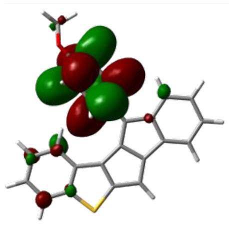
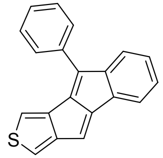 **VI**	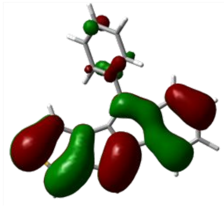	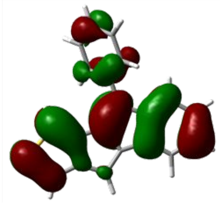	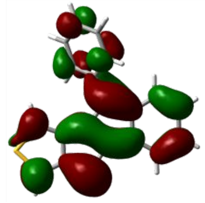	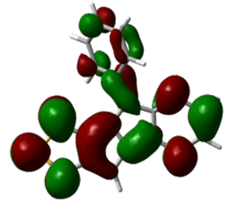

S4.2 Aromaticity analyses

S4.2.1 ACID analyses

Table S9. ACID plots of the conjugated cores **P*b*T**, **P*c*T**, **P*b*BT**, **BP*b*T**, **BP*c*T** and **BP*b*BT** in their ground state. In the plots, the directions of the induced ring currents are shown by small arrows, where the red dots indicate the heads, while the green lines indicate the shafts of the arrows.

Entry	Structure	ACID plot	Brief analysis
1	**P*b*T**	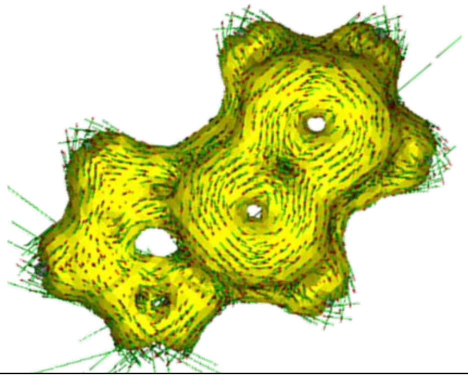	An antiaromatic (AA) counterclockwise ring-current can be observed throughout the pentalene ring system. The thiophene ring shows non-aromatic (NA) character.
2	**P*c*T**	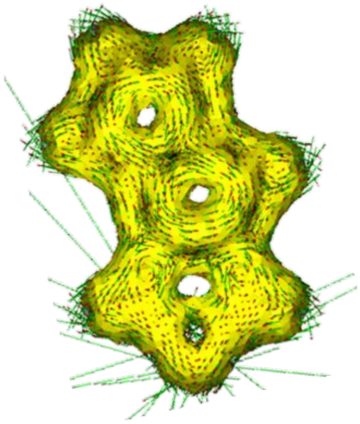	A weak antiaromatic (WAA) counterclockwise ring-current can be observed throughout the pentalene ring system, while the thiophene ring shows NA character.
3	**P*b*BT**	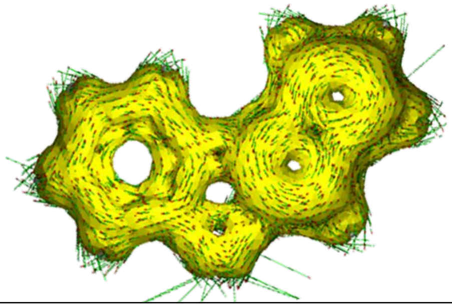	AA ring-current can be observed in the pentalene ring system; the thiophene ring is NA; the benzene ring of the benzothiophene shows aromatic (A) character.

4	**BP*b*T**	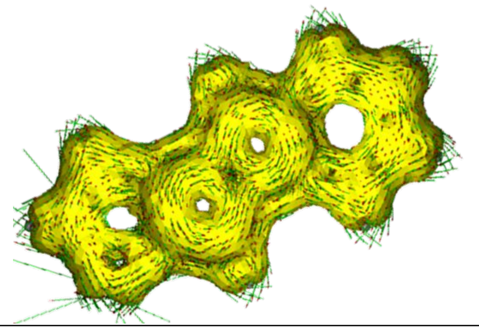	Weak aromatic (WA) ring-current can be observed in the benzene ring of the benzopentalene moiety; AA ring-current in the pentalene ring system; NA character in the thiophene
5	**BP*c*T**	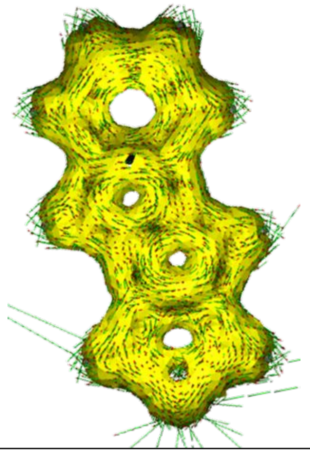	Aromatic ring-current in the benzene ring of the benzopentalene moiety; WAA ring-current in the pentalene ring system; NA character in the thiophene
6	**BP*b*BT**	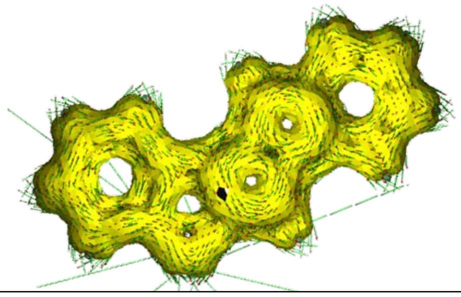	NA character in the benzene ring of the benzopentalene moiety; AA ring-current in the pentalene ring system; NA character in the thiophene; A ring-current in the benzene of the benzothiophene

The pentalene moiety (Table S9) maintains higher antiaromaticity when it is fused to (benzo)thiophene through the "*b*" (2,3) bond of thiophene. Fusion through the "*c*" (3,4) bond of thiophene leads to alleviated antiaromaticity. These are in agreement with the bond order dependent antiaromaticity reported by Haley and co-workers.[20] The benzene ring that is fused to the pentalene subunit is weakening the antiaromatic character of the pentalene moiety.

Table S10. ACID plots of the prepared compounds **III**, **VI** and **V**(PhOMe) in their ground state. In the plots, the directions of the induced ring currents are shown by small arrows, where the red dots indicate the heads, while the green lines indicate the shafts of the arrows.

Entry	Molecule	ACID plot	Brief analysis
1	**III**	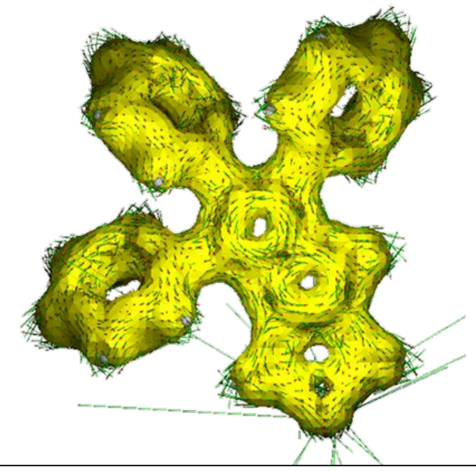	A weak antiaromatic (WAA) counterclockwise ring-current can be observed throughout the pentalene ring system, while the thiophene ring shows NA character.
2	**VI**	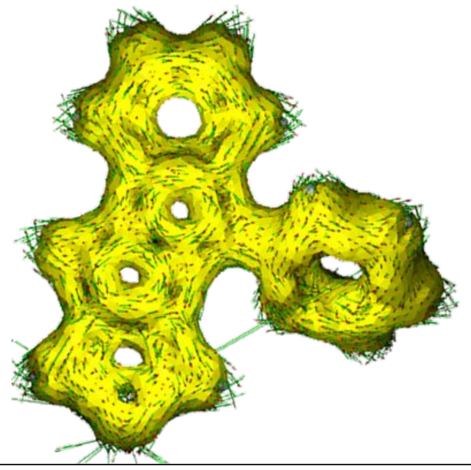	WA ring-current in the benzene ring of the benzopentalene moiety; WAA ring-current in the pentalene ring; NA character in the thiophene
3	**V**(PMP)	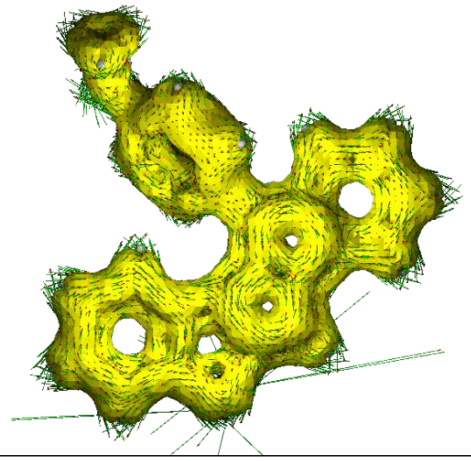	NA character in the benzene ring of the benzopentalene moiety; AA ring-current in the pentalene ring; WA character in the thiophene; A ring-current in the benzene of the benzothiophene

S4.2.2 NICS-*XY* scans

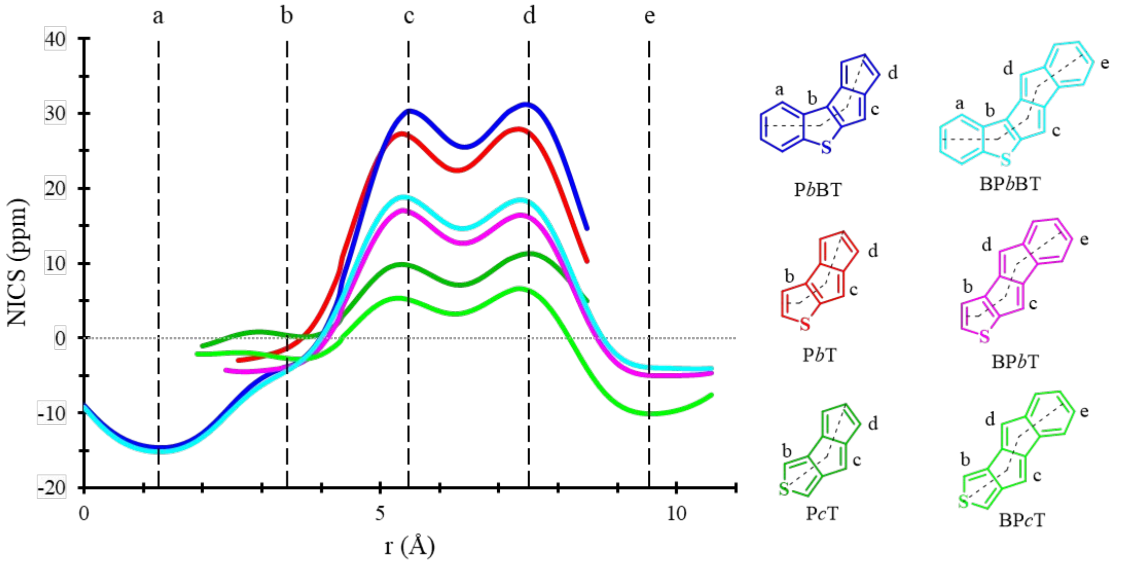

Figure S17. Comparison of NICS-*XY* scans of conjugated cores **P*b*T**, **P*c*T**, **P*b*BT**, **BP*b*T**, **BP*c*T** and **BP*b*BT** in their ground state.

The NICS-*XY* scans – in agreement with the ACID plots – reveal the alleviation of antiaromaticity upon fusing an additional benzene ring to a given monoareno pentalene structure (**P*b*BT** vs **BP*b*BT**; **P*b*T** vs. **BP*b*T**; **P*c*T** vs. **BP*c*T**).

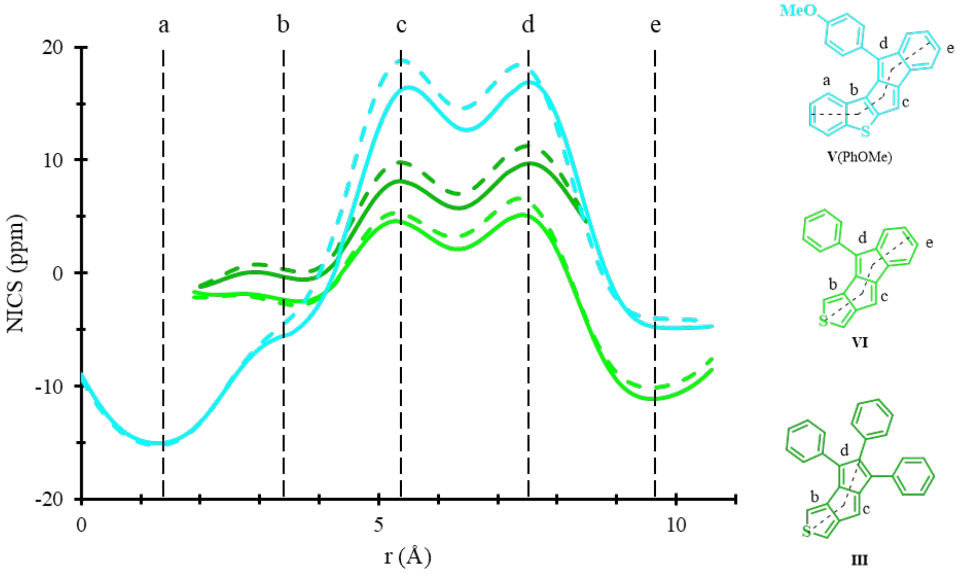

Figure S18. Comparison of the NICS-*XY* scans of the prepared molecules (**III**, **VI** and **V**(PMP)) to each other and to their conjugated cores (dashed line is the scan of the appropriate core).

The prepared compounds and the corresponding conjugated cores have a similar NICS-*XY* scan profiles. The ‘a’ ring of **V**(PMP) has local aromaticity while the pentalene moiety of the molecule is antiaromatic. In compounds **III** and **VI** where the thiophene fusion is through its *c* (3,4) bond, the pentalene units shows low antiaromaticity/approaching non-aromaticity.
The NICS-*XY* scans and the ACID plots are in agreement regarding the (anti)aromatic characters of the studied molecules.

S4.2.3 Electronic aromaticity indices

To assess (anti)aromaticity by means of the electronic structure we used the multicenter index (MCI) and the aromatic fluctuation index (FLU) which can provide information about the electron delocalization (as well as localization) in a ring. The lower the MCI and the higher the FLU, the more antiaromatic the ring/circuit is. Thus, to complement the results of magnetic aromaticity calculations (NICS, ACID), we also analyzed the synthesized molecules as well as the non-isolated ones by means of MCI and FLU (Table S11), along with the delocalization indices (DIs) (which FLU depends on) and bond lengths.
FLU data reveals that, among all compounds, **I** has the most antiaromatic pentalene subunit (FLU=0.0384 e) followed by II (FLU=0.0366 e), which is in agreement with MCI and NICS-*XY* scans. Yet, the overall antiaromaticity trend based on FLU, i.e. **I**>**II**>**VI**>**III**>**IV**≈**V**, is not the same as found by MCI and NICS-*XY* scans (for NICS-*XY* scans see Figure S18). Two main unexpected behaviors are observed: (i) the pentalene subunit of the non-isolated compound **IV** is less antiaromatic (FLU = 0.0326 e) than its isolated isomer (**VI**, FLU = 0.0360 e) but similar to compound **V** (FLU = 0.0310 e), and (ii) the antiaromaticity of the pentalene unit in the isolated compounds increase in the opposite order compared to what is found by NICS-*XY* and MCI, i.e. **VI**>**III**>**V**(PMP).

Table S11. MCI (normal text) and FLU (*italics*) values (in electrons) of the synthesized and non-isolated molecules. Values for pentalene is given for comparison.

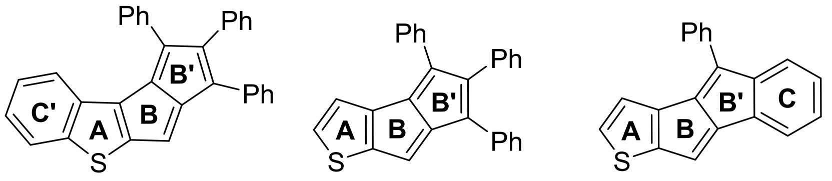

I **II** **IV**

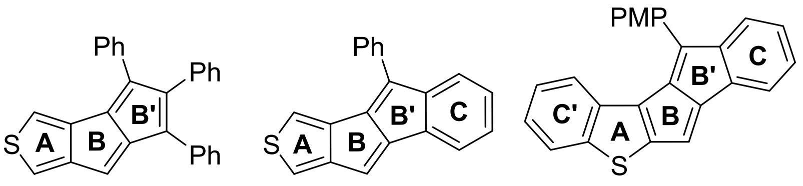

III **VI** **V**(PMP)

	A	***BB'***	***C***	***C'***
**Pentalene**	-	-0.0050, *0.0450*	-	-
**I**	0.0141, *0.0104*	-0.0026, *0.0384*	-	0.0471, *0.0046*
**II**	0.0331, *0.0032*	-0.0024, *0.0366*	-	-
**III**	0.0217, *0.0145*	0.0008, *0.0355*	-	-
**IV**	0.0316, *0.0044*	0.0004, *0.0326*	0.0529, *0.0036*	-
**V**	0.0128, *0.0112*	0.0004, *0.0310*	0.0533, *0.0034*	0.0485, *0.0040*
**VI**	0.0231, *0.0132*	0.0007, *0.0360*	0.0531, *0.0028*	-

The difference between the results provided by MCI (and NICS-*XY* scans) and FLU on the antiaromaticity of **BB'** could be explained based on bond lengths and DIs (which FLU depends on) of each bond in the molecules (Figure S19).

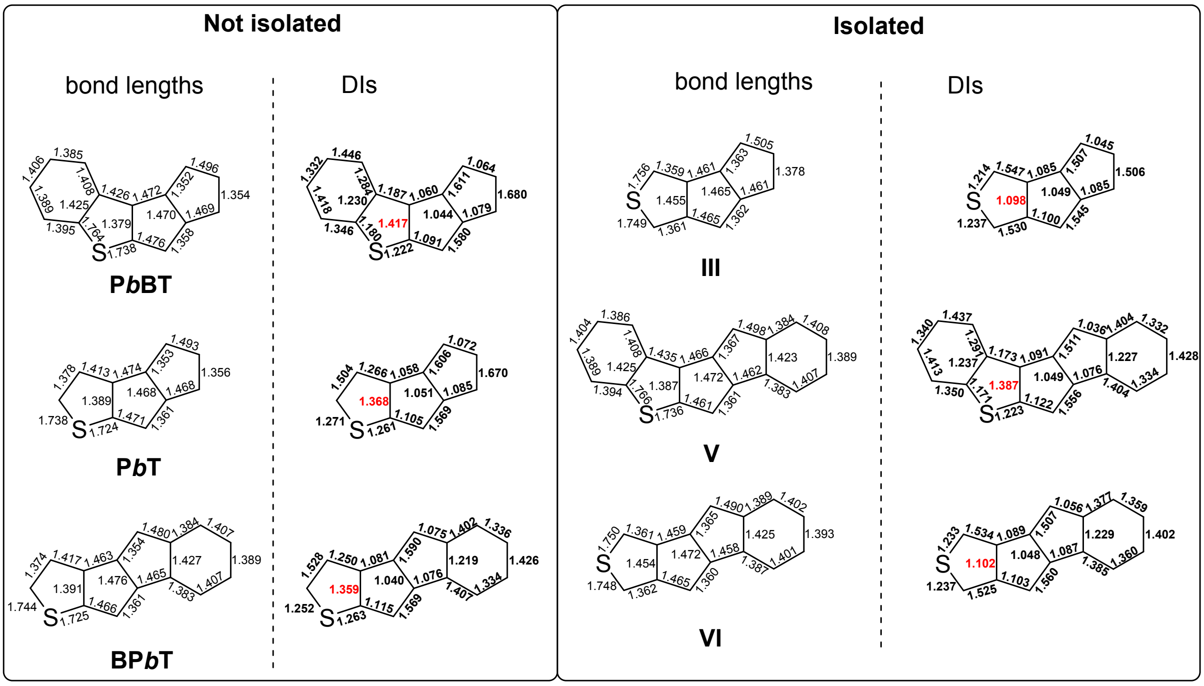

Figure S19. Bond lengths and delocalization indices (DIs) of the non-isolated and isolated molecules. In case of the non-isolated molecules calculations performed on the conjugated cores, while for the isolated molecules substituents are omitted for clarity. Bond lengths and DIs are in Å and electrons, respectively.

NICS-*XY* scans tell that the pentalene subunit in **IV** is more antiaromatic than in **VI** while it is the opposite according to FLU. The difference between the two compounds is the connectivity of the thiophene ring and, in terms of DIs, the bond that suffers the major change is the one between the thiophene and pentalene units. The DI of that bond decrease from 1.359 e (in **IV**) to 1.102 e (in **VI**) and, considering that the DI reference value for an aromatic C-C bond (used in the calculation of FLU) is 1.389 e, the value of 1.102 e is far from it. This explains why, according to FLU, the pentalene unit in **VI** is more antiaromatic than in **IV**, i.e. $FLU_{VI} > FLU_{IV}$. This is also reflected in the bond lengths of the shared bond, as it is more of a single bond character in **VI** compared to **IV**.

While NICS-*XY* scans show that the antiaromaticity of the pentalene unit increases in the order of **VI**<**III**<<**V**(PMP), FLU tells otherwise. From the calculated DI values, one can identify that the bond that suffers the largest deviation in terms of DI is again the one that is shared between the pentalene and the thiophene ring. DIs for this bond change from 1.102 e (for **VI**) to 1.387 e (for **V**). The value of 1.387 e is very close to the DI reference value (1.389 e). This explains why FLU predicts an increase of delocalization of the pentalene unit going from **VI** (FLU = 0.0360 e) to **V**(PMP) (FLU = 0.0310 e). This is the main reason why FLU is decreased in the

case of **V**(PMP) and indicates lower antiaromaticity for its pentalene unit compared to compound **VI**. Additionally, the presence of a stronger pentalene type conjugation in **V**(PMP) compared to **VI** is also reflected in the extent of bond-length alternation across the thiophene/pentalene shared bond and the adjacent bonds within the pentalene framework.

S4.3 Cartesian coordinates and absolute electronic energies

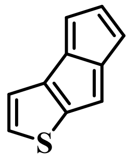

Pentaleno[2,1-*b*]thiophene (**P*b*T**)

E = -782.899251790 Hartree

	X	Y	Z
C	-0.40101200	0.66697800	-0.00001400
C	-0.83098000	-0.65403700	-0.00001300
C	0.31076800	-1.58124900	-0.00000200
C	1.43563400	-0.81536500	-0.00000200
C	1.07143000	0.60627800	-0.00001000
C	2.90103800	-0.89461200	0.00000200
C	3.36310500	0.38020300	0.00001600
C	2.21317400	1.33232500	-0.00000700
C	-1.46828900	1.59306800	0.00000000
C	-2.69365400	0.96346500	0.00001100
S	-2.55139700	-0.76882400	0.00000400
H	0.24123600	-2.66090800	0.00000000
H	3.49398000	-1.79763300	0.00000500
H	4.40049800	0.68533500	0.00002900
H	2.31650200	2.40968600	-0.00000700
H	-1.36232300	2.66971800	0.00000100
H	-3.67482300	1.41266700	0.00001700

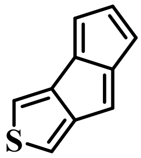

Pentaleno[1,2-*c*]thiophene (**P*c*T**)

E = -782.910675935 Hartree

	X	Y	Z
C	0.40726400	-0.51513900	0.00000100
C	0.73557500	0.90093300	0.00000100
C	-0.51188400	1.67568600	0.00000000
C	-1.55009100	0.79699400	-0.00000100
C	-1.05154100	-0.59438300	0.00000000
C	-3.00473000	0.73934700	-0.00000100
C	-3.34045300	-0.58084400	0.00000000
C	-2.12407800	-1.42374100	0.00000000
C	1.50799200	-1.30821300	0.00000000
S	2.98157800	-0.34894900	-0.00000100
C	2.07278000	1.14662900	0.00000000
H	-0.56836900	2.75671900	0.00000100
H	-3.68271000	1.57997500	-0.00000200
H	-4.34760600	-0.97654000	0.00000100
H	-2.13083800	-2.50519800	0.00000100
H	1.58096500	-2.38507800	-0.00000100
H	2.59830500	2.08969800	0.00000000

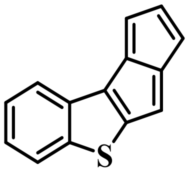

Benzo[*b*]pentaleno[1,2-*d*]thiophene (**P*b*BT**)

E = -936.583779048 Hartree

	X	Y	Z
C	-0.32792200	-0.09121100	-0.00002300
C	-0.45503200	1.28145500	-0.00001900
C	-1.87408200	1.68593100	-0.00000300
C	-2.60213800	0.53936500	0.00000700
C	-1.70076500	-0.62236300	-0.00000500
C	-3.98014700	0.02916300	0.00001500
C	-3.89733300	-1.32231800	0.00001100
C	-2.46101200	-1.74064500	-0.00000600
C	1.02870800	-0.53082200	-0.00000900
C	1.92302200	0.57922800	0.00000200
S	1.06667500	2.12180200	0.00000100
C	3.30724100	0.40491400	0.00001100
C	3.81268500	-0.88873000	0.00001200
C	2.94790300	-1.99776300	0.00000300
C	1.57357100	-1.82940400	-0.00000700
H	-2.23401600	2.70597600	-0.00000200
H	-4.88245600	0.62298800	0.00002700
H	-4.72755800	-2.01511300	0.00001700
H	-2.13128500	-2.77130700	-0.00001700
H	3.97490200	1.25877500	0.00001800

	X	Y	Z
H	4.88537700	-1.04315400	0.00001900
H	3.36577700	-2.99804200	0.00000400
H	0.91426400	-2.68974100	-0.00001500

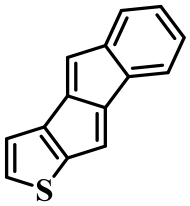

Benzo[4,5]pentaleno[2,1-*b*]thiophene (**BP*b*T**)

E = -936.601163216 Hartree

	X	Y	Z
C	-1.66869300	0.70410900	-0.00002400
C	-1.96569800	-0.65512100	-0.00002300
C	-0.74677800	-1.47027100	-0.00001300
C	0.29848400	-0.59890500	-0.00001500
C	-0.20767700	0.78754100	-0.00002000
C	1.76186000	-0.52796000	-0.00000400
C	2.09109500	0.86052900	-0.00000100
C	0.83817300	1.64794300	-0.00001000
C	-2.82412200	1.52406400	0.00000300
C	-3.97881200	0.78023200	0.00002400
S	-3.66742800	-0.93596000	0.00001300
C	3.41299600	1.27003000	0.00000900
C	4.42666900	0.29449200	0.00001500
C	4.10636200	-1.05743400	0.00001100
C	2.76434600	-1.48080500	0.00000100
H	-0.71471000	-2.55090400	-0.00001100
H	0.80283200	2.73008600	-0.00001000
H	-2.82255800	2.60595100	0.00000400
H	-4.99947900	1.13035100	0.00003500
H	3.66983300	2.32406000	0.00001200
H	5.46609000	0.60212800	0.00002400
H	4.89858700	-1.79753300	0.00001600
H	2.52902500	-2.53944000	-0.00000300

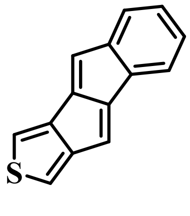

Benzo[4,5]pentaleno[1,2-*c*]thiophene (**BP*c*T**)

E = -936.608581942 Hartree

	X	Y	Z
C	1.65650600	-0.53594300	0.00000100
C	1.90707500	0.89529400	0.00000000
C	0.62073000	1.60241900	-0.00000100
C	-0.36571200	0.66734000	-0.00000100
C	0.20631100	-0.69683000	0.00000000
C	-1.81936000	0.52255700	-0.00000100
C	-2.07883500	-0.88051400	0.00000000
C	-0.79634400	-1.60879800	0.00000100
C	2.79845300	-1.27351700	0.00000100
S	4.21140200	-0.24039800	0.00000000
C	3.23216200	1.20852900	-0.00000100
C	-3.38603200	-1.34854500	-0.00000200
C	-4.43778700	-0.42134900	-0.00000200
C	-4.18290100	0.94878100	0.00000200
C	-2.86632100	1.43142800	0.00000200
H	0.50687000	2.67827900	-0.00000100
H	-0.71017200	-2.68743100	0.00000600
H	2.92707900	-2.34518200	0.00000200
H	3.71229500	2.17527600	-0.00000200
H	-3.59586600	-2.41281900	-0.00000200
H	-5.46253200	-0.77557700	-0.00000500
H	-5.01021600	1.64925900	0.00000500
H	-2.67756400	2.49945300	0.00000500

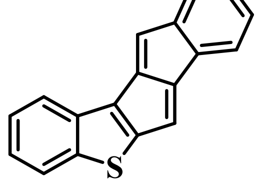

Benzo[*b*]benzo[4,5]pentaleno
[1,2-*d*]thiophene (**BP*b*BT**)

E = -1090.28646528 Hartree

	X	Y	Z
C	-0.80454700	-0.00259300	-0.00007900
C	-0.79670000	-1.38473200	-0.00006500
C	0.57185400	-1.91804500	-0.00004000
C	1.40020300	-0.84008500	-0.00004200

C	0.59876600	0.40210100	-0.00006600
C	2.81282900	-0.44807700	0.00000000
C	2.82801000	0.97793100	0.00000400
C	1.43129200	1.47060300	-0.00004300
C	-2.11984100	0.55666700	-0.00002400
C	-3.10652800	-0.46935600	0.00003700
C	-2.54873700	1.89645100	-0.00004200
C	-3.90427500	2.18487000	0.00000200
C	-4.86199500	1.15682100	0.00005800
C	-4.46992500	-0.17632000	0.00007100
S	-2.38804900	-2.08416100	0.00001700
C	3.99925900	-1.15720500	0.00001700
C	5.21607600	-0.44822900	0.00004800
C	5.23050400	0.94005200	0.00005300
C	4.02595600	1.66891400	0.00002800
H	0.83542900	-2.96638600	-0.00003200
H	1.16186400	2.51908500	-0.00004700
H	-1.81786200	2.69698700	-0.00008400
H	-4.23247700	3.21813900	-0.00000500
H	-5.91723700	1.40384500	0.00009100
H	-5.20895600	-0.96928900	0.00011400
H	4.00238100	-2.24162200	0.00001500
H	6.15167500	-0.99593200	0.00006900
H	6.17636900	1.46955700	0.00008000
H	4.04438900	2.75358200	0.00003100

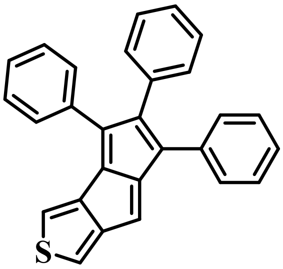

4,5,6-Triphenylpentaleno[1,2-*c*]thiophene (**III**)

E = -1476.24845782 Hartree

	X	Y	Z
C	-2.73701200	-1.10068600	-0.01121500
C	-2.54000600	-2.54201000	-0.01110800
C	-1.10170900	-2.82229800	-0.01521400
C	-0.44868900	-1.62671100	-0.01086500
C	-1.40015700	-0.51270200	-0.02896300
C	0.90225700	-1.07064200	0.00092400
C	0.75886700	0.29985300	0.00468600
C	-0.70237600	0.65822200	-0.00987200
C	-4.05026700	-0.75254900	0.00630800
S	-5.08257200	-2.17337100	0.03038000
C	-3.70307100	-3.24786500	0.00941800
C	1.85264600	1.29337800	0.01084700
C	2.12977900	-1.88406300	-0.05578000
C	-1.27101400	2.01460600	0.03968000
C	-2.35979300	2.34969200	-0.78235100
C	-2.93805300	3.61430900	-0.72351800
C	-2.44486600	4.56987400	0.16444100
C	-1.36530400	4.25079100	0.98706400
C	-0.77887700	2.99016800	0.92211100
C	1.90258100	2.31746500	-0.94789200
C	2.94367800	3.24203200	-0.94974500
C	3.94836200	3.17071000	0.01440300
C	3.90499000	2.16498700	0.97947300
C	2.86915000	1.23514000	0.97687600
C	2.26233200	-3.01981700	0.76067200
C	3.40401900	-3.81514800	0.70275800
C	4.43702600	-3.49652900	-0.17692300
C	4.31647900	-2.37567600	-0.99831600
C	3.17746000	-1.57874800	-0.93956000
H	-0.66070400	-3.81000200	-0.01194700
H	-4.50223500	0.22701200	0.01532200
H	-3.85838500	-4.31623700	0.01490000
H	-2.73669700	1.61506100	-1.48489400
H	-3.77093300	3.85579400	-1.37487200
H	-2.89587000	5.55459800	0.21285000
H	-0.97718800	4.98634200	1.68287900
H	0.05683200	2.75467200	1.56917500
H	1.12583000	2.38108700	-1.70102200
H	2.96867900	4.02024600	-1.70453500
H	4.75621400	3.89385000	0.01524300
H	4.67880200	2.10470000	1.73684500
H	2.83971900	0.45666300	1.73012400
H	1.47259900	-3.26530700	1.46173700
H	3.48843000	-4.68184100	1.34930500
H	5.32559300	-4.11589400	-0.22423300

H	5.11062200	-2.12391100	-1.69262100
H	3.08924400	-0.71761000	-1.59037700

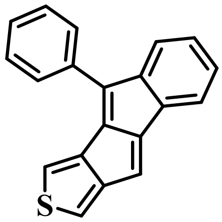

4-Phenylbenzo[4,5]pentaleno
[1,2-*c*]thiophene (**VI**)

E = -1167.72273738 Hartree

	X	Y	Z
C	1.91962100	-0.07084600	-0.01994200
C	2.62838300	-1.34042200	-0.05646400
C	1.64990900	-2.43037000	-0.03448400
C	0.41150300	-1.86907000	0.01575900
C	0.49895100	-0.40005100	0.03063600
C	-1.00414400	-2.21834200	0.03095500
C	-1.72142600	-0.98677900	0.03995500
C	-0.75284200	0.14555400	0.02931100
C	2.75762400	1.00098600	-0.05278000
S	4.42895900	0.48991800	-0.13392900
C	3.98170700	-1.19971000	-0.11765100
C	-3.10944100	-0.99642800	0.10276900
C	-3.78133700	-2.22663800	0.12936900
C	-3.07706000	-3.42839500	0.10315800
C	-1.67677200	-3.43077700	0.05998400
C	-1.10780900	1.57174900	0.02201700
C	-2.12275200	2.06714200	-0.81318300
C	-2.43247100	3.42427200	-0.82900100
C	-1.74422800	4.31271200	-0.00410100
C	-0.73766300	3.83386000	0.83417800
C	-0.42058700	2.47900900	0.84490400
H	1.89273400	-3.48393100	-0.06732400
H	2.52714800	2.05493700	-0.04385300
H	4.75139800	-1.95547500	-0.15656300
H	-3.67331300	-0.07228200	0.14263900
H	-4.86450600	-2.24085300	0.17548100
H	-3.61563500	-4.36891700	0.12475200
H	-1.13168000	-4.36838000	0.05574000

H	-2.64947300	1.38930000	-1.47407300
H	-3.21041300	3.78942600	-1.49032700
H	-1.99055800	5.36840200	-0.01346600
H	-0.20327400	4.51638600	1.48583000
H	0.34923700	2.10796000	1.51169800

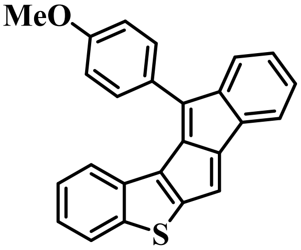

11-(4-methoxyphenyl)benzo[*b*]benzo[4,5]
pentaleno[1,2-*d*]thiophene (**V**(PMP))

E = -1435.95670948 Hartree

	X	Y	Z
C	-1.84410200	0.07259600	-0.01686900
C	-2.96379100	0.89030400	-0.04321900
C	-2.60521400	2.30650000	-0.04056200
C	-1.24553800	2.35024900	-0.01731900
C	-0.69589800	0.98420200	-0.02342400
C	-0.12301800	3.28575600	0.01111000
C	1.05641300	2.49002500	0.01860700
C	0.67012600	1.04340200	-0.01672700
C	-2.17782700	-1.32088500	0.06312000
C	-3.59046300	-1.50710900	0.06122100
C	-1.36492000	-2.46615300	0.16304100
C	-1.94416800	-3.72332100	0.23832100
C	-3.33920200	-3.88206900	0.21819900
C	-4.17097400	-2.77285600	0.13183200
S	-4.46803900	0.02373100	-0.01281800
C	-0.03795100	4.66630800	0.01189600
C	1.23307900	5.26907700	0.01935200
C	2.38508800	4.49388400	0.01218400
C	2.30379800	3.08867200	0.00399800
C	1.64789200	-0.04997400	-0.09018400
C	2.70843200	-0.14199200	0.81935400
C	3.63920800	-1.17734600	0.75226400
C	3.53565300	-2.13724200	-0.25917900
C	2.49123100	-2.04984900	-1.18895400

C	1.56167500	-1.02733400	-1.09847300
H	-3.30217300	3.13255300	-0.04632900
H	-0.28822000	-2.36216000	0.18537700
H	-1.30913600	-4.59872900	0.31566000
H	-3.77186600	-4.87406200	0.27627800
H	-5.24879600	-2.88854200	0.12597200
H	-0.93175200	5.28029400	0.00125800
H	1.31130000	6.35042000	0.02283200
H	3.35761800	4.97268600	0.00705800
H	3.20863700	2.49298200	-0.02318000
H	2.79534700	0.58925900	1.61464200
H	4.43029800	-1.22374100	1.48848600
H	2.43082100	-2.79390700	-1.97404900
H	0.76312100	-0.96616300	-1.82857400
O	4.39303600	-3.18183200	-0.42452200
C	5.47445000	-3.33050800	0.48779100
H	5.11495300	-3.47639100	1.51187900
H	6.01389600	-4.21916700	0.16502900
H	6.14641000	-2.46639600	0.45464100

S5. NMR spectra of compounds and intermediates

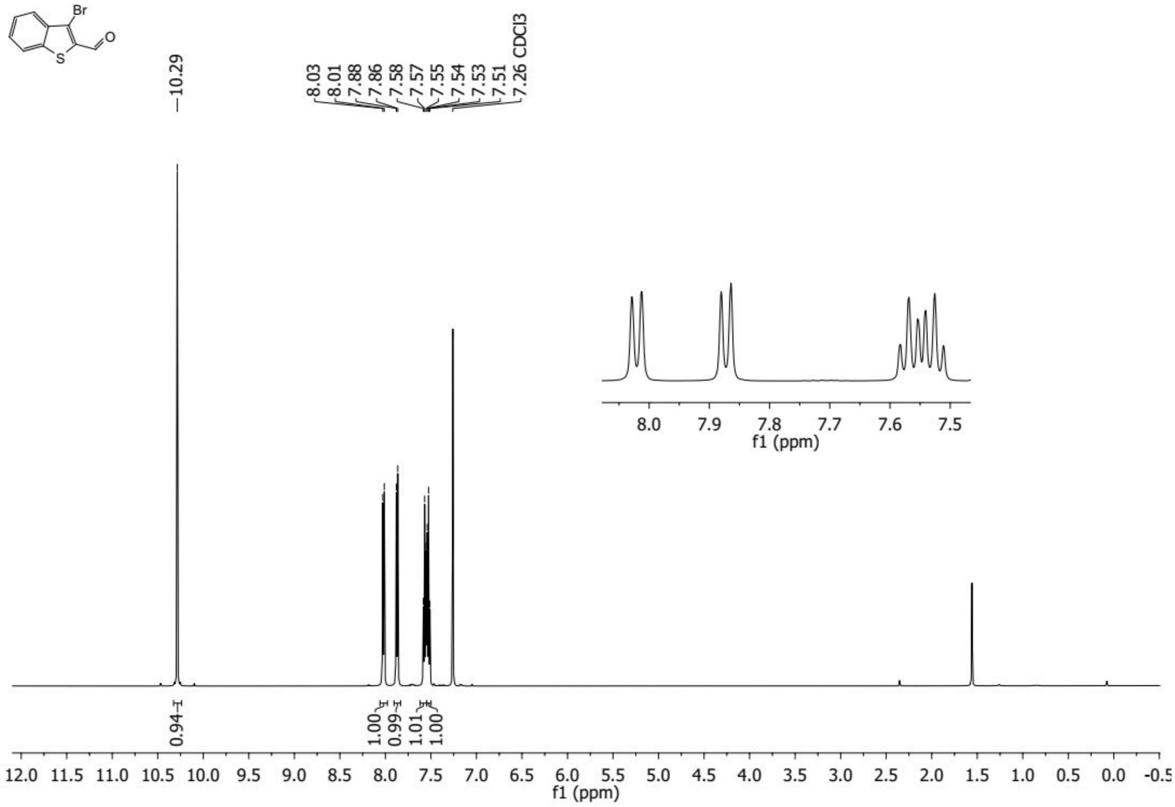

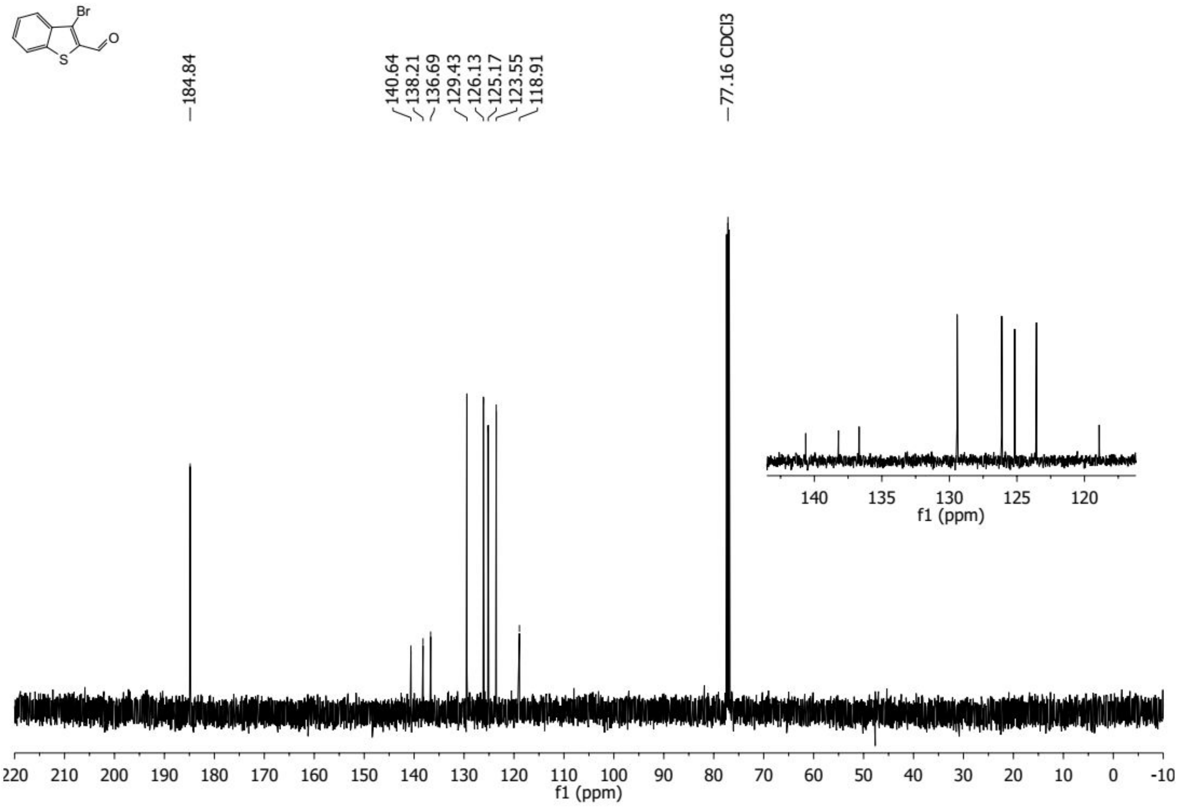

Figure S20. ^{1}H-NMR (500 MHz) (top) and ^{13}C-NMR (126 MHz) (bottom) spectra of **2** in $CDCl_3$.

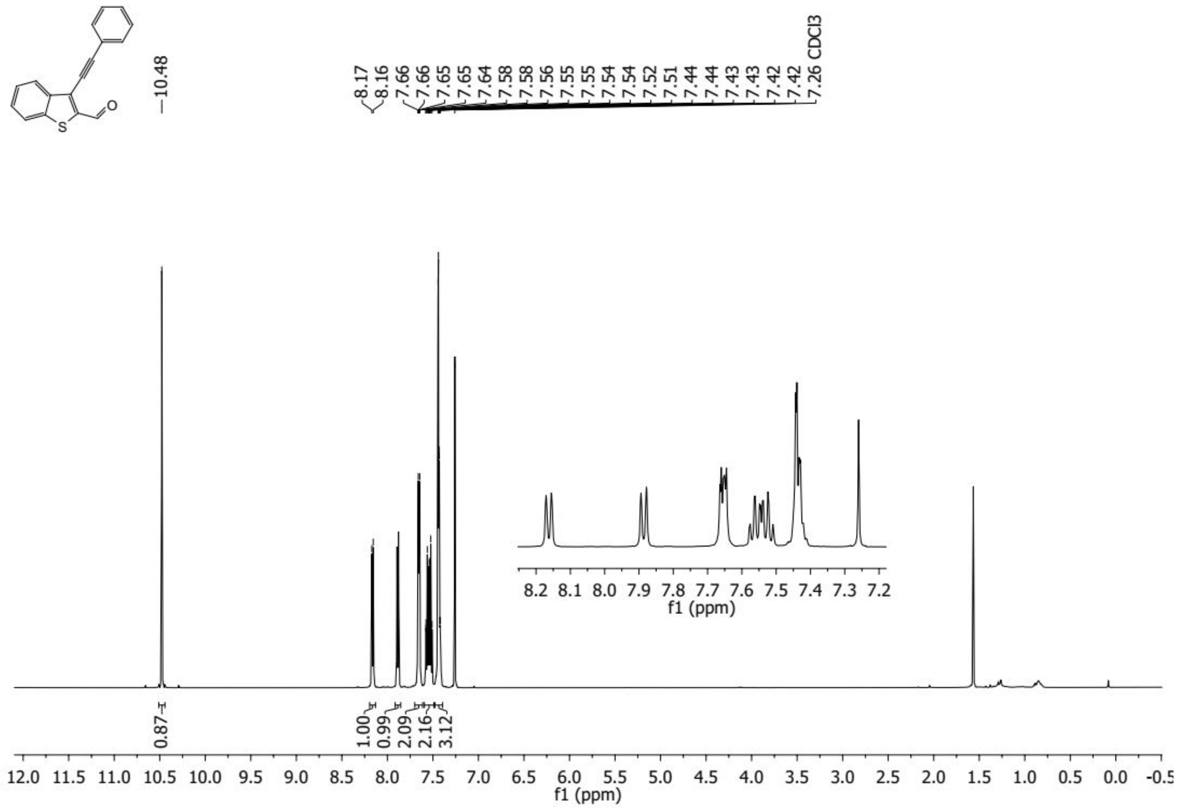

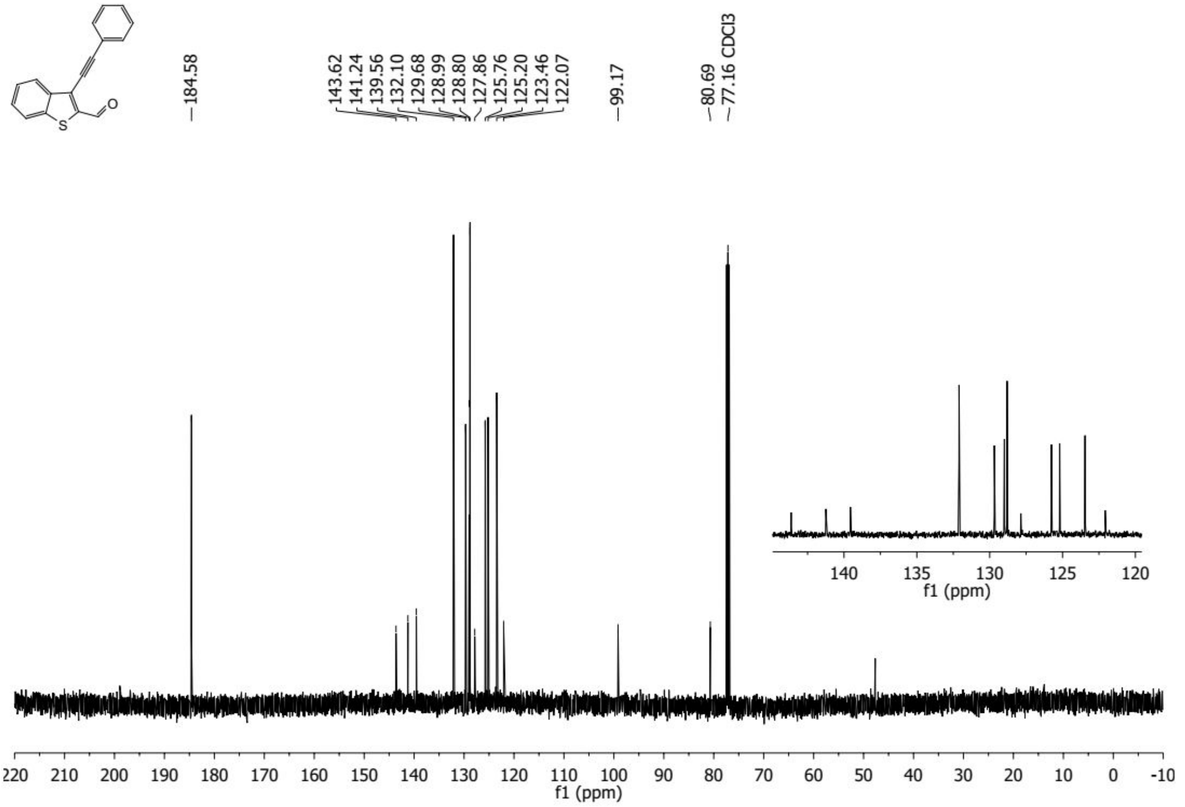

Figure S21. ^{1}H-NMR (500 MHz) (top) and ^{13}C-NMR (126 MHz) (bottom) spectra of **3** in $CDCl_3$.

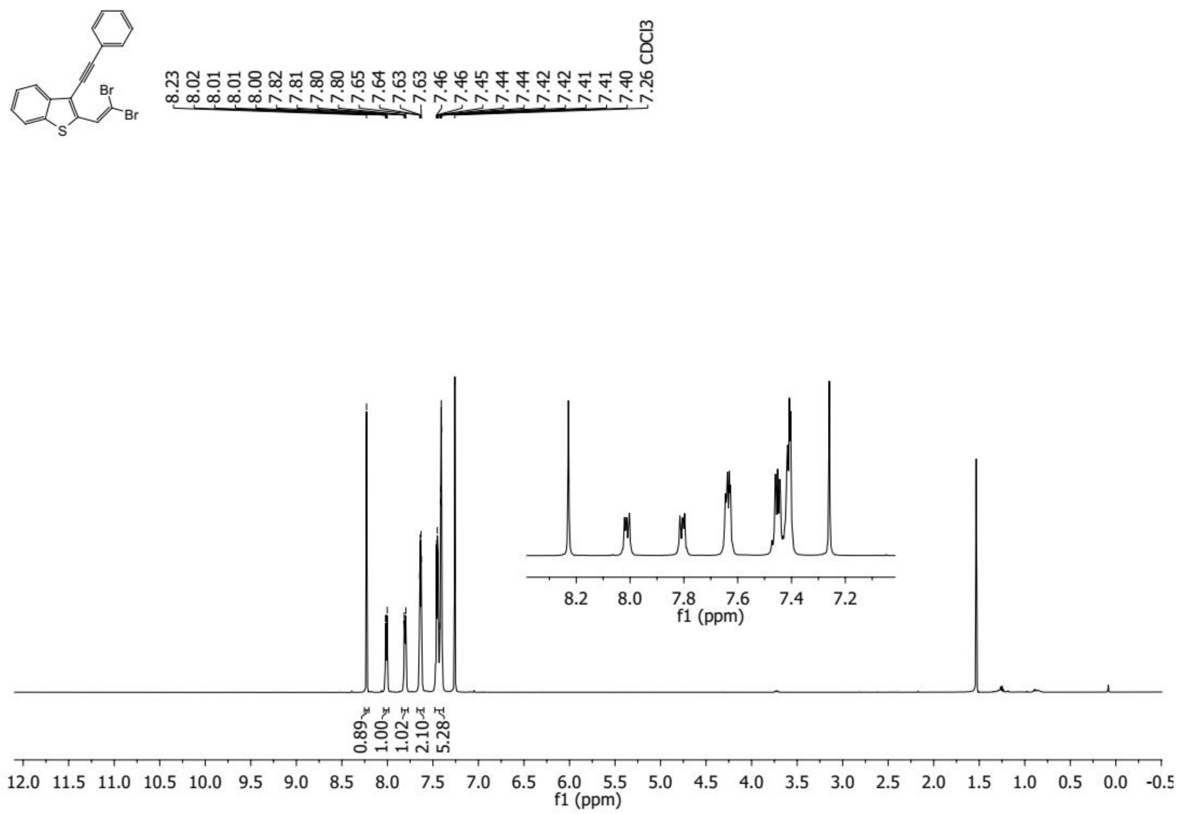

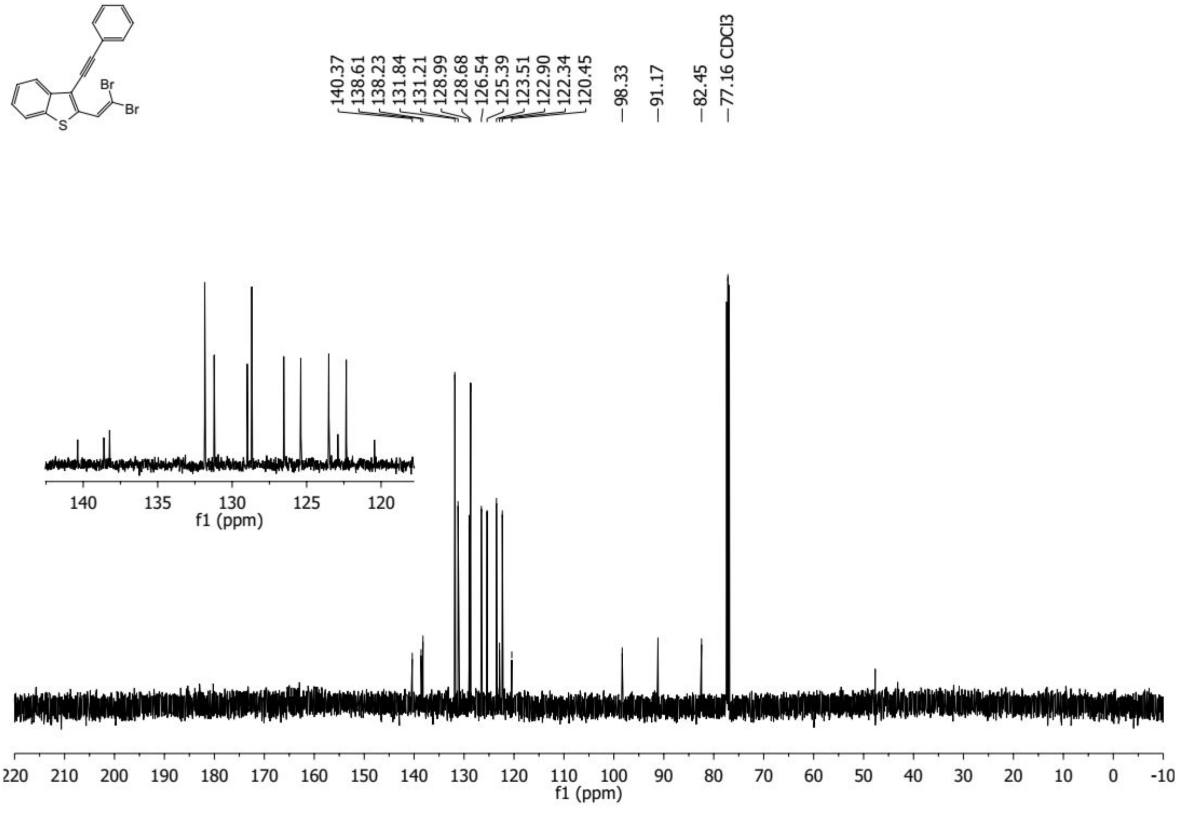

Figure S22. ^{1}H-NMR (500 MHz) (top) and ^{13}C-NMR (126 MHz) (bottom) spectra of **4** in $CDCl_3$.

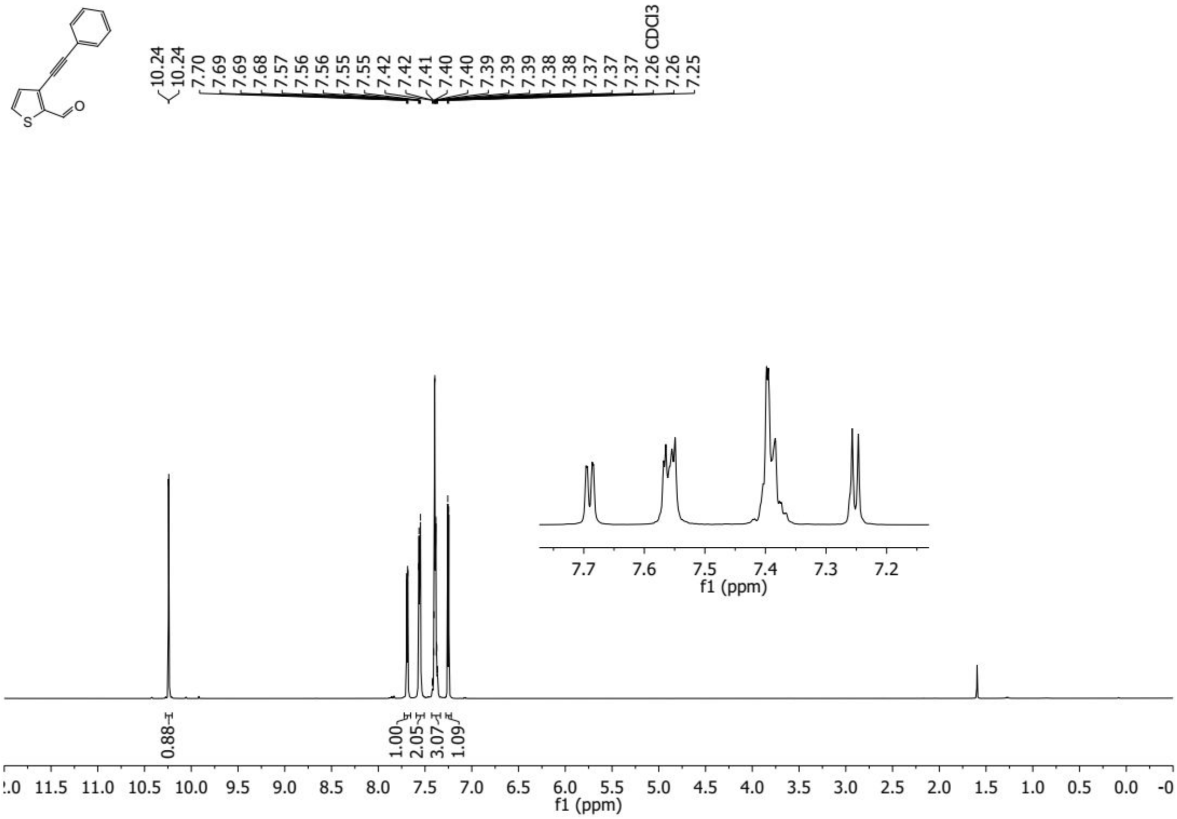

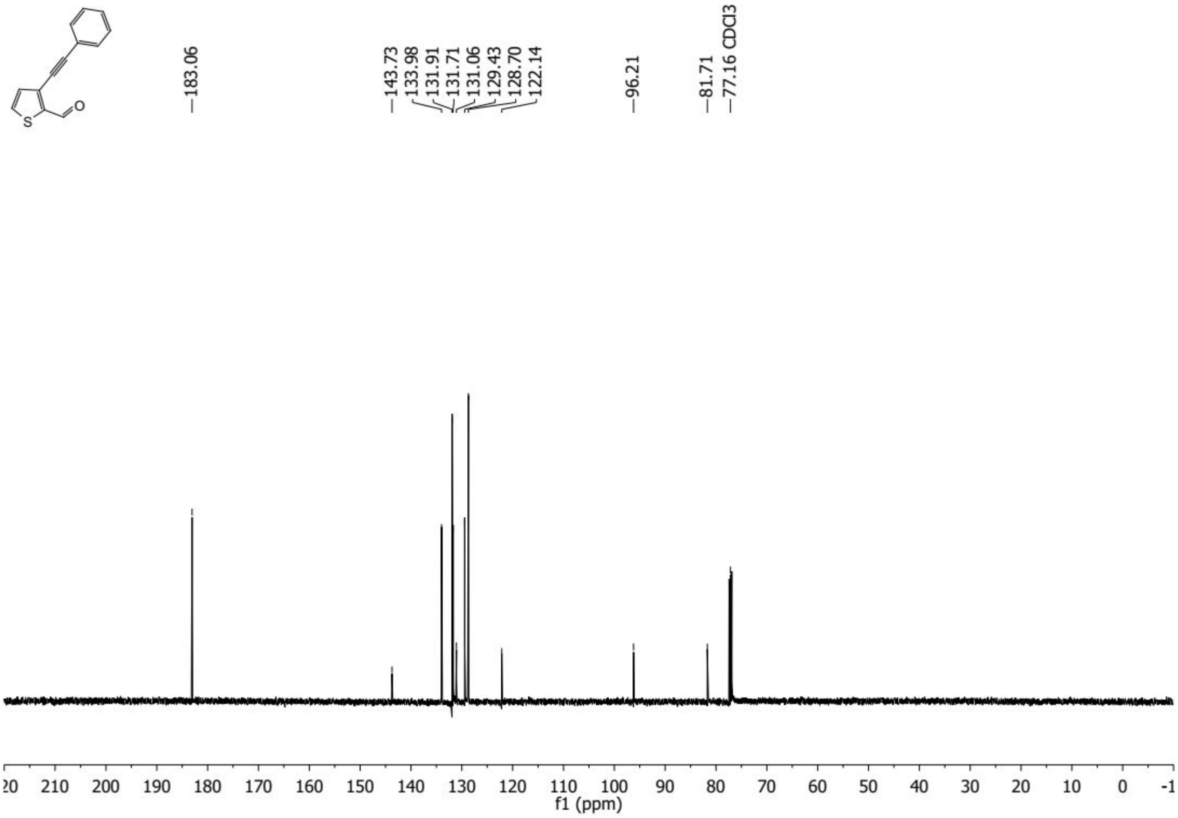

Figure S23. ^{1}H-NMR (500 MHz) (top) and ^{13}C-NMR (126 MHz) (bottom) spectra of **6** in $CDCl_3$.

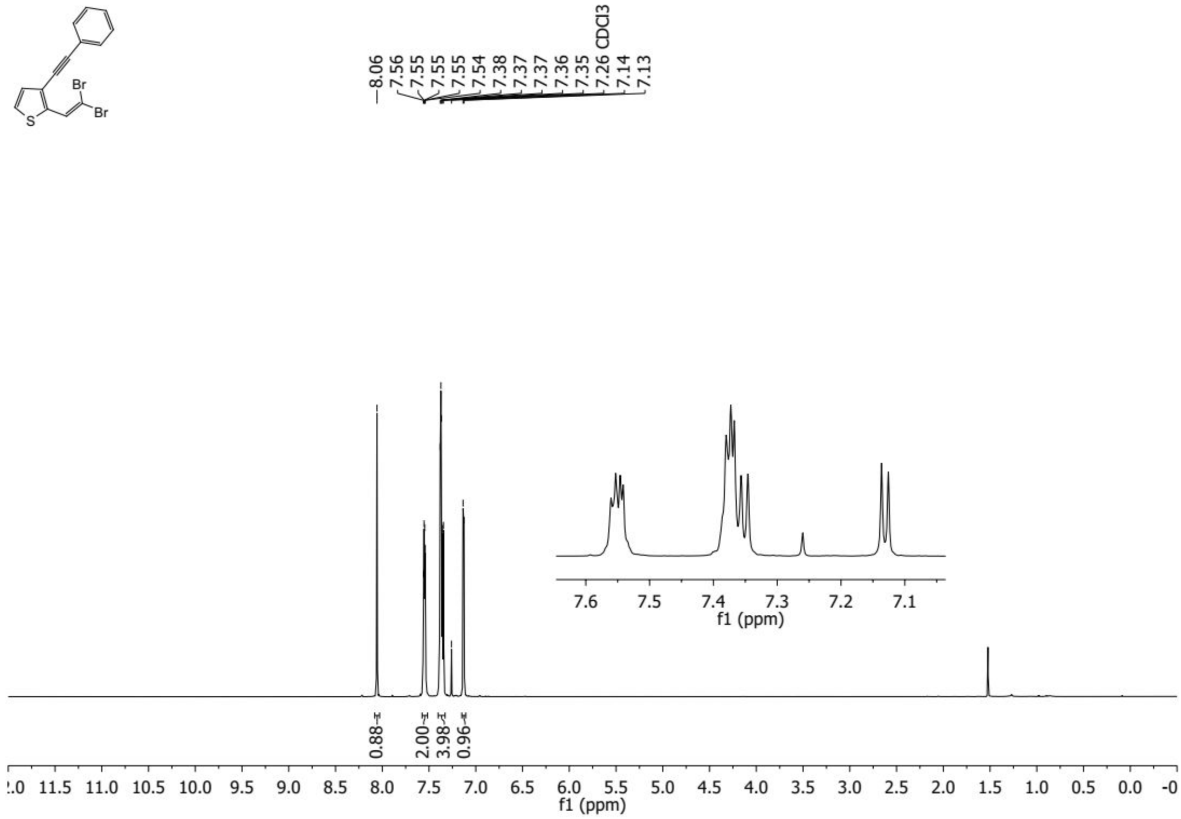

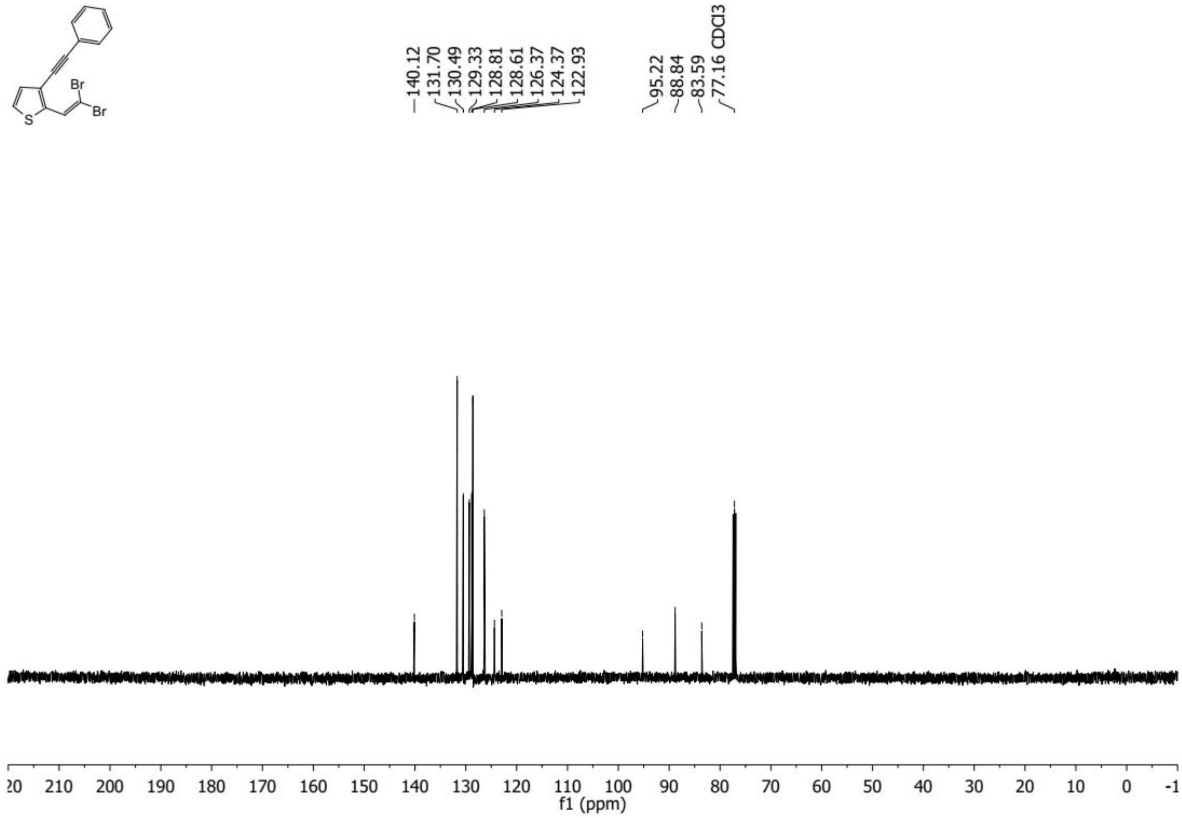

Figure S24. ^{1}H-NMR (500 MHz) (top) and ^{13}C-NMR (126 MHz) (bottom) spectra of **7** in $CDCl_3$.

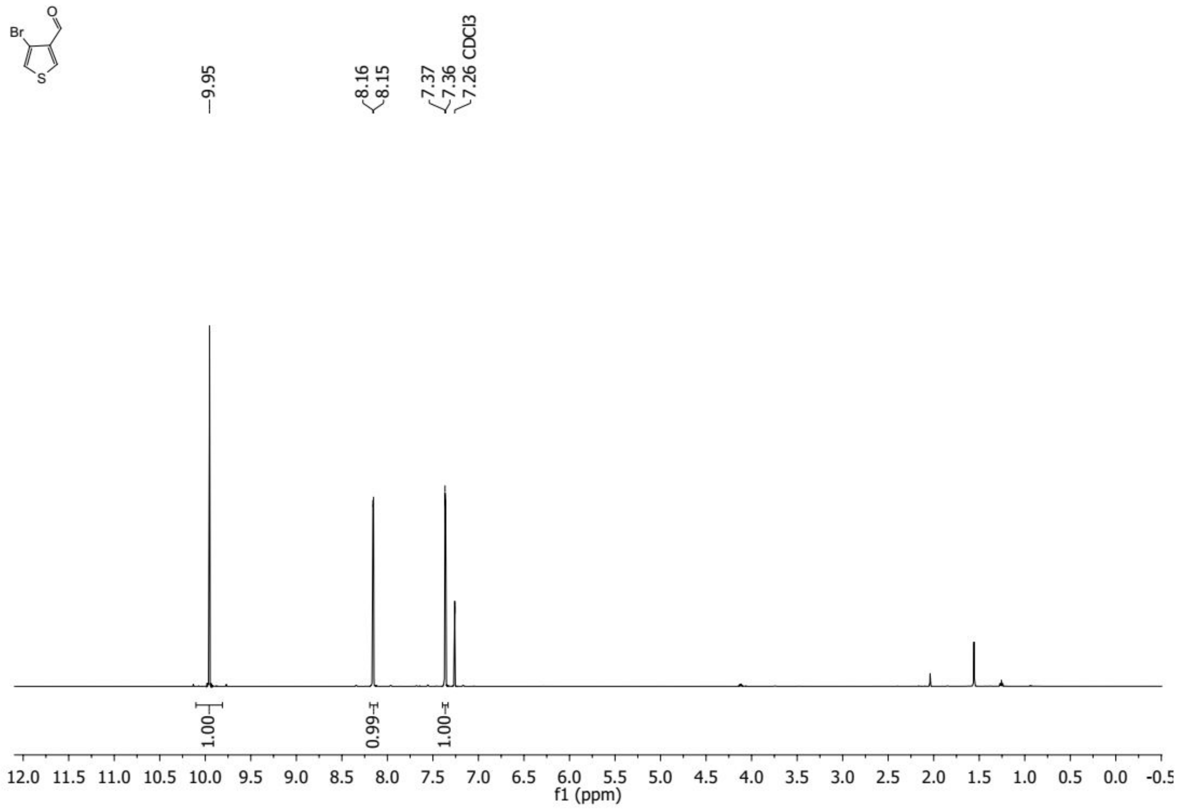

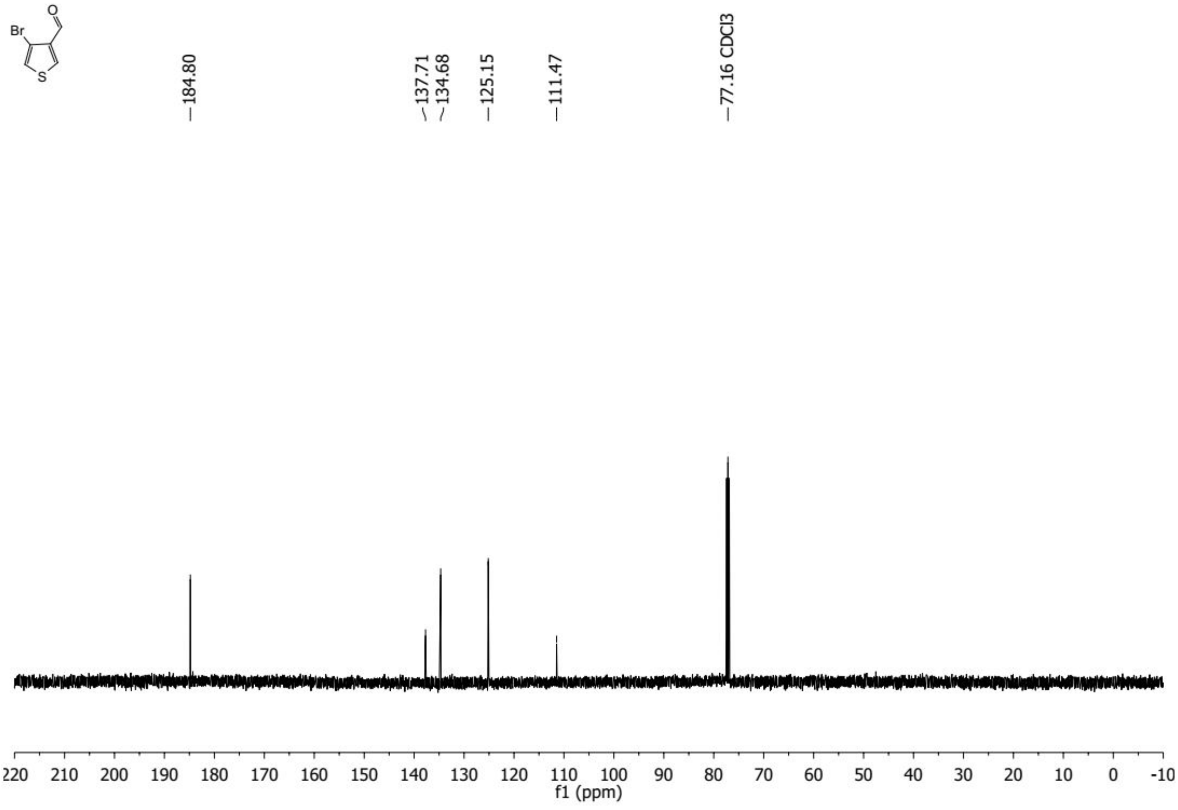

Figure S25. ^{1}H-NMR (500 MHz) (top) and ^{13}C-NMR (126 MHz) (bottom) spectra of **9** in $CDCl_3$.

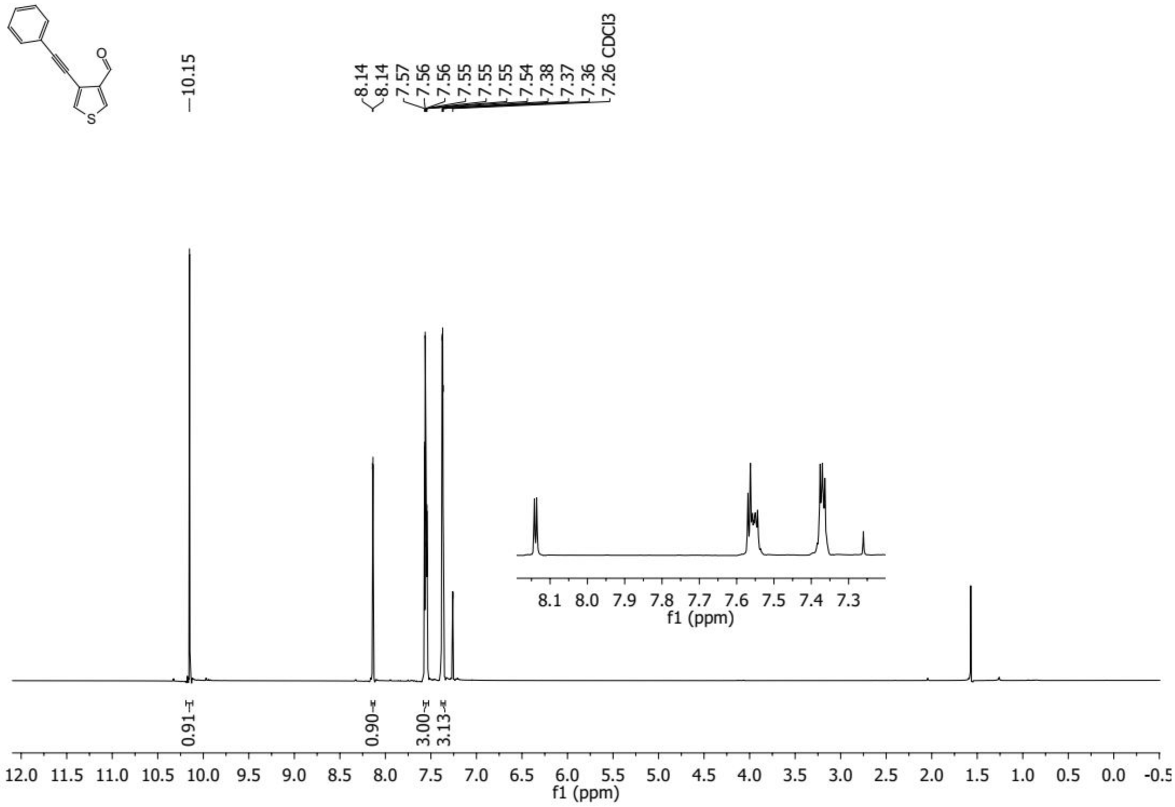

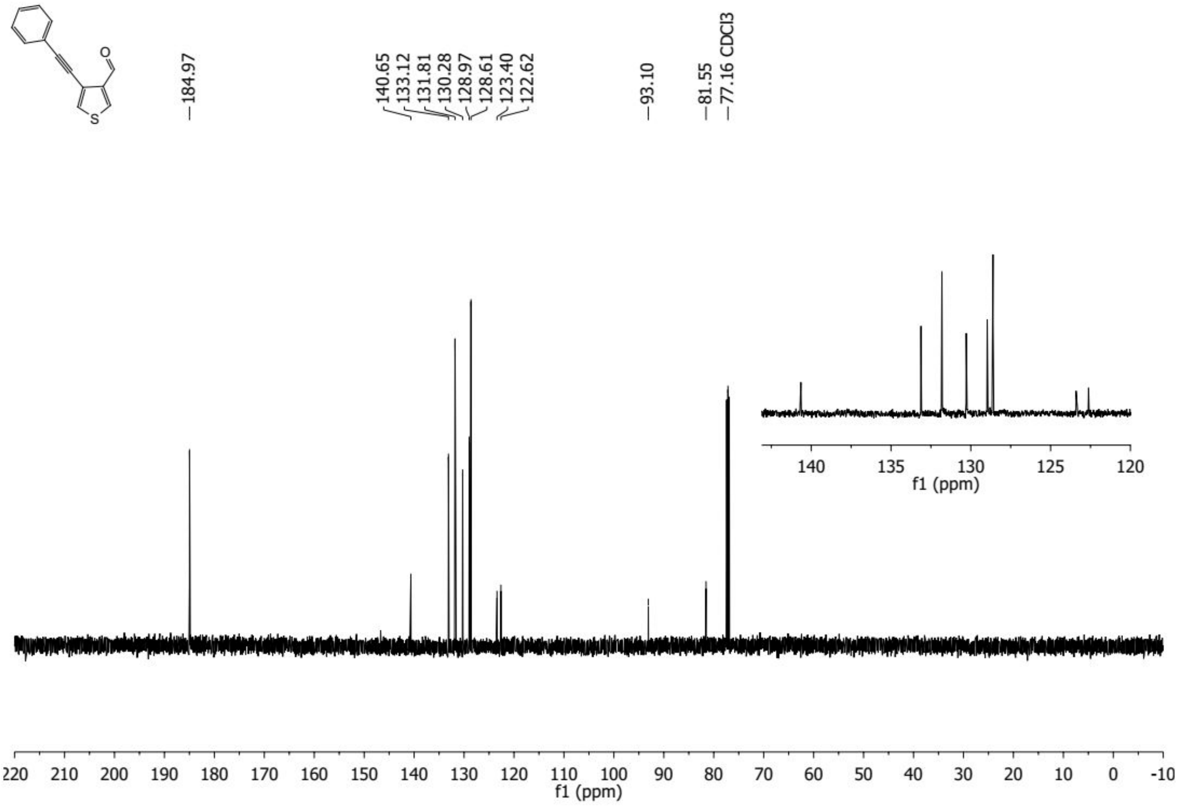

Figure S26. ^{1}H-NMR (500 MHz) (top) and ^{13}C-NMR (126 MHz) (bottom) spectra of **10** in $CDCl_3$.

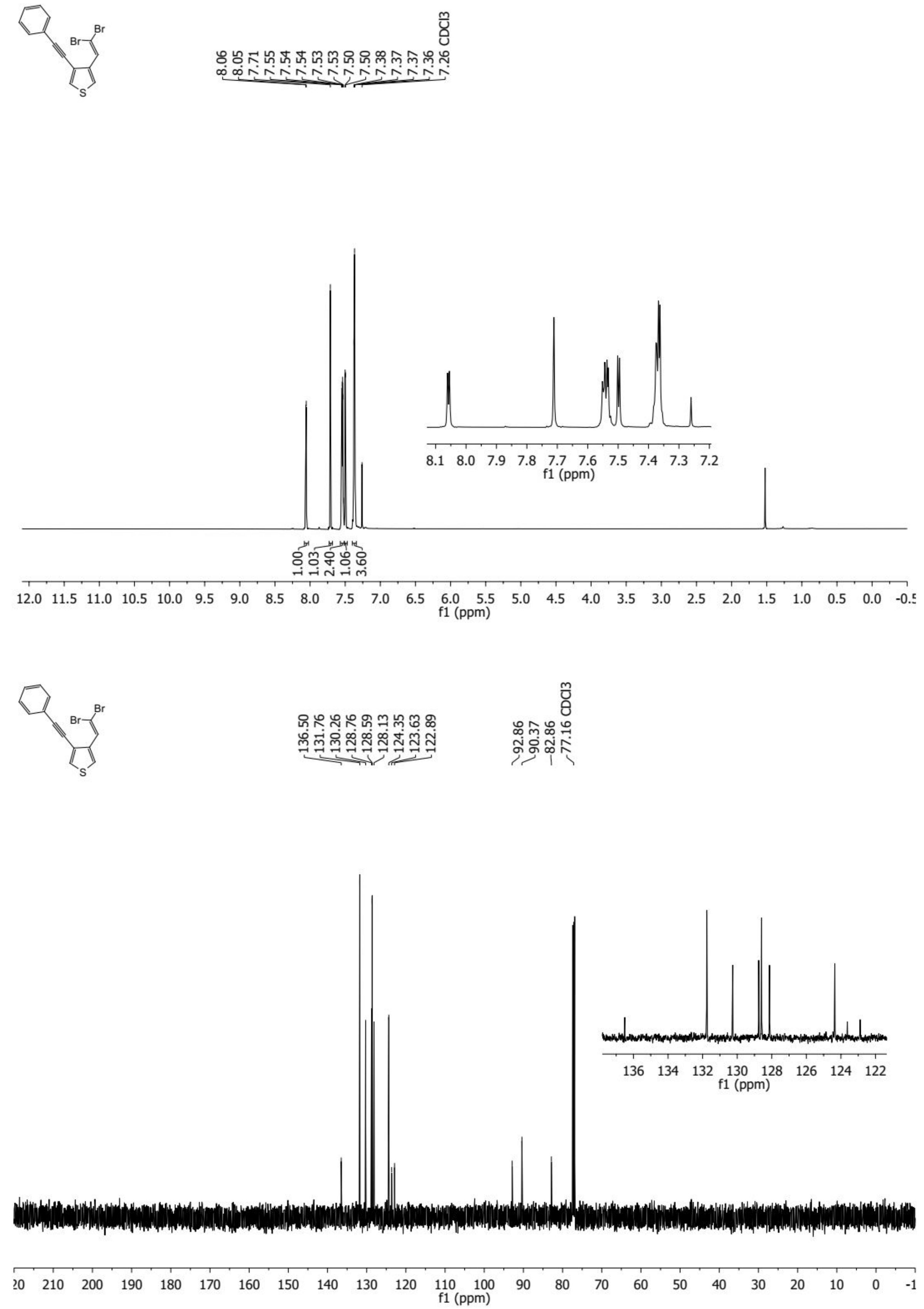

Figure S27. ^{1}H-NMR (500 MHz) (top) and ^{13}C-NMR (126 MHz) (bottom) spectra of **11** in $CDCl_3$.

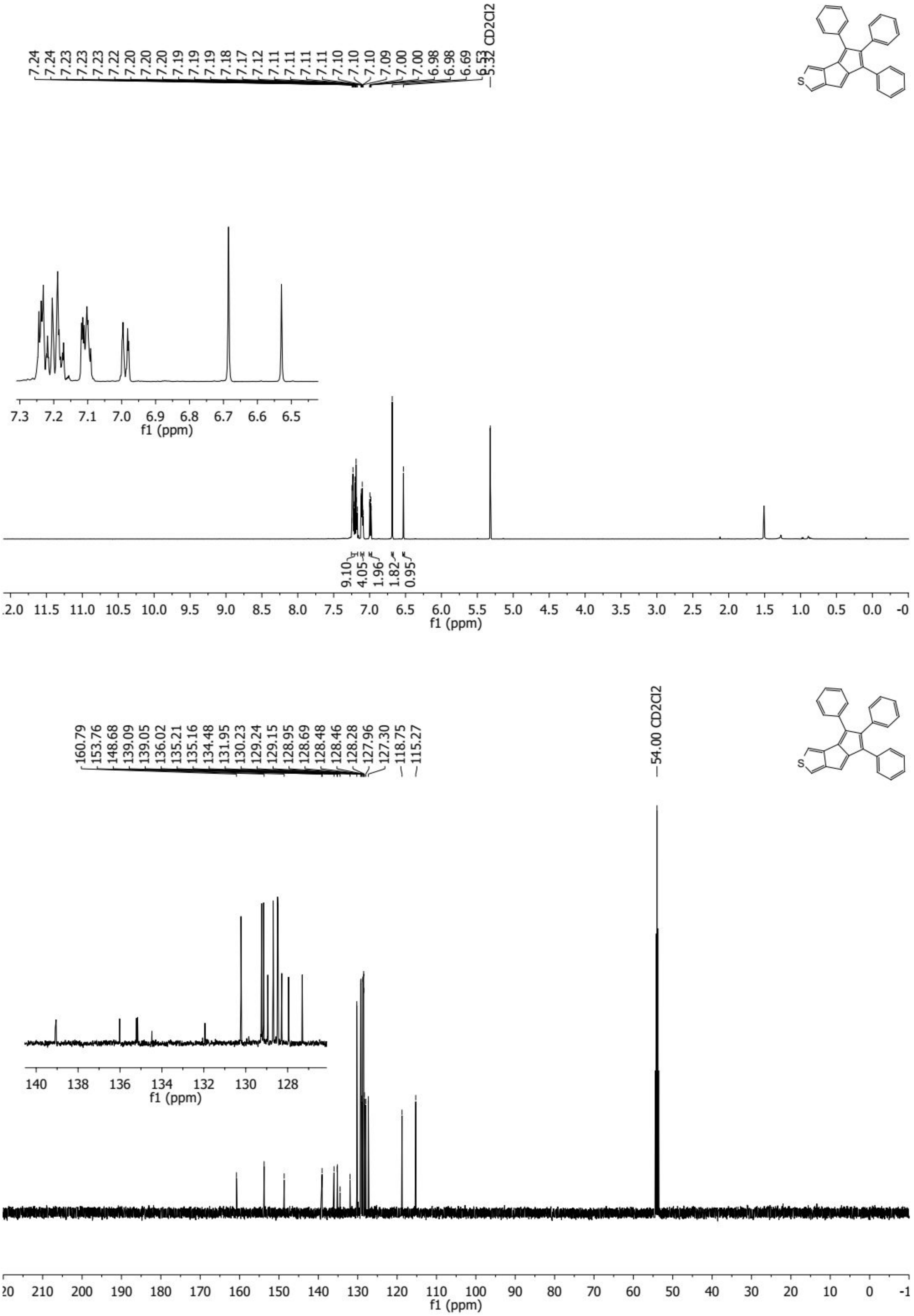

Figure S28. ^{1}H-NMR (500 MHz) (top) and ^{13}C-NMR (126 MHz) (bottom) spectra of **III** in CD_2Cl_2.

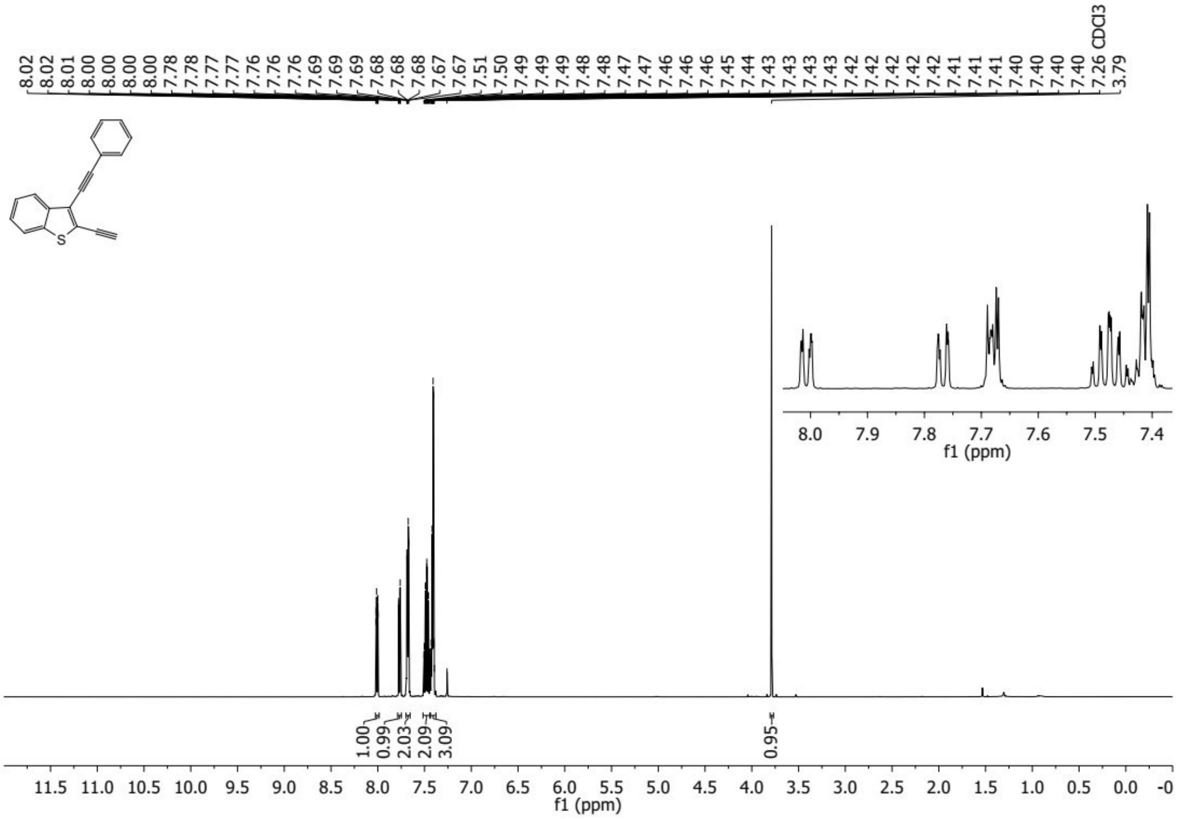

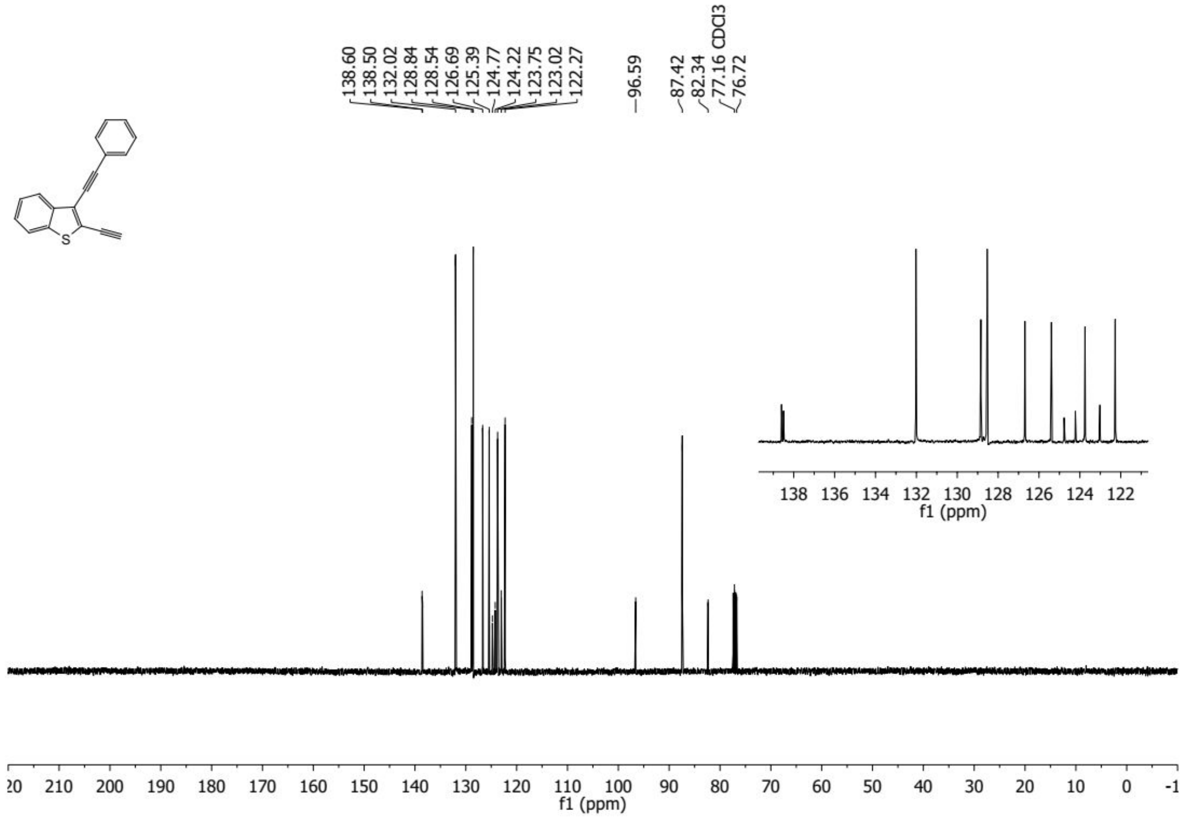

Figure S29. ^{1}H-NMR (500 MHz) (top) and ^{13}C-NMR (126 MHz) (bottom) spectra of **12** in $CDCl_3$.

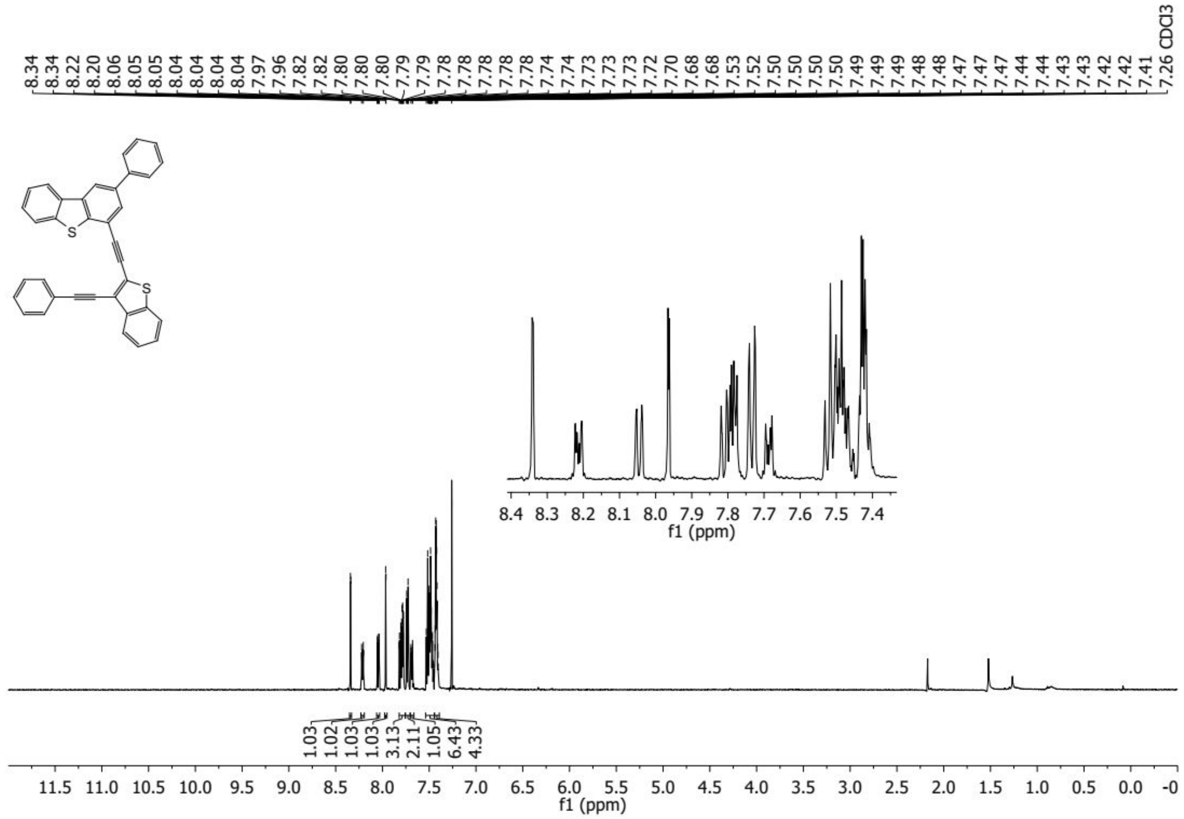

Figure S30. ^{1}H-NMR (500 MHz) spectrum of side-product of **13** in $CDCl_3$.

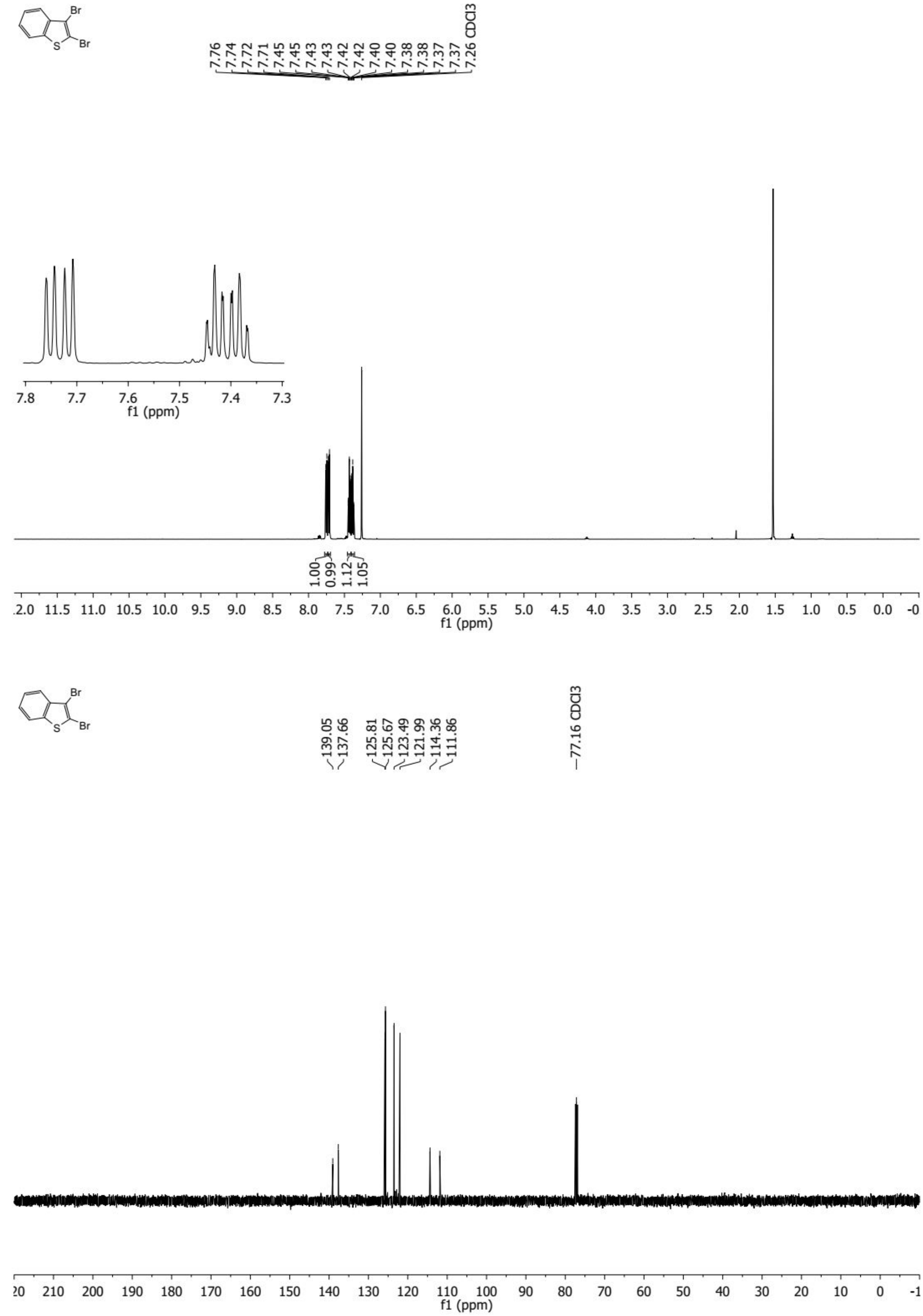

Figure S31. ^{1}H-NMR (500 MHz) (top) and ^{13}C-NMR (126 MHz) (bottom) spectra of **15** in $CDCl_3$.

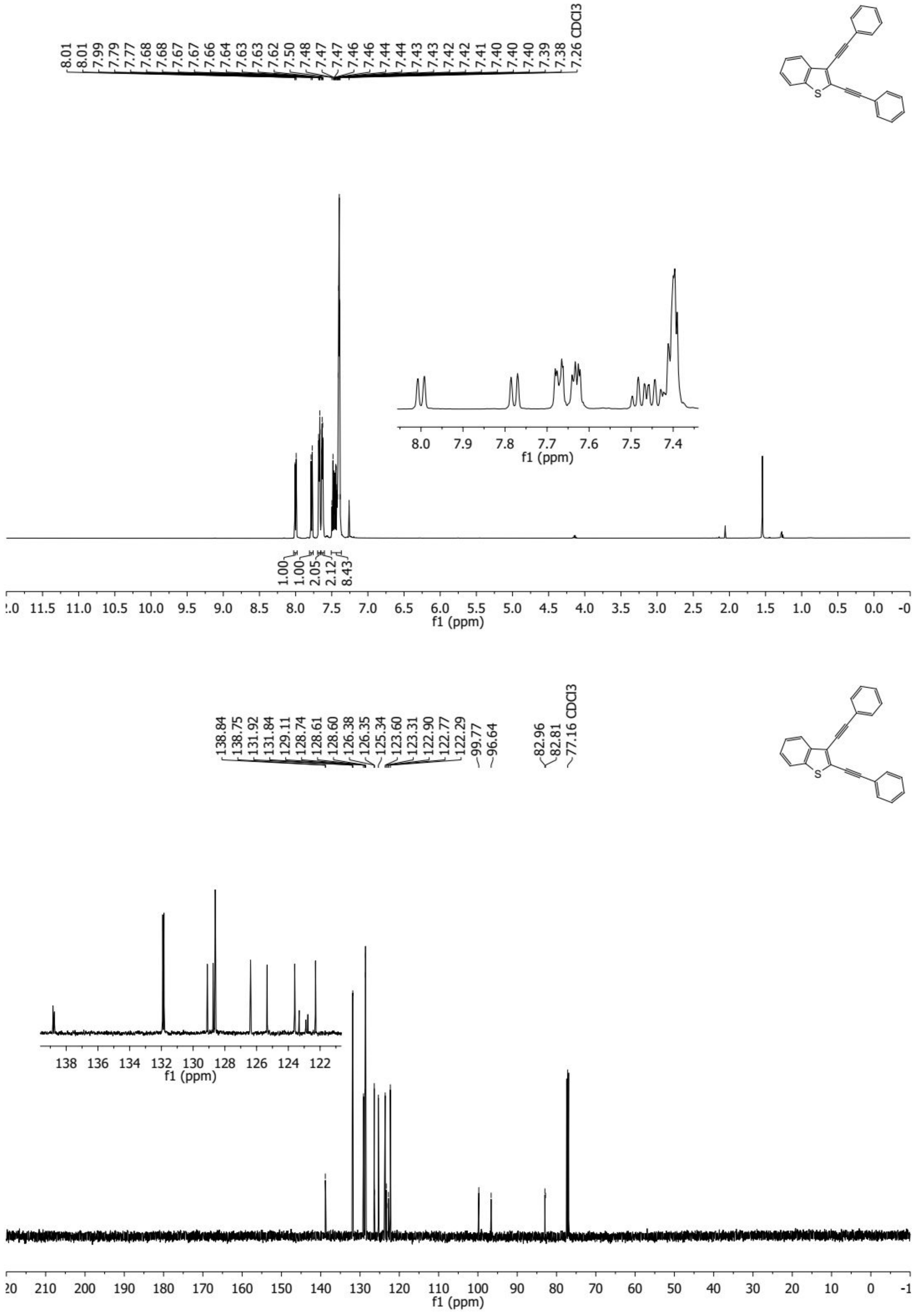

Figure S32. ^{1}H-NMR (500 MHz) (top) and ^{13}C-NMR (126 MHz) (bottom) spectra of **16** in $CDCl_3$.

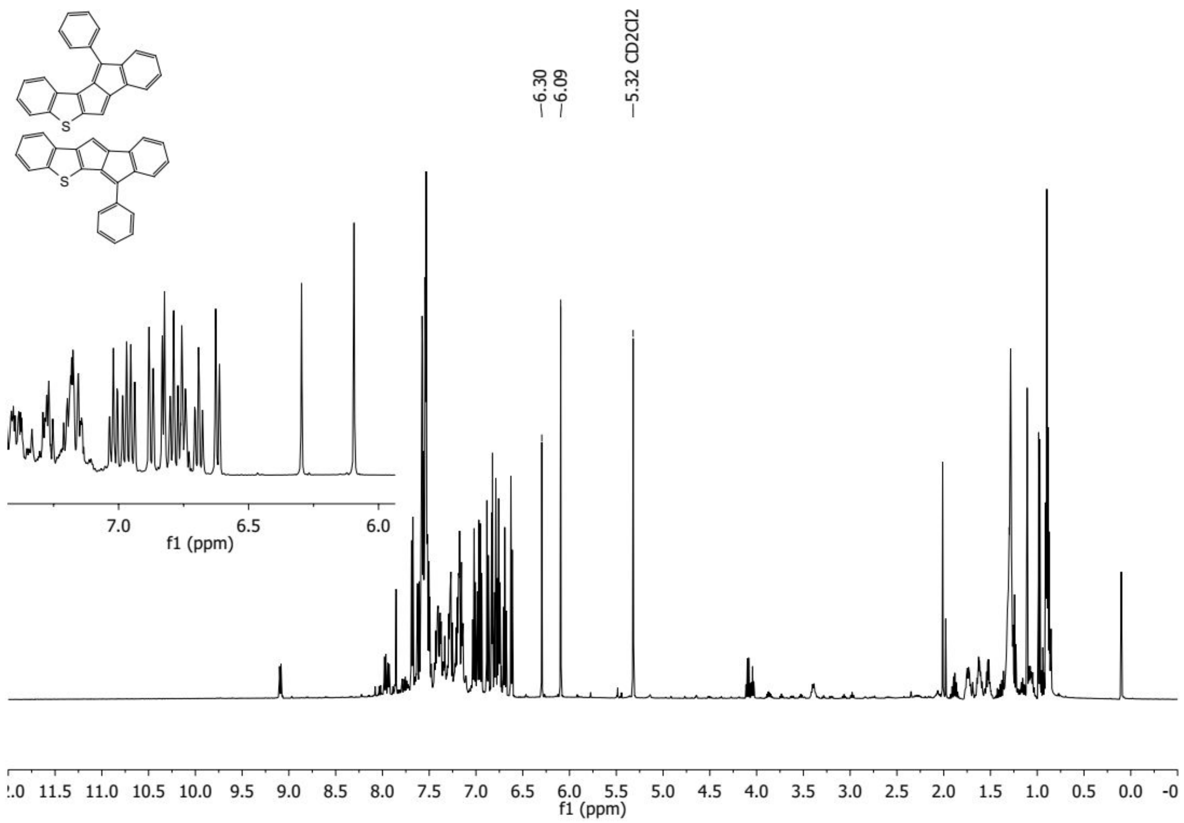

Figure S33. ^{1}H-NMR (500 MHz) spectrum the inseparable isomeric mixture of **V**(Ph) in CD_2Cl_2.

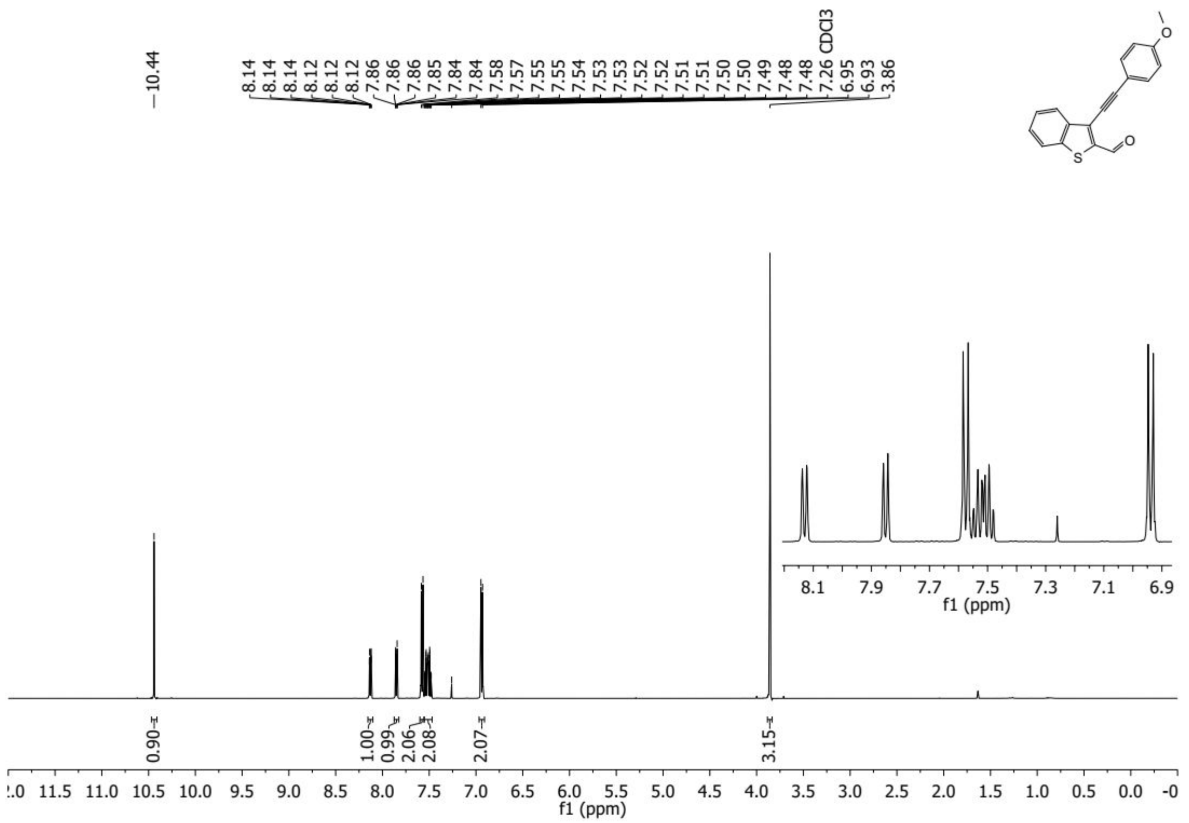

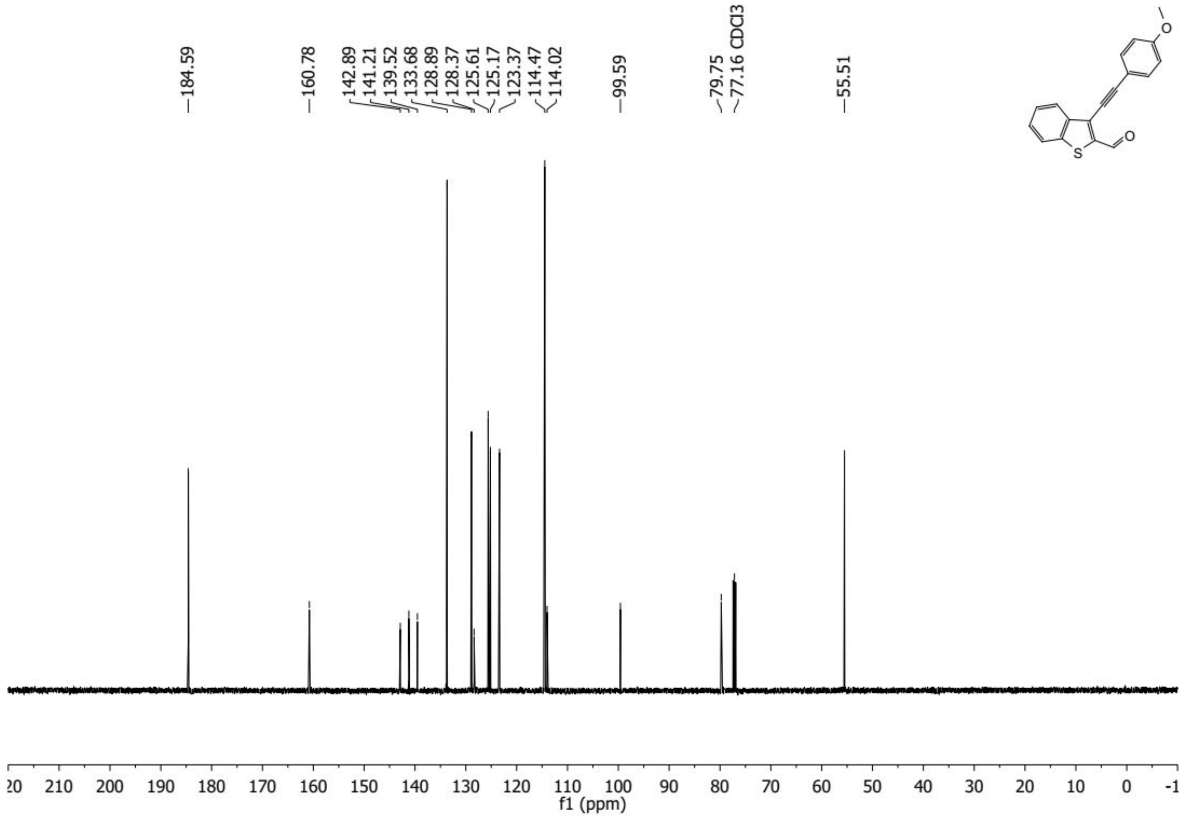

Figure S34. ^{1}H-NMR (500 MHz) (top) and ^{13}C-NMR (126 MHz) (bottom) spectra of **17** in $CDCl_3$.

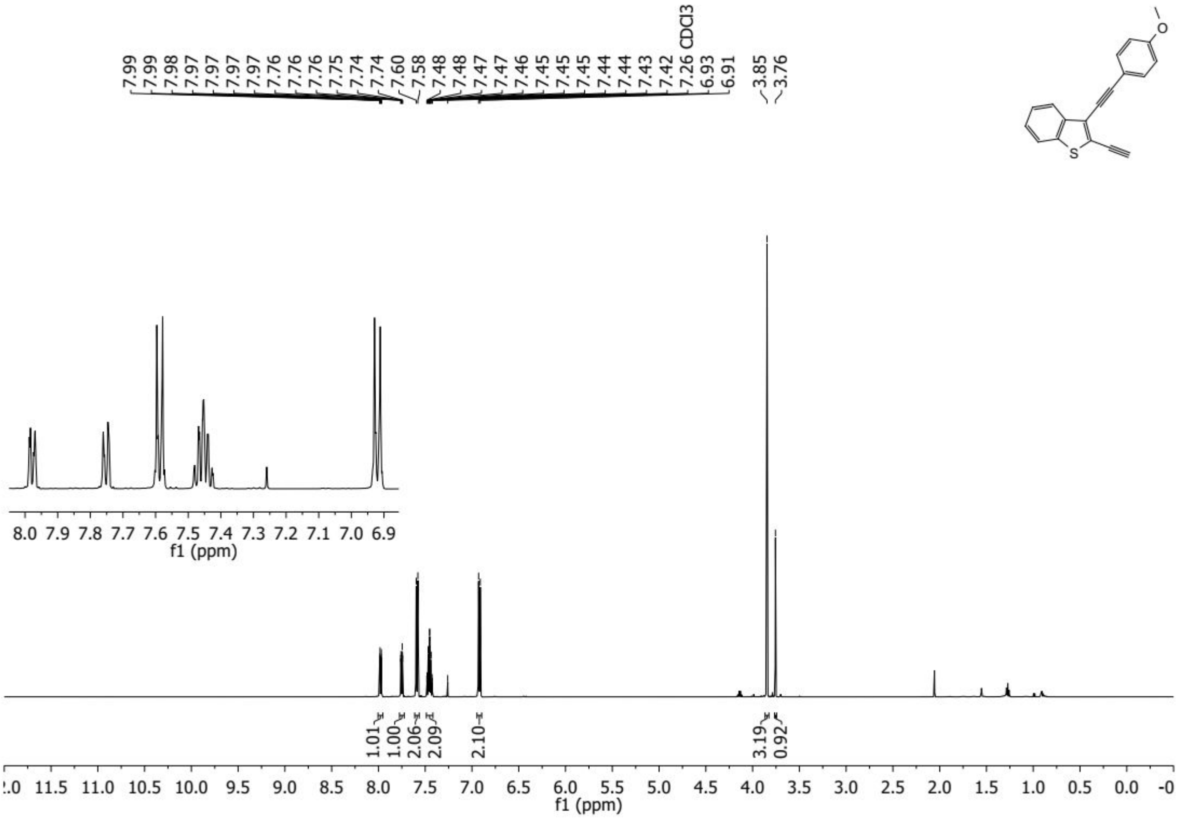

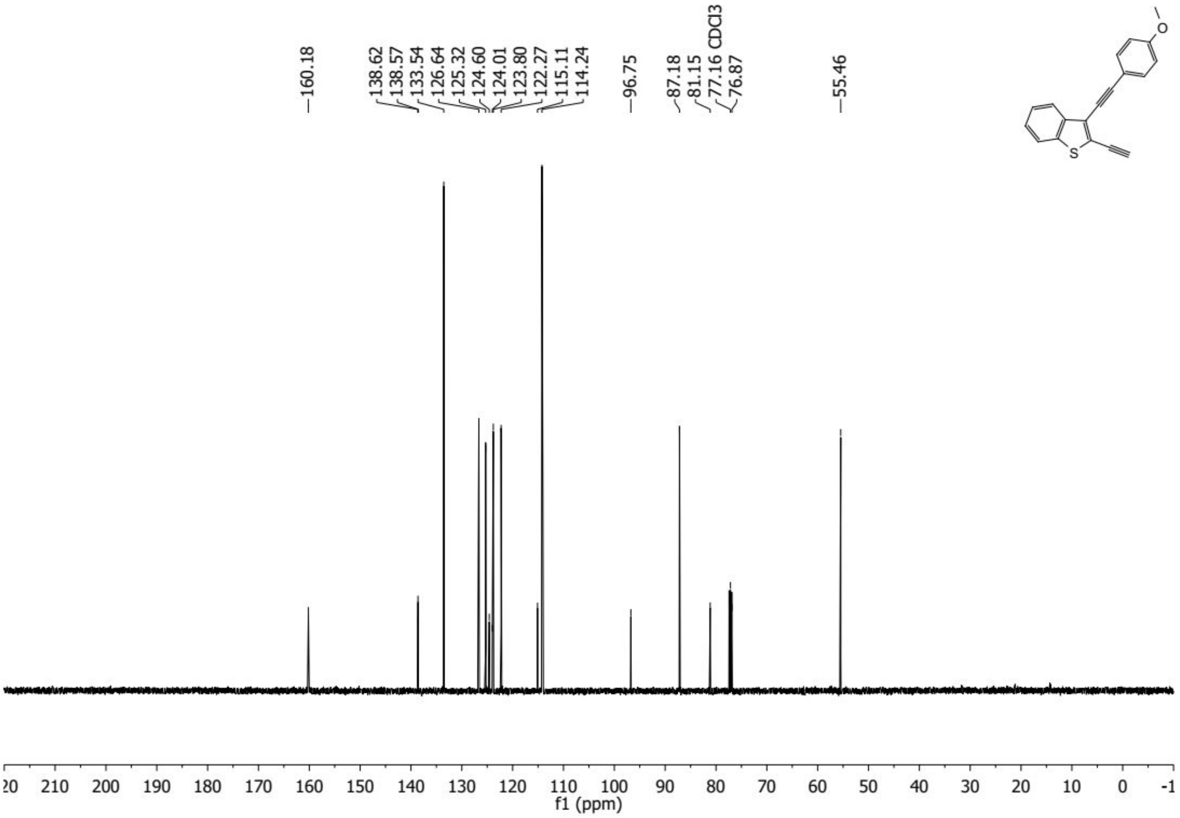

Figure S35. ^{1}H-NMR (500 MHz) (top) and ^{13}C-NMR (126 MHz) (bottom) spectra of **18** in $CDCl_3$.

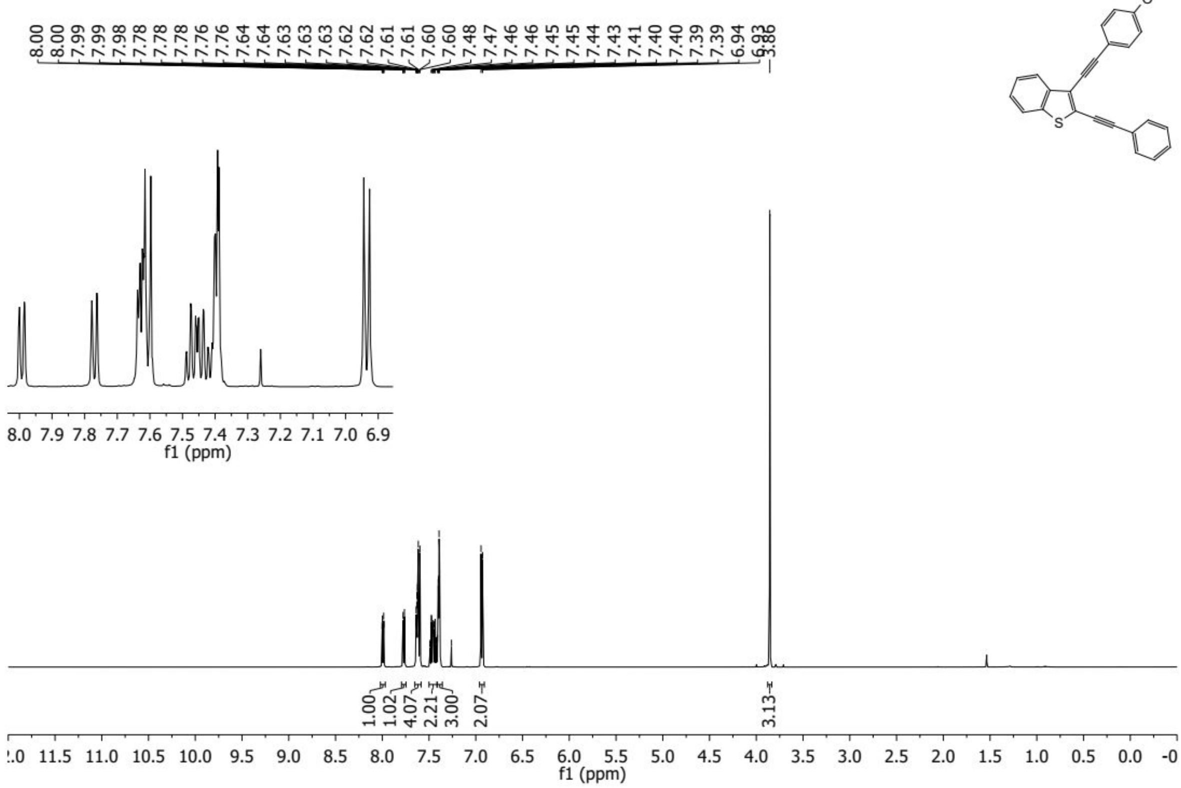

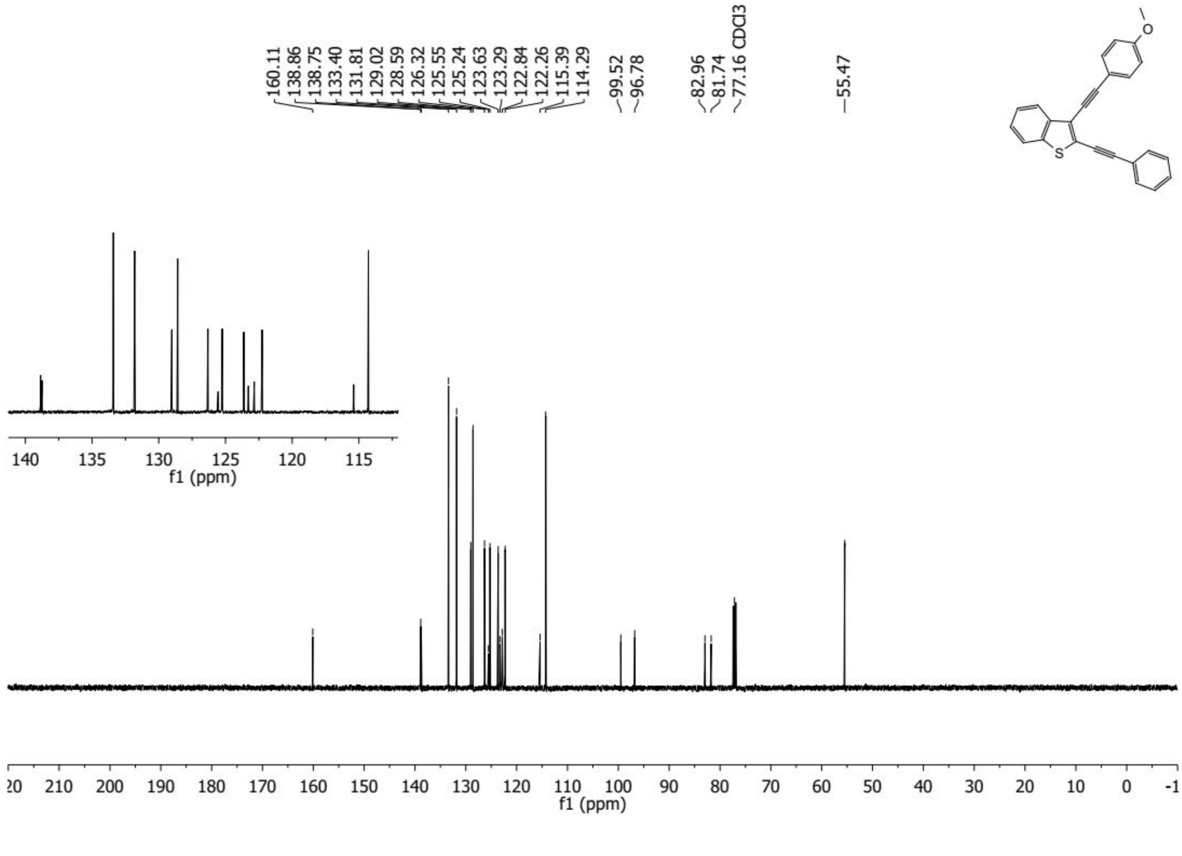

Figure S36. ^{1}H-NMR (500 MHz) (top) and ^{13}C-NMR (126 MHz) (bottom) spectra of **19** in $CDCl_3$.

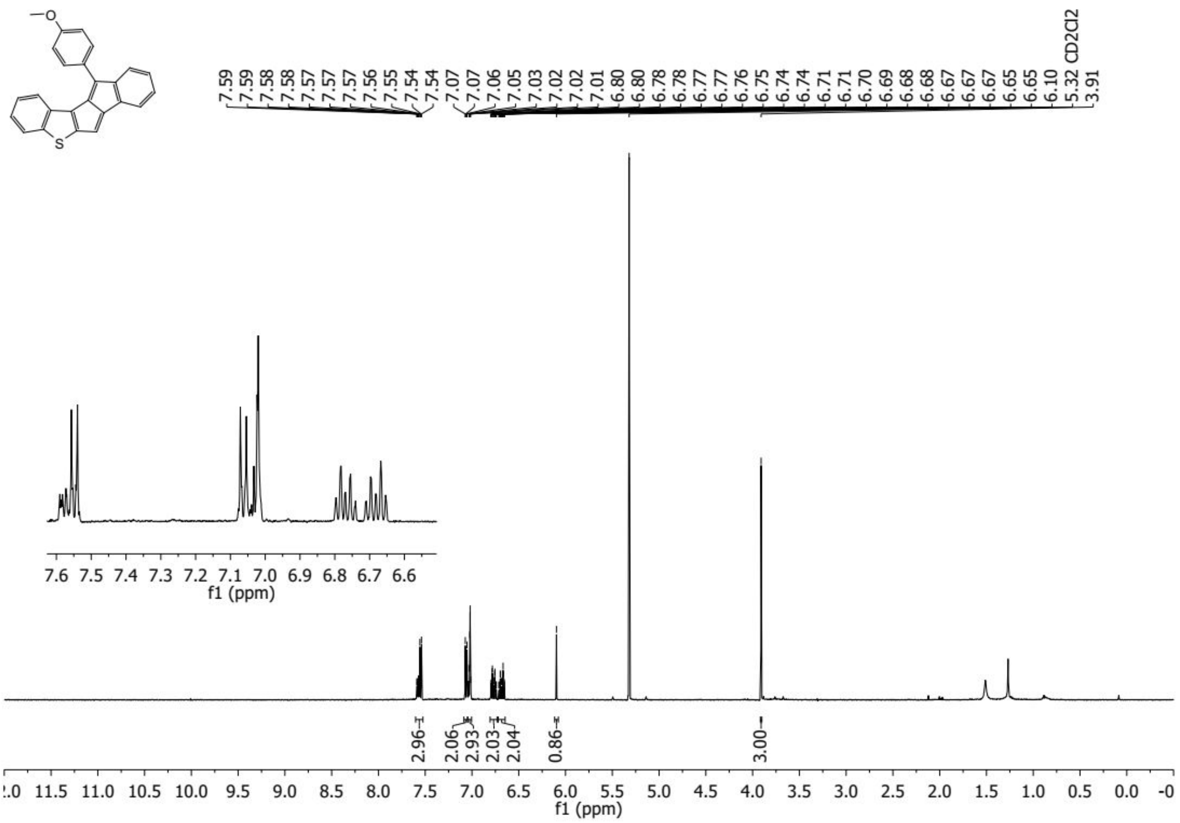

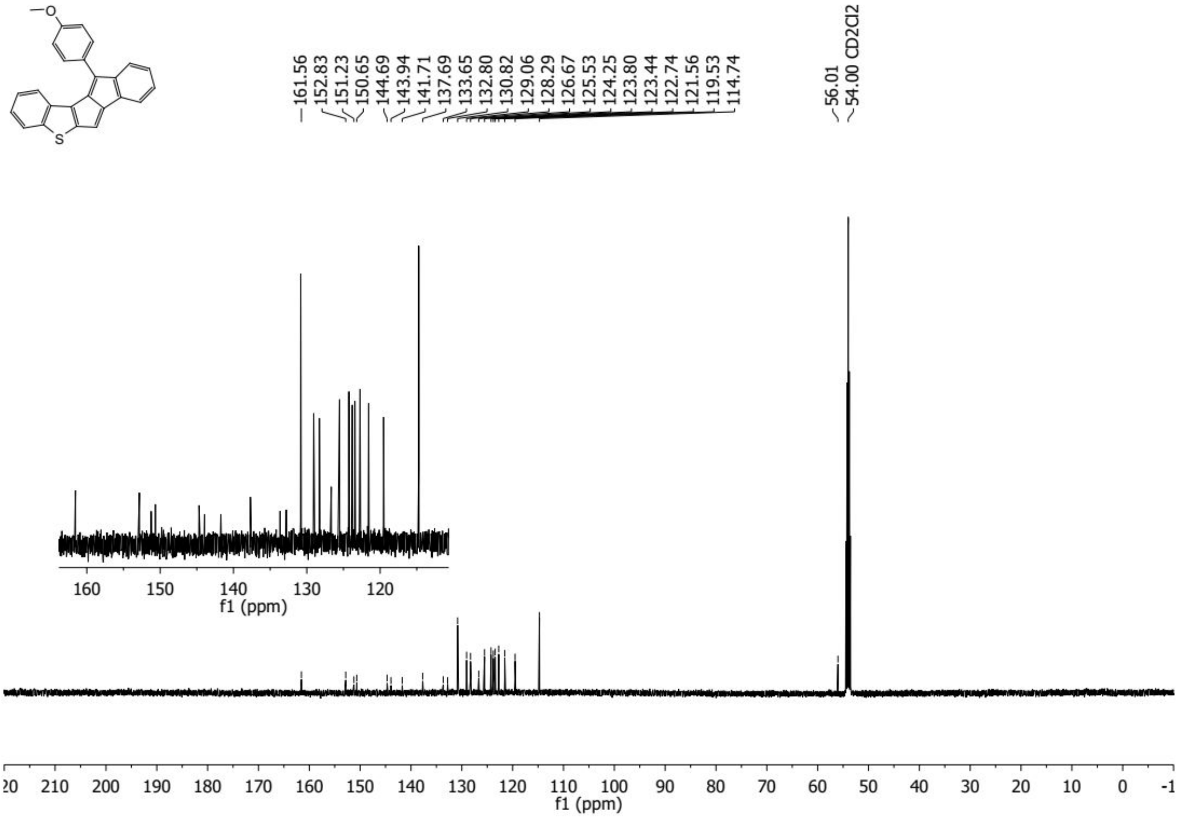

Figure S37. ^{1}H-NMR (500 MHz) (top) and ^{13}C-NMR (126 MHz) (bottom) spectra of **V**(PMP) in CD_2Cl_2.

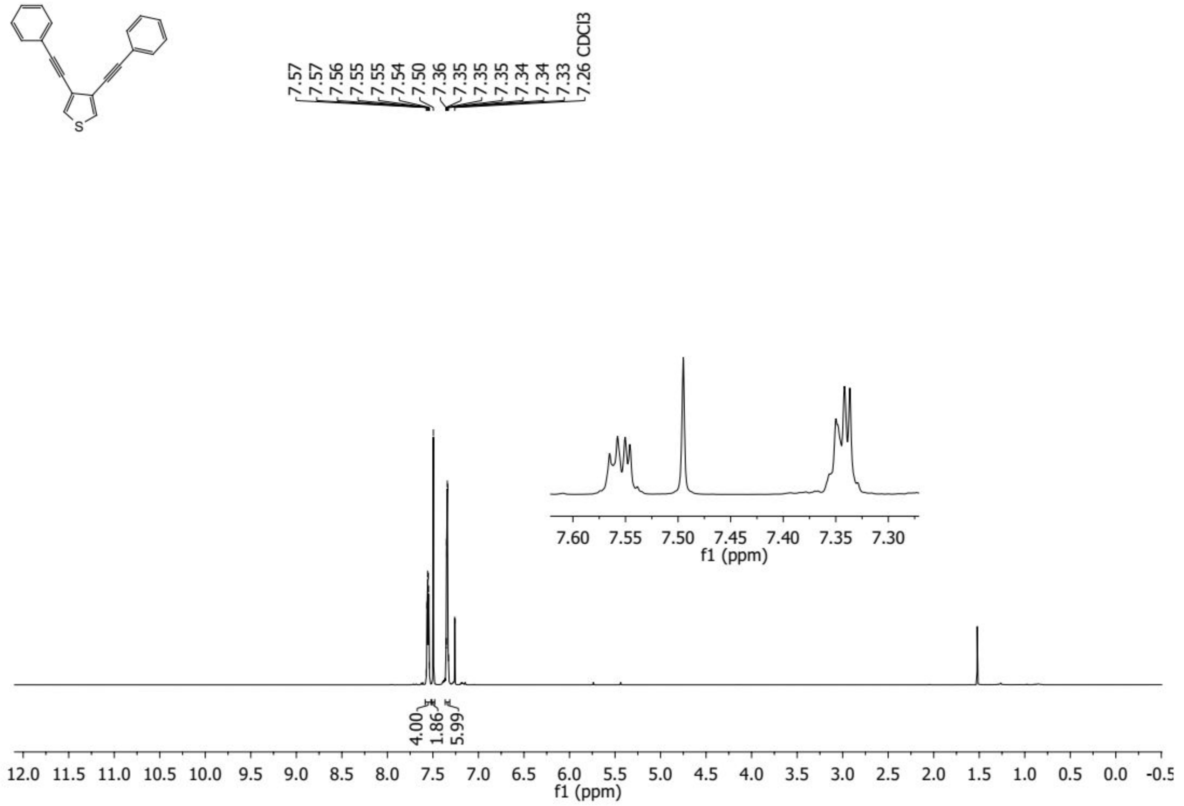

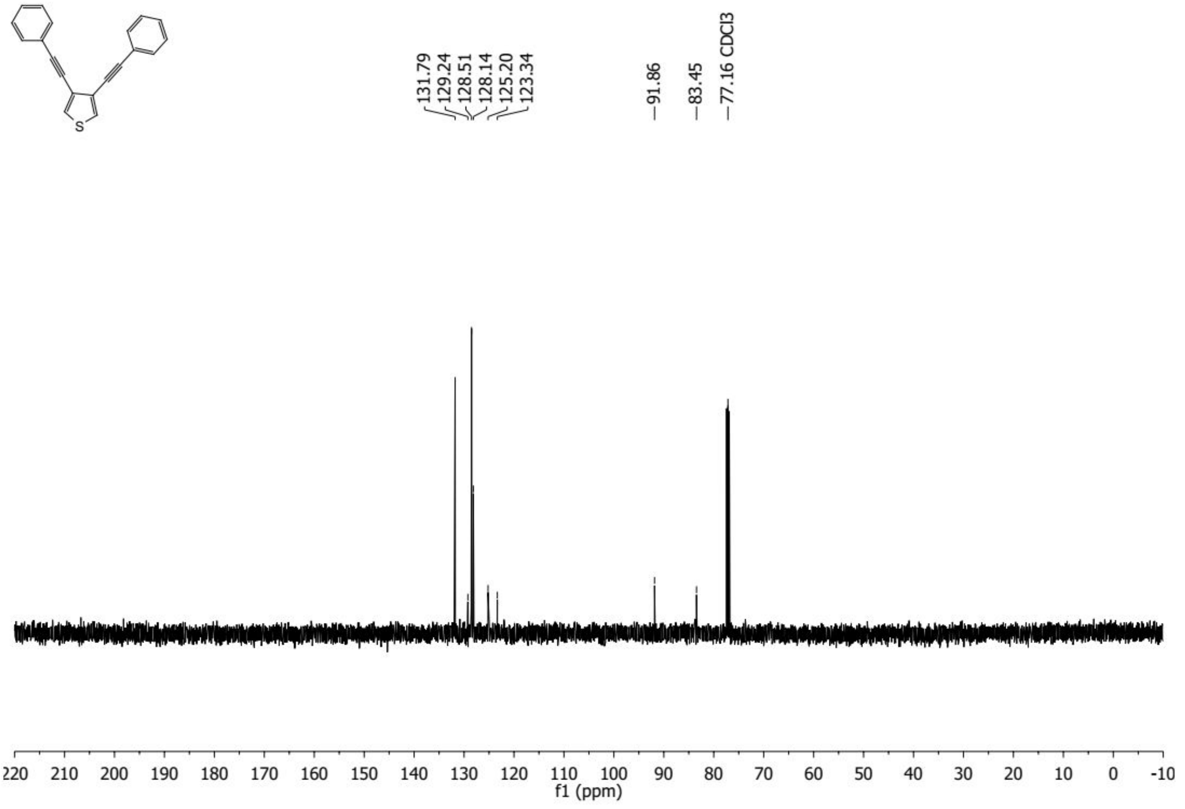

Figure S38. ^{1}H-NMR (500 MHz) (top) and ^{13}C-NMR (126 MHz) (bottom) spectra of **20** in $CDCl_3$.

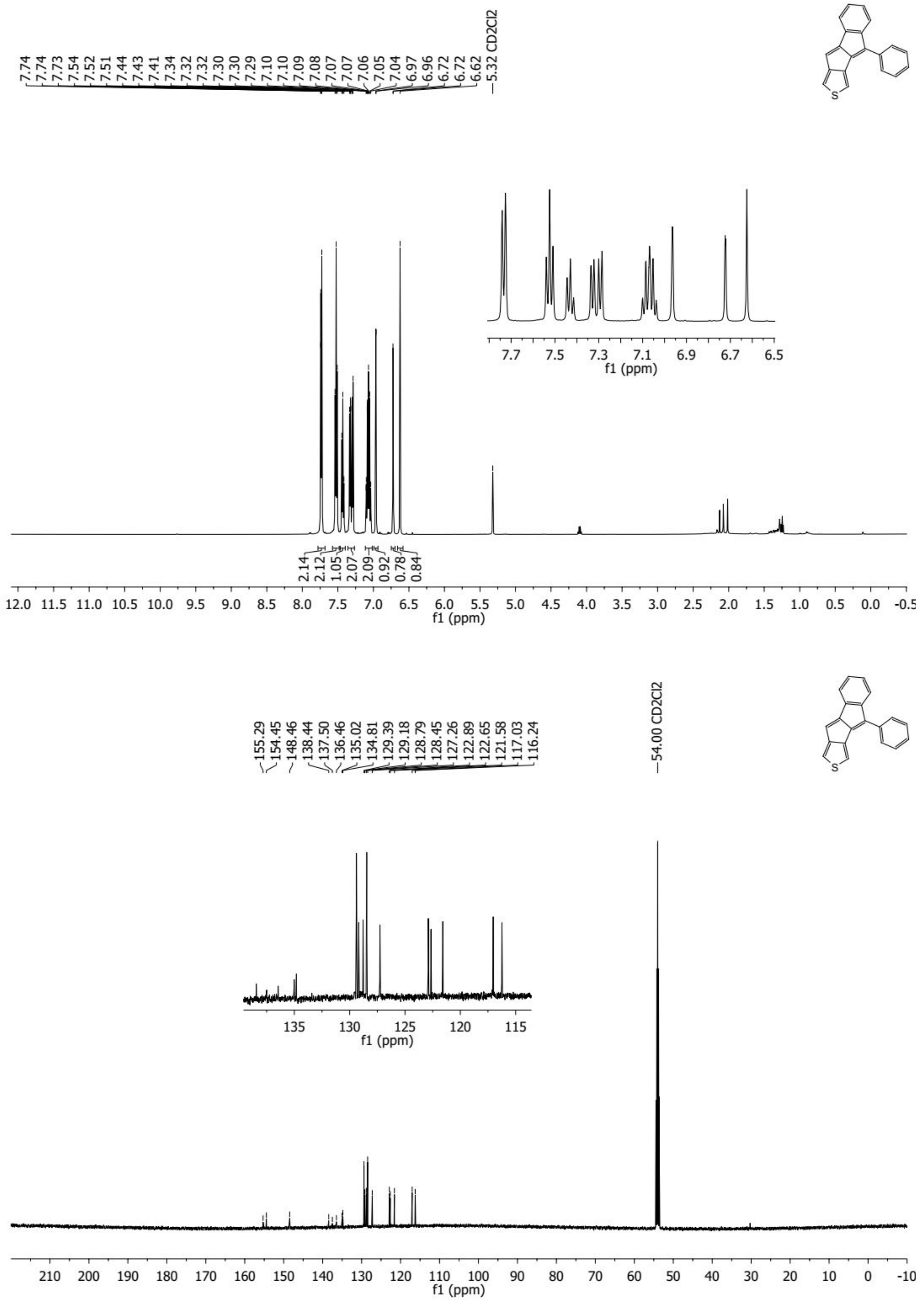

Figure S39. ^{1}H-NMR (500 MHz) (top) and ^{13}C-NMR (126 MHz) (bottom) spectra of **VI** in CD_2Cl_2.

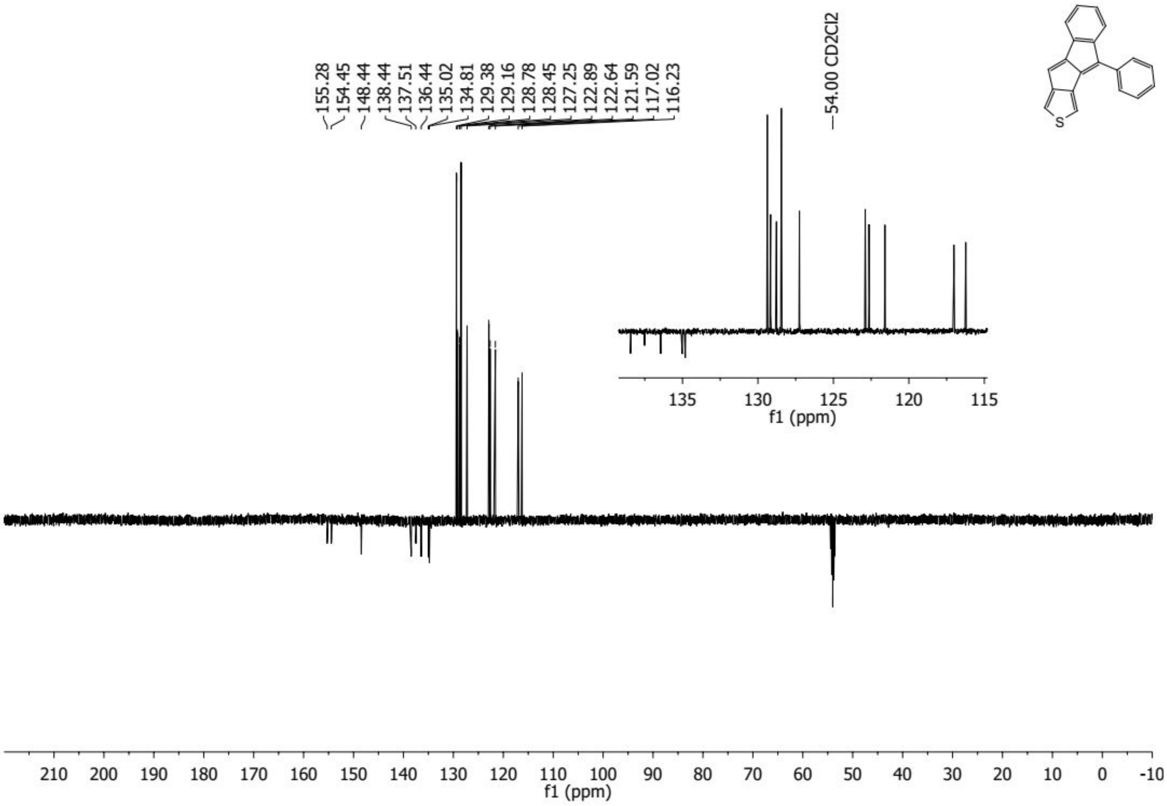

Figure S40. ^{13}C-NMR (APT) (126 MHz) spectrum of **VI** in CD_2Cl_2.

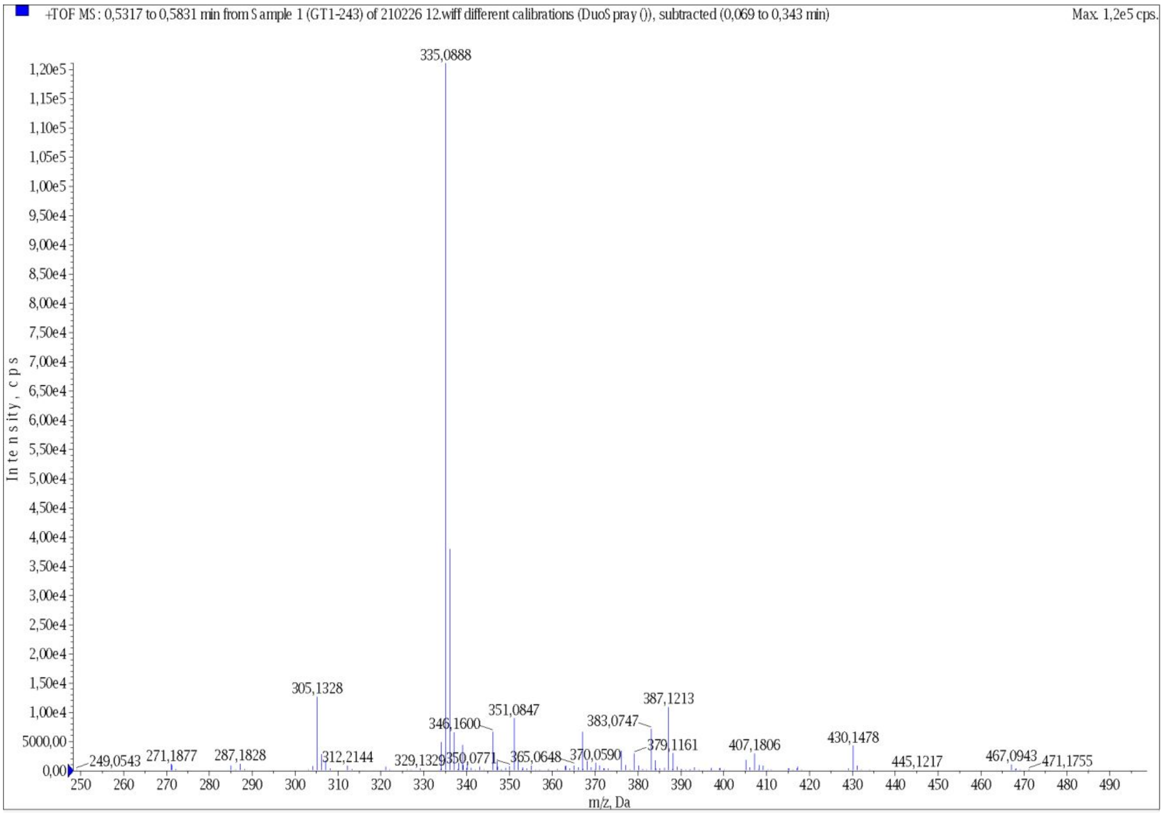

Figure S41. HRMS spectrum of the isomeric mixture of **V**(Ph).

References

1 CrystalClear SM 1.4.0 (Rigaku/MSC Inc., 2008).

2 NUMABS: Higashi, T. (1998), rev. 2002. (Rigaku/MSC Inc.)

3 Sheldrick, G. M. SHELXT - Integrated Space-Group and Crystal-Structure Determination. *Acta Cryst.* **2015**, *C71*, 3-8.

4 Spek, A. L. Single-Crystal Structure Validation with the Program PLATON. *J. Appl. Crystallogr.* **2003**, *36*, 7–13.

5 Chen, C.; Harhausen, M.; Liedtke, R.; Bussmann, K.; Fukazawa, A.; Yamaguchi, S.; Petersen, J. L.; Daniliuc, C. G.; Fröhlich, R.; Kehr, G.; Erker, G. Dibenzopentalenes from B(C6F5)3-Induced Cyclization Reactions of 1,2-Bis(phenylethynyl)benzenes. *Angew. Chem. Int. Ed.*, **2013**, *52*, 5992–5996.

6 Gaussian 09, Revision E.01, Frisch, M. J.; Trucks, G. W.; Schlegel, H. B.; Scuseria, G. E.; Robb, M. A.; Cheeseman, J. R.; Scalmani, G.; Barone, V.; Mennucci, B.; Petersson, G. A.; Nakatsuji, H.; Caricato, M.; Li, X.; Hratchian, H. P.; Izmaylov, A. F.; Bloino, J.; Zheng, G.; Sonnenberg, J. L.; Hada, M.; Ehara, K.; Toyota, K.; Fukuda, R.; Hasegawa, J.; Ishida, M.; Nakajima, T.; Honda, Y.; Kitao, O.; Nakai, H.; Vreven, T.; Montgomery, J. A.; Peralta, J. E. Jr.; Ogliaro, F.; Bearpark, M.; Heyd, J. J.; Brothers, E.; Kudin, K. N.; Staroverov, V. N.; Keith, T.; Kobayashi, R.; Normand, J.; Raghavachari, K.; Rendell, A.;Burant, J. C.; Iyengar, S. S.; Tomasi, J.; Cossi, M.; Rega, N.; Millam, J. M.; Klene, M.; Knox, J. E.; Cross, J. B.; Bakken, V.; Adamo, C.; Jaramillo, J.; Gomperts, R.; Stratmann, R. E.; Yazyev, O.; Austin, A. J.; Cammi, R.; Pomelli, C.; Ochterski, J. W.; Martin, R. L.; Morokuma, K.; Zakrzewski, V. G.; Voth, G. A.; Salvador, P.; Dannenberg, J. J.; Dapprich, S.; Daniels, A. D.; Farkas, O.; Foresman, J. B.; Ortiz, J. V.; Cioslowski, J.; Fox, D. J. Gaussian, Inc., Wallingford CT, 2013.

7 Stephens, P. J.; Devlin, F. J.; Chabalowski, C. F.; Frisch, M. J. Ab Initio Calculation of Vibrational Absorption and Circular Dichroism Spectra Using Density Functional Force Fields. *J. Phys. Chem.* **1994**, *98*, 11623–11627.

8 Krishnan, R.; Binkley, K. S.; Seeger, R.; Pople, J. Self-Consistent Molecular Orbital Methods. XX. A Basis Set for Correlated Wave Functions. *J. Chem. Phys.* **1980**, *72*, 650– 654.

9 Herges, R.; Geuenich, D. Delocalization of Electrons in Molecules. *J. Phys. Chem. A* **2001**, *105*, 3214–3220.

10 Geuenich, D.; Hess, K.; Köhler F.; Herges, R. Anisotropy of the Induced Current Density (ACID), a General Method To Quantify and Visualize Electronic Delocalization. *Chem. Rev.* **2005**, *105*, 3758–3772.

11 Gershoni-Poranne, R.; Stanger, A. The NICS-XY-Scan: Identification of Local and Global Ring Currents in Multi-Ring Systems. *Chem. Eur. J.* **2014**, *20*, 5673–5688.

12 Stanger, A. Obtaining Relative Induced Ring Currents Quantitatively from NICS. *J. Org. Chem.* **2010**, *75*, 2281–2288.

13 Stanger, A. Nucleus-Independent Chemical Shifts (NICS): Distance Dependence and Revised Criteria for Aromaticity and Antiaromaticity. *J. Org. Chem.* **2006**, *71*, 883–893.

14 Stanger, A.; Rahalkar, A. Aroma, http://schulich.technion.ac.il/Amnon_Stanger.htm.

15 Bader, R. F. W. Atoms in Molecules: A Quantum Theory, Clarendon, Oxford, **1990**

16 Bultinck, P.; Rafat, M.; Ponec, R.; Van Gheluwe, B.; Carbó-Dorca, R.; Popelier, P. Electron Delocalization and Aromaticity in Linear Polyacenes: Atoms in Molecules Multicenter Delocalization Index. *J. Phys. Chem. A* **2006**, *110*, 7642– 7648.

17 Matito, E.; Duran, M.; Solà, M. The Aromatic Fluctuation Index (FLU): A New Aromaticity Index Based on Electron Delocalization. *J. Chem. Phys.,* **2005**, *122*, 014109.

18 AIMAll (Version 17.11.14 B) TK Gristmill Software (aim.tkgristmill.com), Overland Park KS, USA, **2018**

19 Matito, E. ESI-3D: Electron Sharing Indexes Program for 3D Molecular Space Partitioning, Institute of Computational Chemistry and Catalysis, Girona, Catalonia, Spain, **2006**, https://ematito.webs.com/programs.htm

20 Frederickson, C. K.; Zakharov, L. N.; Haley, M. M. Modulating Paratropicity Strength in Diareno-Fused Antiaromatics. *J. Am. Chem. Soc.* **2016**, *138*, 16827−16838.
